# Supplementary material for: Risk stratification for hospital-acquired venous thromboembolism in medical patients (RISE): Protocol for a prospective cohort study
Source: PLoS One. 2022 May 24;17(5):e0268833. doi: 10.1371/journal.pone.0268833 (PMC9128957; doi:10.1371/journal.pone.0268833)
Supplement: S1 File — (PDF) [file pone.0268833.s001.pdf]

# RISK STRATIFICATION FOR HOSPITAL-ACQUIRED VENOUS THROMBOEMBOLISM IN MEDICAL PATIENTS: A PROSPECTIVE COHORT STUDY

---

| | |
|---|---|
| Research legislation: | Ordinance on human research with the exception of Clinical trials (HRO) [1]. |
| Type of Research Project: | Research project involving human subjects |
| Risk Categorisation: | Risk category A |

Project Leaders:

**Inselspital, Bern University Hospital:**
Dr. med. Christine Baumgartner, MAS
Department of General Internal Medicine
Inselspital, Bern University Hospital
University of Bern
Freiburgstrasse
3010 Bern
Switzerland
Phone: +41 (0)31 632 57 69
e-mail: Christine.Baumgartner@insel.ch

**Centre Hospitalier Universitaire Vaudois (CHUV):**
Dr. med. Marie Méan
Service de Médecine Interne
Centre Hospitalier Universitaire Vaudois
Rue du Bugnon 46
1011 Lausanne
Switzerland
Phone: +41 (0)21 314 11 11
e-mail: Marie.Mean@chuv.ch

Local Investigator

**Geneva University Hospital (HUG):**
Dr. med. Pauline Darbellay Farhoumand
Service de Médecine Interne Générale
Hôpitaux Universitaires de Genève
Rue Gebrielle-Perret –Gentil 4
1205 Geneva
Switzerland
Phone : +41 (0)22 372 92 03
e-mail: Pauline.Darbellay@hcuge.ch

CONFIDENTIAL
The information contained in this document is confidential and the property of the project leaders. The information may not - in full or in part - be transmitted, reproduced, published, or disclosed to others than the applicable Competent Ethics Committees and Regulatory Authorities without prior written authorisation from the sponsor except to the extent necessary to obtain informed consent from those who will participate in the study.

## CHANGE HISTORY

| Version Nr | Version date | Modified without version change | Description, comments | Control |
|---|---|---|---|---|
| 1.0 | 17.03.2020 | | Initial version | |
| 2.0 | 29.09.2020 | | Amendment : new participating site and local investigator: Pauline Darbellay Farhoumand, Geneva | |
| | | | | |
| | | | | |
| | | | | |

## PROTOCOL SIGNATURE FORM

| | |
|---|---|
| Study Title | Risk Stratification for Hospital-Acquired Venous Thromboembolism in Medical Patients: a Prospective Cohort Study |

The project leaders at the lead center/site and at the local center/site have approved the protocol version ***2.0, 29.09.2020,*** and confirm hereby to conduct the project according to the protocol, the Swiss legal requirements,[1,2] the current version of the World Medical Association Declaration of Helsinki[3] and the principles of Good Clinical Practice.

**Project leader (lead center/site)**

Site: Department of General Internal Medicine, Inselspital, Bern University Hospital, University of Bern, Freiburgstrasse, 3010 Bern, Switzerland

Name: Dr. med. Christine Baumgartner, MAS

Date: 1.12.20 Signature: C. Baumgartner

Clinic Director at lead center: Prof. Dr. med. Drahomir Aujesky

Date: 2.12.20 Signature: [illegible]

**Project leader at local center/site:**

Site Centre Hospitalier Universitaire Vaudois (CHUV), Rue du Bugnon 46, 1011 Lausanne, Switzerland

Name of Local Project Leader: Dr. med. Marie Méan

Date: 8.12.2020 Signature: [illegible]

| | |
|---|---|
| Study Title | Risk Stratification for Hospital-Acquired Venous Thromboembolism in Medical Patients: a Prospective Cohort Study |

The project leaders at the lead center/site and at the local center/site have approved the protocol version ***2.0, 29.09.2020***, and confirm hereby to conduct the project according to the protocol, the Swiss legal requirements,[1,2] the current version of the World Medical Association Declaration of Helsinki[3] and the principles of Good Clinical Practice.

**Project leader at local center/site:**

Site HUG, Geneva, Switzerland

Name of Local Project Leader: Dr. med. Pauline Darbellay Farhoumand

Date: Genève, le 27.11.2020 Signature: ______________________

**Statistician**

Andreas Limacher, PhD
Head of Statistics and Methodology
CTU Bern (Clinical Trials Unit)
University of Bern

Date: Bern, 7.12.2020 Signature: ______________________

## TABLE OF CONTENTS

CHANGE HISTORY 2
TABLE OF CONTENTS 5
GLOSSARY OF ABBREVATIONS 7
1 BACKGROUND AND PROJECT RATIONALE 8
1.1 Background 8
1.2 Relevance, Originality, and Perspectives 11
1.3 Categorization of the Study 12
2 PROJECT OBJECTIVES AND DESIGN 12
2.1 Objectives 12
2.2 Primary and secondary endpoints 13
2.2.1 Primary endpoint 13
2.2.2 Secondary endpoint 13
2.2.3 Assessment of endpoints 14
2.3 Project design 14
3 PROJECT POPULATION AND STUDY PROCEDURES 15
3.1 Project population, inclusion and exclusion criteria 15
3.2 Recruitment, screening and informed consent procedure 15
3.3 Study procedures 16
3.3.1 Planned project timeline 16
3.3.2 Patient timeline 17
3.3.3 Enrolment and Baseline 18
3.3.3.1 Baseline data collection 18
3.3.3.2 Mobility measurements 19
3.3.3.3 Substudy I: Prospective validation of the Improve Bleeding risk 20
3.3.3.4 Substudy II: Correlation between nurses'estimation of mobility and an objective motion measurement 20
3.3.3.5 Substudy III: Feasibility of a future randomized controlled trial 21
3.3.4 Follow-up visit prior to discharge 22
3.3.5 Follow-up phone call 90 days after enrolment 22
3.4 Risks and measures to minimize the risks 22
3.5 Withdrawal and discontinuation 23
4 STATISTICS AND METHODOLOGY 23
4.1 Statistical analysis plan 23
4.1.1 Sample size calculation 23
4.1.2 Statistical analyses 23
4.2 Handling of missing data 24
5 5 REGULATORY ASPECTS AND SAFETY 24
5.1 Local regulations / Declaration of Helsinki 24
5.2 Notification of safety and protective measures (HRO Art. 20) 24
5.3 5.3 Serious events (HRO Art. 21) 25
5.4 Amendments 25
5.5 End of project 25
5.6 Insurance 25

6 FURTHER ASPECTS 25
6.1 Overall ethical considerations 25
6.2 Risk-Benefit Assessment 26
6.3 Rationale for the inclusion of vulnerable participants 26
7 QUALITY CONTROL AND DATA PROTECTION 27
7.1 Quality measures 27
7.2 Data recording and source data 27
7.3 Confidentiality and coding 27
7.4 Retention and destruction of study data and biological material 28
8 FUNDING / PUBLICATION / DECLARATION OF INTEREST 28
9 REFERENCES 29

## GLOSSARY OF ABBREVATIONS

| | |
|---|---|
| *ADL* | *activities of daily living* |
| *AIC* | *Akaike information criterion* |
| *AUC* | *area under the curve* |
| *BASEC* | *Business Administration System for Ethical Committees* |
| *CHUV* | *Centre Hospitalier Universitaire Vaudois* |
| *CI* | *confidence interval* |
| *CRF* | *Case report form* |
| *DVT* | *deep vein thrombosis* |
| *FOPH* | *Federal Office of Public Health* |
| *HA-VTE* | *hospital-acquired venous thromboembolism* |
| *HRA* | *Human Research Act* |
| *HRO* | *Ordinance on Human* |
| *ICU* | *intensive care unit* |
| *LMWH* | *low molecular weight heparin* |
| *PE* | *pulmonary embolism* |
| *RAM* | *risk assessment model* |
| *ROC* | *receiver operating characteristic* |
| SSL | *Secure Sockets Layer* |
| *TPX* | *thromboprophylaxis* |
| *VTE* | *venous thromboembolism* |

# 1 BACKGROUND AND PROJECT RATIONALE

## 1.1 Background

Venous thromboembolism (VTE), defined as pulmonary embolism (PE) or deep vein thrombosis (DVT), is a feared complication among hospitalized patients, and hospital-acquired VTE (HA-VTE) is one of the leading preventable causes of in-hospital mortality.[4] The risk of VTE is particularly high in patients undergoing major surgery,[5] but hospitalization for medical illness has been identified as a risk factor *per se* for VTE,[6] with about 75% of all HA-VTE occurring in non-surgical patients.[7]

***Risk stratification to predict HA-VTE using current strategies***

Randomized-controlled trials have shown significant reductions in VTE with the use of pharmacological thromboprophylaxis (TPX) such as heparin compared to placebo in medical inpatients, although with no reduction in mortality.[8-10] The benefits of pharmacological VTE prophylaxis come at the cost of a small increase in the risk of bleeding.[8] Based on available evidence, guidelines recommend administering pharmacological TPX with low molecular weight heparin (LMWH) or fondaparinux in hospitalized medical patients at increased risk of VTE during their inpatient stay.[11,12] While VTE risk in surgical patients is dictated by the type of intervention received,[5] assessing thromboembolic risk in the population of medical inpatients is less straight forward given their heterogeneity, and the complex inclusion criteria used in previous trials are of limited help in guiding clinicians how to best identify patients who might benefit from prophylaxis. To address the challenges of VTE risk stratification in the heterogeneous population of medical inpatients and to standardize VTE risk assessment, guidelines suggest the use of a validated risk assessment model (RAM),[11,12] such as the original Geneva score,[13] the Padua or the IMPROVE score,[14,15] which incorporate a number of demographic and clinical characteristics (**Table 1**). However, current RAMs have methodological and practical limitations, including a suboptimal sensitivity to identify high risk patients,[16] items that are not available at admission (e.g. ICU stay),[15] non-uniform cut-off values to define risk groups,[15,17] or an unacceptably large number of items,[13] limiting their applicability in everyday practice.[16] In Switzerland, the simplified Geneva score (**Table 1**) has been recently developed as a simpler and thus more usable RAM.[18] In the derivation cohort, its performance was similar compared to the more complicated original Geneva score, and its sensitivity was higher compared to the Padua score.[18] A retrospective validation study has been recently performed using the Padua cohort that had been assembled in 2007 and 2008.[19] However, a prospective validation of the simplified Geneva score is lacking and urgently needed before it can be recommended for use in everyday clinical practice.

Previous studies suggest that pharmacological VTE prophylaxis is inappropriately used in medical patients: an international cross-sectional study reported that only about 40% of medical patients at high risk of VTE received appropriate prophylaxis, while on the other hand, it was inappropriately provided in almost half of all low risk patients in a Swiss multicenter study.[13,20,21] Multiple reasons have been postulated for inadequate use of VTE prophylaxis in hospitalized medical patients, including the difficulty of assessing thromboembolic risk.[22]

Immobilization is an important risk factor for VTE;[23] a post hoc analysis of a randomized study comparing the incidence of VTE in ambulatory and non-ambulatory patients treated with enoxaparin versus placebo confirmed that immobilization was associated with a 2-fold increase of HA-VTE, while the provision of pharmacological TPX reduced the risk of VTE in both groups.[24] Thus, immobilization is not only considered as a predictor in existing VTE RAMs (**Table 1**, highlighted in blue) but it is also one of the most prevalent.[13] Experts recommend providing pharmacological TPX to patients at higher VTE risk, based on existing RAMs, for 6 to 21 days, until full mobility is restored, or until discharge from hospital, whichever comes first. [11,12] Although immobilization has been recognized as a trigger for TPX administration,[25] no standardized

definition exists and its weighting varies between RAMs,[13-15,18,26] limiting greatly the usefulness of this predictor.

**Table 1. VTE risk assessment models for risk stratification in hospitalized medical patients**

| | Points | | | |
|---|---|---|---|---|
| **Score Items** | **Simplified Geneva Score[18]** | **Original Geneva Score[13]** | **Padua Score[14]** | **IMPROVE Score[15,17]** |
| Previous VTE | 3 | 2 | 3 | 3 |
| Hypercoagulable state | 2 | 2 | 3 | 2 |
| Cancer[13,14,27] | 2 | 2 | 3 | 2 |
| Myeloproliferative syndrome | | 2 | | |
| Cardiac failure | 2 | 2 | 1 | |
| Respiratory failure | | 2 | | |
| Acute infection | 2 | 2 | 1 | |
| Acute rheumatologic disorder | | 2 | | |
| Immobilization § | 2 | 1 | | 1 |
| Reduced mobility‖ | | | 3 | |
| Lower limb paralysis or paresis[27] | | | | 2 |
| Age >60 years | 1 | 1 | | 1 |
| Age >70 years | | | 1 | |
| Body mass index ≥30kg/m$^2$ | 1 | 1 | 1 | |
| Recent stroke[13] | 1 | 2 | 1 | |
| Recent myocardial infarction[13] | | 2 | | |
| Nephrotic syndrome | | 2 | | |
| Hormonal treatment | | 1 | 1 | |
| Recent travel (>6 hours) | | 1 | | |
| Chronic venous insufficiency | | 1 | | |
| Pregnancy | | 1 | | |
| Dehydration | | 1 | | |
| Recent trauma or surgery (<1 month) | | | 2 | |
| Stay in intensive or coronary care unit | | | | 1 |
| **Cut-offs[12-15,18]** | | | | |
| Low VTE risk | 0-2 | 0-2 | 0-3 | 0-1 |
| High VTE risk | ≥3 | ≥3 | ≥4 | ≥2 |

§ for the IMPROVE score, immobilization was considered if the patient was being confined to bed or chair with or without bathroom privileges for ≥7 days immediately prior to and during hospital admission;[27] for the simplified and original Geneva score, immobilization was defined as complete bedrest or inability to walk for >30min per day for ≥3 days[13]

‖ for the Padua score, reduced mobility was defined as anticipated bed rest with bathroom privileges for ≥3 days[14]

In daily hospital practice, treating physicians base their evaluation of the patient's ability to move either on observational data such as periodic nursing reports,[28-30] on nurse standardized functional assessment,[31,32] or on the own perception.[33] However, subjective physician estimation-of the patient's mobility can be inaccurate (**Figure 1**).[33]

**Figure 1. Step count by digital monitoring and physician estimation** (from: Daskivich, et al. JAMA Network Open, 2019)[33]

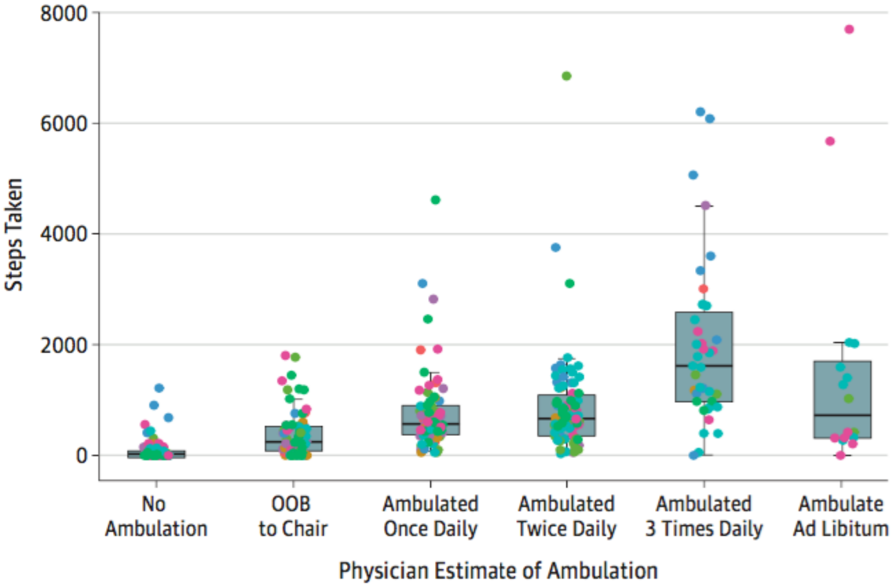


Box-and-whiskers plot showing accelerometry-measured daily step count by physician estimates in a cohort of surgical patients. The points represent individual patients. Boxes indicate the interquartile range of the steps taken, with medians marked by a vertical line within the box. Whiskers extending from the top and the bottom of the boxes indicate the range of the non-outlier values. Although physician estimates indicating higher levels of daily ambulation correlated with higher median accelerometry-measured step count, objective step counts varied widely within these categories (e.g. patients categorized by their surgeons as being out of bed [OOB] to chair had step counts ranging from 0 to 1803 steps [0-1.3 km]).

### *Risk stratification to predict HA-VTE using innovative strategies*

Recently, objective measures of mobility using accelerometry,[34] which allows the collection of objective and continuous physical activity data, improved the accuracy of mobility assessment in hospitalized patients.[35-38] Observational studies showed that medical inpatients are immobile most of the time, defined as being confined to a bed or chair.[28,37,39] In a sample of hospitalized older patients capable of walking independently prior to admission, the mean time per day spent standing or walking was 43 min.[39] Another study reported similar durations spent walking (7 min./day) and standing up (35 min./day) in 100 older patients.[40] A recent study performed in a Swiss hospital by one of the PIs confirmed that hospitalized medical patients aged ≥65 years moved very little during a hospital stay.[41] Interestingly, patient's mobility peaked during mealtimes, indicating that patients were actually able to move (**Figure 2**).

While a meta-analysis of epidemiological studies identified a 2- to 3-fold increase in VTE risk in immobilized medical patients,[23] these findings were limited by the heterogeneity in methods and inaccuracy of mobility assessment. One study with objective accelerometry-based mobility measurements in 102 patients considered HA- VTE as a secondary outcome, but the sample size was too small to draw any meaningful conclusions.[42] An objective evaluation of mobility and dose-dependent threshold to identify patients at high VTE risk has never been explored in the field of VTE prevention, neither compared to the current risk stratification strategies for HA-VTE. Whether novel, objective mobility measures such as accelerometry could predict HA-VTE, and whether incorporation of these objective measures into existing RAMs could improve their predictive ability, must therefore be examined.

**Figure 2. Average mobility per hour among patients hospitalized in an internal medicine unit at the Lausanne University Hospital (CHUV).**

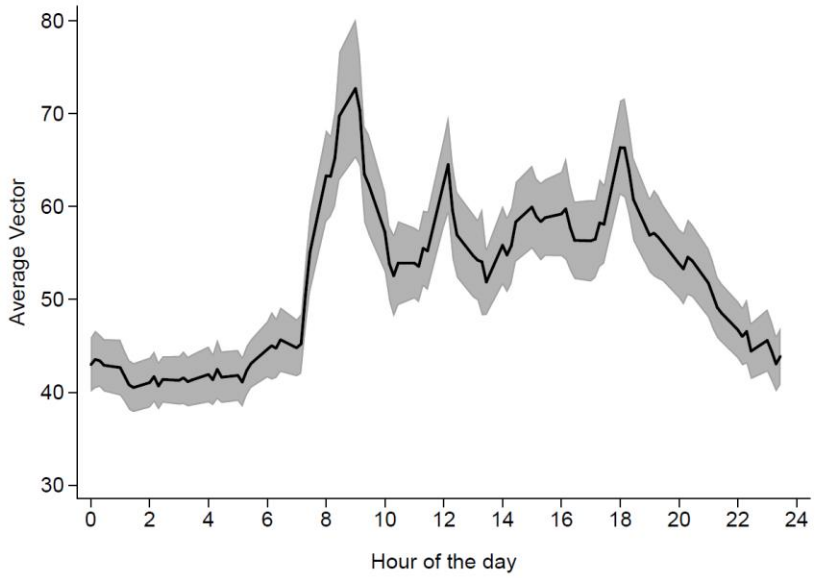


Results are shown as average and with 95% confidence interval. Average vector magnitude is an estimate reflecting total ambulatory movement over the observation period, i.e. it is a proxy for mean activity levels.

## 1.2 Relevance, Originality, and Perspectives

In accordance with a call for more research on VTE risk assessment,[12] our study **explores novel risk stratification strategies** for HA-VTE in medical inpatients using accelerometry-assessed immobility and examines the validity of a new easy-to-use RAM, the simplified Geneva score.

There is a lack of objective information on mobility and its level-dependent impact on VTE in medical inpatients, limiting the ability of physicians to estimate the degree of a patient's mobility or immobility to inform the decision on TPX prescription. Thus, the use of a tracking device to record and monitor daily steps for measuring mobility during hospital stay is **innovative** and may reflect patient mobility more accurately than standard subjective methods of mobility assessment. If our study can establish an objectively measured immobility level at which inpatients would be at higher risk of HA-VTE, increasing patient mobility above this threshold could be a simple and safe novel VTE prevention strategy.

Moreover, our project will provide the **first prospective head-to-head comparison** of validated VTE RAMs. The current uncertainty about optimal provision of TPX and the underuse of RAMs due to their complexity[22] is reflected by the fact that only about 40% of high risk patients receive appropriate TPX,[20] while it is inappropriately provided in almost half of all low risk patients,[21] resulting in unnecessary bleeding events and costs. Our results will provide a clear guidance for physicians about optimal VTE risk assessment and thus have the potential to **facilitate and improve VTE prevention** and reduce HA-VTE and associated deaths in medical inpatients.

To our knowledge, this will be the largest cohort of medical inpatients with accelerometry-assessed mobility, generating data for numerous secondary analyses. For example, the association between objective patient mobility and hospital length of stay could be investigated, or adequacy of TPX prescription in relation to current guideline recommendations could be assessed as a basis for a quality improvement intervention. Importantly, this project will provide preliminary data to assess the feasibility and inform the design of **a future randomized trial** comparing the impact of different VTE prevention strategies on clinically relevant outcomes. Such a trial is urgently needed to fill an important gap in knowledge, whether pharmacologic

VTE prophylaxis confers a net clinical benefit in medical inpatients at high risk of VTE. It will address the call from experts for additional research to clarify the risk-benefit ratio of inpatient pharmacologic TPX in acutely ill medical inpatients, while providing a clear guidance about baseline VTE risk assessment.[12,43,44] The data generated by this project will be used to estimate the recruitment potential and feasibility. In addition, the results of this study will help to identify the most accurate RAM for defining the eligibility criteria for this future trial to select patients at high risk of VTE which are most likely to benefit from VTE prophylaxis. The identification of a mobility threshold above which VTE risk decreases will inform the design of an intervention to increase patient mobility as a novel non-pharmacological VTE prevention strategy. Although immobilization is a key modifiable VTE risk factor, until today no study, in Switzerland or elsewhere, has tested the impact of an interventional program aiming at increasing the patient's mobility instead of prescribing a pharmacological TPX to prevent HA-VTE.

In conclusion, our project has the potential to generate **innovative knowledge about VTE prevention and risk stratification**, and thus to **improve the quality of care** of medical hospitalized patients.

### 1.3 Categorization of the Study

This study is a risk category A study according to the Swiss law (HRO Art. 7) as the measures for collecting personal data (including interviews of patients and health care personnel, review of medical records, accelerometry) only entail minimal risks and burden.

## 2 PROJECT OBJECTIVES AND DESIGN

### 2.1 Objectives

The ***broad objective*** of this prospective study is to improve VTE prevention strategies in hospitalized medical patients by prospectively validating a novel Swiss RAM, the simplified Geneva score, and by developing a new, objective, and innovative definition of patient immobilization in the hospital using accelerometry.

The specific objectives of this project are divided in two work packages:

**Objectives of work package 1: <u>Evaluate current VTE risk prediction strategies</u>**

**1)** to prospectively validate the simplified Geneva score as a RAM to predict HA-VTE (primary objective)

***Assumption:*** *the novel, easier-to-use simplified Geneva score will be able to accurately detect medical inpatients at risk of HA-VTE (primary hypothesis)*

**2)** to compare the prognostic performance of the simplified Geneva score to discriminate between low and high VTE risk patients with previously validated RAMs (original Geneva, Padua, and IMPROVE score)

***Assumption:*** *the novel, easier-to-use simplified Geneva score will be at least as accurate as previously validated RAMs in detecting medical inpatients at risk of HA-VTE*

**Objectives of work package 2: <u>Assess novel VTE risk prediction strategies using objective measures of mobility</u>**

**1)** to assess whether objective accelerometry-assessed immobilization predicts the risk of HA-VTE and to compare it to the standard subjective method to assess immobilization (physician perception)

***Assumption:*** *objective, accelerometry-assessed immobilization will be more accurate in predicting the risk of HA-VTE than subjective physician perception.*

**2)** to compare the predictive performance of the simplified Geneva score using the standard subjectively-assessed definition of immobilization (i.e. physician perception) versus using objective accelerometry-assessed mobility measures

***Assumption:*** *the incorporation of accelerometry-assessed immobility into the simplified Geneva risk score will improve its prognostic performance*

## 2.2 Primary and secondary endpoints

### 2.2.1 Primary endpoint

The primary outcome will be symptomatic, objectively confirmed fatal and non-fatal HA-VTE, including symptomatic distal and proximal DVT and PE up to 90 days after hospital admission.

The objective diagnostic criteria for a PE diagnosis will be based on available radiographic or autopsy reports, and will include a new intraluminal filling defect on computed tomography pulmonary angiography (CTPA) or pulmonary angiography; a perfusion defect involving at least 75% of a segment, with corresponding normal ventilation (i.e., high probability lung scan); the confirmation of a new PE on autopsy, or an objectively confirmed proximal DVT with clinical signs and symptoms of PE.[45] The objective diagnosis of DVT will be the non-compressibility of a venous segment on compression ultrasonography or an intraluminal filling defect on contrast venography.[45] Because iliac veins and the inferior vena cava may be technically difficult to compress, additional diagnostic criteria for iliac and caval DVT will also include abnormal duplex flow patterns compatible with thrombosis or an intraluminal filling defect on spiral computed tomography or magnetic resonance imaging venography.[46] We will consider both proximal and distal symptomatic DVTs. In line with previous studies on VTE prophylaxis and given similarities in some risk factors and outcomes,[27,47] symptomatic upper extremity DVT will also be considered as a study outcome, although its incidence is expected to be low.[47] VTE events diagnosed during the first 48 hours of hospitalization will not be considered as a primary outcome for this study in order to rule out pre-existing VTE that occurred prior to hospital admission.[48]

### 2.2.2 Secondary endpoint

As secondary outcomes we will collect:

- symptomatic, objectively confirmed fatal and non-fatal HA-VTE during hospitalization
- all-cause mortality during hospitalization and up to 90 days after admission
- major bleeding during hospitalization and up to 90 days after admission. Major bleeding will be defined as fatal bleeding, symptomatic bleeding at critical sites (intracranial, intraspinal, intraocular, retroperitoneal, intraarticular, pericardial, or intramuscular with compartment syndrome), or bleeding with a reduction of hemoglobin of at least 20 g/L or bleeding leading to transfusion of 2 or more units of packed red blood cells according to the definition of the International Society on Thrombosis and Haemostasis.[49]
- clinically relevant non-major bleeding during hospitalization and up to 90 days after admission. Clinically relevant non-major bleeding will be defined as overt bleeding that does not meet criteria for major bleeding but is associated with a medical intervention, unscheduled physician contact (visit or telephone call), or pain or impairment of activities of daily life[50]
- Patient autonomy in the activities of daily living at discharge and at 90 days after admission using the modified Barthel Index
- Length of hospital stay, defined as the time/date of discharge minus time/date of admission at the hospital ward.
- Subsequent hospitalization (readmission) up to 90 days after admission.

### 2.2.3 Assessment of endpoints

To obtain information on the occurrence and timing of outcome events, study personnel will conduct patient visits on the day prior or the day of discharge, and will contact patients, family members, and/or primary care physicians by phone at 90 days after admission.[13,46]

Medical records will be collected and reviewed to obtain information about the date/type/circumstances of medical outcome events (HA-VTE, major and clinically relevant non-major bleeding, death). In patients who experienced HA-VTE, radiographic reports and images will be obtained. For patients who died, the cause of death based on medical reports, death certificates, and autopsy reports (if available) will be recorded.

Patient's autonomy in the activities of daily living will be assessed using the modified Barthel index in a face-to-face interview at hospital discharge, and by phone at 90 days after admission. The modified Barthel Index has been reported as being the most accurate scale to assess activities of daily living (ADL) and has thus been widely used as a measure of autonomy.[51] The modified version improves the internal consistency and provides a better discrimination of functional ability compared to the original version.[51] The patient's ability to perform different ADLs will be rated as follows: fully independent, with minimal or moderate help, attempts task but unsafe, and unable to perform. The maximum point score is 100; a total modified Barthel Index point score of 0-20 suggests total, 21-60 severe, 61-90 moderate, and 91-99 slight dependence. A point score of 100 indicates that the patient is independent of assistance from others.

To assess length of hospital stay, admission and discharge times/dates will be obtained from hospital charts. The number of subsequent hospitalizations up to 90 days after admission will be obtained through patient interview during the follow-up phone call, interview of the patient's primary care physician, or hospital chart review.

All medical outcome events (HA-VTE, major and clinically relevant non-major bleeding, and death) will be adjudicated by a committee of 3 independent clinical experts. Based on available information, death will be adjudicated as PE-related, due to another cause, or due to an undetermined cause (if the cause of death is undetermined based upon the clinical information, or if no or insufficient information is available to determine the cause of death). Death will be considered PE-related in the following situations: a) autopsy-confirmed PE in the absence of another more likely cause of death, or b) objectively confirmed PE within the last 48 hours before death in the absence of another more likely cause of death, or c) PE is not objectively confirmed, but is most likely the main cause of death (personal communication from the International Society of Thrombosis and Haemostasis working group for the Classification of the Cause of Death in VTE Studies). For death due to a cause other than PE, the cause will be recorded using the World Health Organization definitions (bleeding, acute coronary syndrome, ischemic stroke, left ventricular failure, sudden death without known etiology, cancer, and other).[52] The final adjudication will be based on the committee's consensus.

## 2.3 Project design

We will conduct a multicenter national non-interventional prospective cohort study including consecutive consenting adult patients admitted to general internal medicine at ≥3 large-volume Swiss hospitals. VTE risk will be assessed at admission using the simplified Geneva score, and 3 other validated RAMs. Mobility will be measured during the hospitalization using accelerometry. Patients will be followed for the occurrence of HA-VTE, with a follow-up phone call at 3 months after admission (**Figure 3**).

**Figure 3. Prospective cohort study on risk stratification for hospital-acquired VTE in hospitalized medical patients**

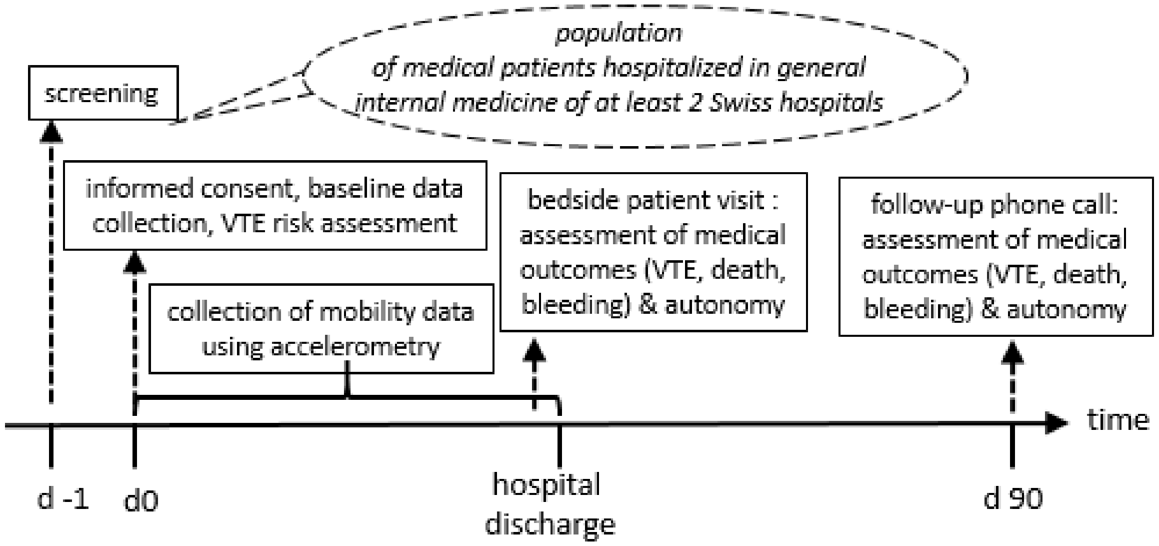


# 3 PROJECT POPULATION AND STUDY PROCEDURES

## 3.1 Project population, inclusion and exclusion criteria

Overall, 1350 consecutive adult patients hospitalized for acute illness in general internal medicine will be invited to participate in this study upon admission if they meet the following eligibility criteria:

- **Inclusion criteria:**
  1) age ≥18 years
  2) admitted for hospitalization >24 hours on a general internal medicine ward
  3) informed consent as documented by signature

- **Exclusion criteria:**
  1) need for therapeutic anticoagulation (e.g., atrial fibrillation)
  2) life expectancy <30 days
  3) insufficient proficiency of the German or French language
  4) unwilling to provide informed consent
  5) prior enrolment in the study

For participants who are unable to give informed consent due to mental illness or cognitive impairment, permission to participate in the study will be obtained from a legally authorized representative. We will not exclude these patients, because the risk of VTE and immobilization and the associated adverse outcomes are particularly high in the elderly,[53,54] where cognitive impairment is more prevalent.

## 3.2 Recruitment, screening and informed consent procedure

Consecutive hospitalized medical patients will be screened for recruitment at the General Internal Medicine wards of at 3 large Swiss hospitals. Study nurses will screen newly admitted patients (n≈12-15 per hospital/day) on a daily basis for eligibility. Reasons for exclusion will be recorded for all patients. Potentially eligible patients will receive the participant information sheet. The investigators or delegated study members will explain to each participant the nature of the study, its purpose, the procedures involved, the expected duration, the potential risks and

benefits and any discomfort it may entail. Potential participants will be informed that the participation in the study is voluntary and that they may withdraw from the study at any time and that withdrawal of consent will not affect subsequent medical assistance and treatment. They will be informed that their medical records may be examined by authorised individuals other than their treating physician. The potential study participants will have time to read the participant information, and they will have the opportunity to ask questions. Ample time (up to 24 hours) will be given to study candidates to consider their participation. The investigator or his/her delegate will ask eligible patients who are interested in study participation to provide informed consent and sign and date the approved consent form. The consent form will also be signed and dated by the investigator or delegated study team member after the participant has signed, and it will be retained as part of the study records. A copy of the signed document will be given to the study participants.

Screening of patients who are admitted on weekends or holidays will occur on the next working day. To assess generalizability of enrolment, demographic data (age, year of birth, sex) without any identifying, patient-specific information and the reasons for exclusion will be recorded for all patients who fulfill one or more exclusion criteria or who do not consent to participate.

No payment or compensation will be given to study participants.

Given that >9'000 patients are hospitalized at the general internal medicine wards of two of the participating hospitals annually, and assuming that 70% of the patients will be eligible and >50% will agree to participate based on pilot data and previous experience, achieving the target sample size of 1350 over a recruitment period of 15 months is highly feasible. Patient enrolment will be continuously monitored to ensure that recruitment goals are met.

If the observed enrolment rates should be unexpectedly lower than anticipated, we will reassess the recruitment plan and alter the recruitment strategy, e.g. by considering additional study sites or by extension of the recruitment phase to achieve the target sample size.

## 3.3 Study procedures

### 3.3.1 Planned project timeline

The overall duration of the study phase of this project is planned to be 18 months (we plan a 15 months recruitment period, and 3 months follow-up period; see **Table 2**). Each patient will be followed for 3 months after inclusion.

**Table 2. Planned Project Timeline**

| **Tasks** **Project Month** | 5 | 6 | 7 | 8 | 9 | 10 | 11 | 12 | 1 | 2 | 3 | 4 | 5 | 6 | 7 | 8 | 9 | 10 | 11 | 12 | 1 |
|---|---|---|---|---|---|---|---|---|---|---|---|---|---|---|---|---|---|---|---|---|---|
| **STUDY PHASE (18 months)** | | | | | | | | | | | | | | | | | | | | | |
| Patient recruitment | | | | | | | | | | | | | | | | | | | | | |
| Patient follow-up | | | | | | | | | | | | | | | | | | | | | |
| **CLOSE OUT (3 months)** | | | | | | | | | | | | | | | | | | | | | |
| Data cleaning | | | | | | | | | | | | | | | | | | | | | |
| Data analysis | | | | | | | | | | | | | | | | | | | | | |
| Preparation of reports and manuscripts | | | | | | | | | | | | | | | | | | | | | |
| Dissemination of results | | | | | | | | | | | | | | | | | | | | | |

### 3.3.2 Patient timeline

**Table 3. Timeline of patient enrolment and schedule of assessments**

| Study Period | Screening | Enrolment & Baseline | | Follow-up | Close-out |
|---|---|---|---|---|---|
| **Visit / Follow-up phone call** | **Screening** | **1** | | **2** | **3✆** |
| **Time (day with allowed visit window)** | **d ≤ -1** | **d0** | **d1 (1-3)** | **dx** | **d90 (85-95)** |
| **Description of time point** | **Hospital admission[1]** | | **Day after inclusion** | **Day prior to discharge[2]** | **End of follow-up** |
| Eligibility screen | x | | | | |
| Oral and written information | x | | | | |
| Written informed consent | | x | | | |
| ***Baseline characteristics*** | | | | | |
| Contact information | | x | | | |
| Demographic characteristics | | x | | | |
| Items of the RAMs | | x | | | |
| Comorbidities | | x | | | |
| Contraindications to pharmacological VTE prophylaxis | | x | | | |
| Laboratory findings | | x | | | |
| Medications at admission | | x | | | |
| Treatments since admission | | x | | x | x |
| Autonomy: modified Barthel Index | | x | | x | x |
| Pressure sore risk: Braden scale | | x | | | |
| ***Mobility measurements*** | | | | | |
| Distribution of accelerometer & instructions | | x | | | |
| Accelerometry | | | | | |
| Physician's estimation of physical activity level | | | x | | |
| Ability to walk | | x | | | |
| ***Feasibility Substudy[3]*** | | | | | |
| Consent for hypothetical RCT | | | | ***x*** | |
| ***Medical Outcomes*** | | | | | |
| Symptomatic HA-VTE | | | | x | x |
| All-cause mortality | | | | x | x |
| Major bleeding | | | | x | x |
| Clinically relevant non-major bleeding | | | | x | x |
| ***Other study data*** | | | | | |
| Length of hospital stay | | | | x | |
| Discharge location | | | | x | |
| Duration of VTE prophylaxis | | | | x | |
| Subsequent hospitalizations | | | | | x |

[1] Enrolment and baseline visit may take place within 72 hours of admission

[2] The final visit during the hospital stay will take place the day prior to discharge or on the day of discharge.

[3] Recruitment feasibility for a future hypothetical randomized controlled trial (RCT) will be assessed in patients from one study site (Inselspital) who meet potential eligibility criteria for this hypothetical trial.

### 3.3.3 Enrolment and Baseline

#### 3.3.3.1 Baseline data collection

After study inclusion, study personnel will collect the participants' contact details (name, date of birth, address, phone number, e-mail, contact details of the primary care physician and of a surrogate). This is necessary in order to contact the patients for the follow-up phone calls (or a surrogate in case the patient cannot be reached or has cognitive impairment) and to collect outcome data. These identifying data will be securely stored at the local study site and not be entered in the research database. For all eligible and consenting participants, study personnel will prospectively collect baseline data to describe the study population and for use as adjustment variables in statistical analyses. Following baseline data will be collected (**Table 4**): a) demographic data; b) all items of the simplified and original Geneva score, IMPROVE score, and Padua score (**Table 1**); c) comorbidities d) potential contraindications for pharmacological TPX; e) laboratory findings that are relevant concerning VTE or bleeding risk; f) medications at admission with a potential antithrombotic effect; g) treatments during the current hospital stay, including start date and type of TPX, defined as any use of pharmacological (unfractionated or low-molecular-weight heparin, fondaparinux, or direct oral anticoagulants) or mechanical (intermittent pneumatic compression devices or compression stockings) TPX, as well as other treatments potentially affecting risk of HA-VTE or bleeding; h) information on patient's autonomy.

**Table 4. Baseline data collection**

| |
|---|
| **Demographic characteristics**<br>Sex, year of birth, date of admission, date of inclusion, body weight, height, setting prior to admission |
| **Items of the risk assessment models**<br>Previous VTE, hypercoagulable state/thrombophilia, active cancer, history of cancer within last 5 years, myeloproliferative syndrome, cardiac failure, respiratory failure, acute infection, rheumatologic disorder, immobilization (bed rest with bathroom privileges) ≥72 hours, estimated immobilization >7d, stroke (and date of event), myocardial infarction (and date of event), recent (≤1 month) trauma or surgery (and date of event), ongoing hormonal treatment, lower extremity paralysis/paresis, stay in the intensive care unit / intermediate care unit, nephrotic syndrome, recent travel (>6 hours), chronic venous insufficiency, pregnancy, dehydration |
| **Other potential VTE risk factors**[55]<br>Heart rate, body temperature, leg edema, active tobacco use |
| **Comorbidities**[56]<br>History of myocardial infarction, congestive heart failure, peripheral vascular disease, cerebrovascular disease, dementia, chronic obstructive pulmonary disease, connective tissue disease, peptic ulcer disease, liver disease, diabetes mellitus, hemiplegia, chronic kidney disease, localized solid tumor, metastatic solid tumor, leukemia, lymphoma, AIDS, gastroduodenal ulcer, history of bleeding, inflammatory bowel disease, blood dyscrasias, anemia; number of comorbidities; risk of bedsores (using the Braden scale) |
| **Contraindications to pharmacological VTE prophylaxis**<br>Known hypersensitivity to heparin, history of heparin induced thrombocytopenia, liver failure, active non-major or major bleeding (and date of event), hemorrhagic transformation of acute ischemic stroke (and date of event) |
| **Laboratory findings**<br>Platelet count, international normalized ratio (INR), serum creatinine, hemoglobin, leukocytes, CRP, D-Dimer, fibrinogen |
| **Medications at admission**<br>aspirin, other antiplatelet therapy (clopidogrel, prasugrel, ticagrelor), nonsteroidal antiinflammatory drugs (NSAIDs) |
| **Treatments during hospital stay**<br>Pharmacological thromboprophylaxis: type (low molecular weight heparin, unfractionated heparin, fondaparinux, other), start date; mechanical VTE prophylaxis (lower extremity compression stockings/bandages, intermittent pneumatic compression devices); red blood cell transfusions (and date), central venous catheter |

| |
|---|
| **Autonomy**<br>Modified Barthel Index; location of eating, eliminating urine or stool, and washing |

Comorbidities will be collected according to the Charlson Comorbidity Index.[56]

Autonomy in the activities of daily living prior to hospitalization will be assessed at admission using the modified Barthel Index in a face-to-face interview (for description of the modified Barthel Index, see section 2.2.3). For patients with cognitive impairment or confusion, the level of autonomy before hospitalization will be assessed by interviewing their relatives or caregivers, in face-to-face interviews or by phone call.

Data will be collected from electronic health records, except for information such as data on contact details, mobility, paresis/paralysis, recent travel, dehydration, leg, edema, and patient autonomy, which will be collected at the patient bedside (from the patient and/or nurse in charge) by trained study nurses.

At baseline, the simplified and original Geneva score, the IMPROVE score, and the Padua score will be calculated and patients will be categorized as high or low VTE risk according to each RAM (**Table 1**). Treating physicians will not be informed of the patients' scores, and no specific recommendations concerning the use of a RAM or TPX will be made. Thus, the use of TPX will not be influenced by the study.

### 3.3.3.2 Mobility measurements

Because immobilization criteria defined in each RAM consider the patient's ability or inability to move and its duration (**Table 1**), we will objectively assess periods of mobility in all eligible and consenting patients using a wrist-worn tri-axis accelerometer (GENEActiv Original, ActivInsights Ltd, UK, https://www.activinsights.com/actigraphy/geneactiv-original/), parametrized at 50 Hz. These accelerometers have been shown to provide a reliable and valid measurement of physical activity in adults[57] and hospitalized patients.[34,38] The devices will be provided to the patients immediately after inclusion and patients will choose on which wrist they prefer to wear the device. Previous studies have shown that measuring site does not influence measurement.[58] Patients will be asked to wear the device continuously (day and night, including showering) until hospital discharge or transfer to another department (e.g. intensive care, surgery unit). Accelerometry data will be extracted and analyzed using the GGIR package for R (version 1.11 or later) [59]. A valid day will be defined as at least 10 hours of daytime wear, and at least 24 hours of valid data will be required for analysis.[60] In the analysis, we will consider the following measurements: minutes/day in different type of activities, no activity or sleep; total minutes during day spent active/inactive; mean acceleration in miliG/vector.

Physical activity raw data will be further processed using the open source GENEAclassify R-package (https://cran.r-project.org/web/packages/GENEAclassify/GENEAclassify.pdf) in order to obtain the number of steps taken per day, the mean cadence, and the maximum gait speed (m/s). We will estimate the percentage of time of patient's mobility, using a cut-off of <4 steps and ≥4 steps taken per minute to define periods of immobilization and mobilization respectively, as previously reported in a study of medical inpatients.[37]

At baseline, patients will be asked about their ability to walk, i.e. whether they are able to walk independently, with assistance from one or two persons, with or without mobility aids, or if they are not able to walk. In addition, we will assess from medical records whether physical therapy has been prescribed for mobilization.

On the second day of admission, study nurses will ask the treating physicians to judge whether their patients fulfill the different immobilization criteria as defined in each RAM (**Table 1**):

- for the simplified and original Geneva score: complete bedrest or inability to walk for >30min per day for ≥3 days[13]
- for the Padua score: anticipated bed rest with bathroom privileges for ≥3 days[14]
- for the IMPROVE score: patient is confined to bed or chair with or without bathroom privileges for ≥7 days immediately prior to and during hospital admission.[27]

The treating physicians will also be asked to subjectively estimate the patient's mobility level in standardized terms (i.e., no ambulation, out of bed to chair, out of bed to ambulate once daily, twice or 3 times daily, or ambulate ad libitum).[33] Physicians will be contacted on the second day of hospitalization rather than on admission because the decision to prescribe TPX is most likely already made on the second hospital day and thus less likely to be influenced by questions on the patient's mobility status. Prescriptions of specific ambulation regimens or physical therapy will be left at the discretion of the treating physician.

#### 3.3.3.3 Substudy I: Prospective validation of the IMPROVE bleeding risk score

TPX related-bleeding risk assessment in medical inpatients is a challenge. The IMPROVE bleeding risk score consisting of 11 demographical, clinical, and laboratory items was derived and validated to predict in-hospital bleeding in medical inpatients upon admission.[61,62] However, in the external validation cohort, bleeding as the main outcome was retrospectively identified based on ICD-10 codes of major bleeding, resulting in limited accuracy of the outcome measurement. In addition, potentially relevant non-major bleeding events were missed.[62] Thus, external validation with prospective and accurate assessment of bleeding outcomes has not been performed yet, and whether this model (www.outcomes-umassmed.org/improve/) may help to adequately assess the risk of bleeding prior to TPX prescription remains to be evaluated.

We aim to prospectively validate the IMPROVE bleeding risk score in this Swiss cohort of hospitalized medical patients. In addition, we will review prophylaxis practice patterns and assess the effects that various prophylactic regimens have on the risk of bleeding and the performance of the score. Finally, we will calculate the IMPROVE VTE risk score[27] in addition to the IMPROVE bleeding risk score to assign a risk-benefit category to all patients in our cohort.

#### 3.3.3.4 Substudy II: Correlation between nurses'estimation of mobility and an objective motion measurement

VTE RAMs for inpatients frequently include immobilization as a variable. However, the reliability and inter-individual variability of this assessment by nurses or doctors is not well established. The Braden scale has been validated to identify hospitalized patients at risk of pressure sores (**Figure 3**).[41,63] This scoring system includes 6 items with a total score ranging from 0 to 23. Patients with a score of 9 or less are considered at very high risk. Two items of this score are specifically dedicated to assess surrogates of physical activity: Activity and Mobility with scores ranging from 1 to 4. We will evaluate the correlation between two items dedicated to mobility in the systematically documented Braden scale among internal medicine patients. In case of good reliability of either or both items, these variables which are already systematically documented by nurses could be integrated to VTE RAMs to identify patients warranting thromboprophylaxis.

Prognostic values on LOS, readmission, transfer to post-acute care facility of accelerometer-measured and nurses' estimation of mobility will be assessed.

**Figure 3. Braden Scale to predict the risk of pressure sores**

**BRADEN SCALE –Pressure Sore Risk**

| | | | | |
|---|---|---|---|---|
| **Sensory perception,** ***ability to respond to pressure related discomfort effectively*** | | | | |
| 1. Completely limited: Unresponsive | 2. Very limited: Responds only to painful stimuli. | 3. Slightly limited: Responds to verbal commands, cannot ask to turn | 4. No impairment: no sensory deficit limiting expression of discomfort | ***Score*** |
| **Moisture,** ***degree to which skin is exposed to moisture*** | | | | |
| 1.Constantly moist | 2.Very moist | 3.Occasionally moist | 4.Rarely moist | ***Score*** |
| **Activity,** ***degree of physical activity*** | | | | |
| 1. Bedfast | 2. Chair fast | 3. Walks occasionally | 4. Walks often | ***Score*** |
| **Mobility,** ***ability to change and Control Position*** | | | | |
| 1. Completely immobile | 2. Very limited | 3. slightly limited | 4. No limitation | ***Score*** |
| **Nutrition,** ***usual food intake pattern*** | | | | |
| 1. Very poor | 2. Probably inadequate | 3. Adequate | 4. Excellent | ***Score*** |
| **Friction and shear** | | | | |
| 1. Problem | 2. Potential problem | 3. No apparent problem | | ***Score*** |
| 15-18 Low Risk, 13-14 Moderate Risk, 10-12 High Risk, >9 Very High Risk | | | TOTAL | |

### 3.3.3.5 Substudy III: Feasibility of a future randomized controlled trial

In a subset of 100 patients the recruitment feasibility for a future randomized controlled trial comparing different VTE prevention strategies will be assessed. Patients who are at high risk of HA-VTE as identified by the simplified Geneva score and who are meeting the eligibility criteria for this hypothetical randomized trial will be approached at the discharge visit to evaluate acceptability to be enrolled in such a trial.

- Inclusion criteria:
    1) Hospitalized for acute non-surgical illness
    2) Expected duration of hospital stay ≥48 hours
    3) High VTE risk as assessed by the simplified Geneva score (≥3 points)

- Exclusion criteria
    1) Known hypersensitivity to heparin
    2) History of heparin induced thrombocytopenia
    3) Glomerular filtration rate (GFR) <15ml/min
    4) Platelet count <50,000G/L
    5) Liver failure with spontaneous INR >2.0
    6) Active bleeding
    7) Hemorrhagic transformation of acute ischemic stroke
    8) Requiring fibrinolysis/thrombolysis within 24h
    9) Any type of anticoagulation given for >48h before baseline inclusion
    10) Prior hospitalization for >72h
    11) Major surgery within last month, or planned during index hospitalization
    14) Pregnancy or breastfeeding

The information to assess the eligibility criteria will be collected at baseline.
At the discharge visit, participants who meet these potential trial eligibility criteria will receive oral information regarding a hypothetical randomized placebo-controlled trial comparing pharmacological VTE prophylaxis with enoxaparin 40mg s.c. once daily to placebo s.c. once daily with or without a mobility intervention for the duration of hospital stay until discharge, or for a maximum duration of 14 days. Patients randomized to the placebo group would subsequently be divided into standard care and increased mobility arms. Patient in the increased mobility arms will be asked to walk every day a certain amount of steps, or for those who are unable to walk, to engage in a mobility program which will be done in bed (e.g. three times daily and meals taken out of bed if possible).
Study nurses or investigators will answer questions from potential trial participants, who will then be asked whether they would be willing to provide consent for enrolment in such a trial. In

case they will refuse, the reason for refusal will be assessed as free text. If they would be willing to provide informed consent to participate in the study, patients will then be asked about their preferred follow-up schedule.
We chose the discharge visit to inquire about the potential willingness to participate in a hypothetical trial in order to prevent that TPX provision will be influenced by our study.

#### 3.3.4 Follow-up visit prior to discharge

A follow-up visit will be conducted the day prior to discharge or the day of discharge. Information on the discharge location (home, nursing home, rehabilitation, other hospital), and the date of discharge (in order to assess length of hospital stay) will be obtained from medical records and/or interview of the participant and treating health care personnel (**Table 5**). In addition, potential medical outcome events will be assessed (see section 2.2). Autonomy will be assessed in a face-to-face interview using the modified Barthel Index (see sections 2.2.3 and 3.3.3.1).

Similar to the baseline visit, information on treatments since admission with a focus on pharmacological and mechanical TPX will be assessed in order to determine duration of TPX (**Table 5**).

Study personnel will collect the accelerometer and upload accelerometry data to the database using the relevant software (Geneactiv and R).

In case a patient dies during the hospitalization, study nurses will collect the accelerometer on the day of death. Also, information on the circumstances of death from medical records and interview of the treating physicians will be collected.

**Table 5. Data collection at discharge**

| |
|---|
| **Discharge information**<br>Date of discharge, discharge location |
| **Medical outcome events**<br>HA-VTE, all-cause death, major bleeding, clinically relevant non-major bleeding |
| **Autonomy**<br>Modified Barthel Index |
| **Treatments since admission**<br>Pharmacological thromboprophylaxis: type (low molecular weight heparin, unfractionated heparin, fondaparinux, other), start date; mechanical VTE prophylaxis (lower extremity compression stockings/bandages, intermittent pneumatic compression devices); therapeutic anticoagulation, red blood cell transfusions (and date), central venous catheter, surgery |

#### 3.3.5 Follow-up phone call 90 days after enrolment

A final follow-up call will be scheduled at day 90 (range day 85-95) after study inclusion. The follow-up phone call will be conducted by trained study personnel at the local study sites. Medical outcomes (HA-VTE, all-cause mortality, major bleeding and clinically relevant non-major bleeding), subsequent hospitalizations since discharge, autonomy in the activities of daily living will be assessed. Assessment of endpoints is described in section 2.2.3. As initiation of therapeutic anticoagulation during follow-up will affect the outcomes, we will also collect information on new treatments, i.e. therapeutic anticoagulation since discharge.

### 3.4 Risks and measures to minimize the risks

Ideally, we would conduct this study in medical inpatients who do not receive any TPX, which is ethically not feasible. Thus, a substantial proportion of patients (<60%) will receive TPX,[18] which may decrease VTE risk and by consequence incidence of HA-VTE. However, as no RAM is formally implemented in the participating hospitals, we expect that patients with and without TPX will be well distributed in low and high VTE risk groups.[21] Also, we will adjust our analyses for

receipt of TPX and conduct sensitivity analyses excluding patients with TPX. Given the non-invasive nature of accelerometry and its good acceptability among inpatients based on the literature and on PI's practical experience,[34,64] we do not expect its implementation to cause any problems. Due to the observational design of our study, we do not expect safety issues for the patients enrolled.

### 3.5 Withdrawal and discontinuation

Participants can leave the study at any time for any reason if they wish to do so without any consequences. Investigators will make every reasonable effort to keep each patient on the study until all planned assessments have been performed. Protocol violations will not lead to patient withdrawal from the study.

Withdrawals were accounted for in our sample size calculation (see section 4.1.1); therefore, participants who withdraw from the study after provision of informed consent will not be replaced.

For a patient who withdraws consent for accelerometry-based measurements or for follow-up prior to hospital discharge, the option of an assessment at 90 days will be provided. Whether a withdrawing patient agrees or refuses to have the final 90 day assessment performed, this will be noted in the CRF.

If a study withdrawal occurs, coded data collected up to the time of withdrawal may nevertheless be used, as stipulated by the Swiss Human Research Ordinance (HRO Art. 10); we will inform potential participants in the patient information that anonymization is not possible, as done previously.[46] In addition, patients withdrawing consent for further study participation will be asked whether he/she would nevertheless agree with follow-up information being collected based on available hospital records and/or follow-up with his/her primary care physician, and the patients' decision will be noted in the CRF.

If a participant is lost to follow-up (i.e. a participant whose status is unclear because he/she cannot be reached for the follow-up phone call), the primary care physician and/or surrogate (relative or close friend) that were indicated by the participant during the baseline visit will be contacted and the hospital electronic records will be verified to obtain information about recurrent VTE and vital status. The steps taken to contact the participant, his/her primary care physician, and surrogates (i.e. dates of phone calls) will be noted in the CRF.

## 4 STATISTICS AND METHODOLOGY

### 4.1 Statistical analysis plan

#### 4.1.1 Sample size calculation

For work package 1, assuming that 67% of patients will be categorized as high risk and 33% as low risk based on the simplified Geneva score, and assuming that the 90-day incidence of HA-VTE is 2.8% in high risk and 0.6% in low risk patients with and without TPX,[18] we will need 1308 patients to detect an absolute risk difference of 2.2%, with a power of 80% at a 2-sided alpha of 0.05. The numbers stated above correspond to a relative risk of 4.7, a sensitivity of 90%, and a specificity of 34%.[18] This sample size will provide sufficient precision for the validation of the simplified Geneva score. Assuming an area under the curve (AUC) of 0.75, the normal-approximation 95% confidence interval (CI) will range from 0.64 to 0.86. For sensitivity and specificity, the 95% Wilson CIs will range from 72% to 96% and from 31% to 36%, respectively. We will recruit 1350 patients to account for potential dropouts, which will be few given the low follow-up burden.[14]

In work package 2, we will assess the same measures of association and prognostic accuracy as in work package 1, but using immobility measures rather than the RAMs, or a combination thereof. The sample size of 1350 patients will provide comparable precision as stated above.

#### 4.1.2 Statistical analyses

Work package 1) Time to event analyses with competing risk methods will be used to assess the prognostic performance of the simplified Geneva score and the other RAMs (**Table 1**) and their association with HA-VTE, with non-VTE death representing the competing risk. We will use a subdistribution hazard model of Fine and Gray to assess the association of the simplified Geneva score and the other RAMs with VTE, calculating subhazard ratios with 95% CIs.[65] These analyses will be adjusted for the use of TPX and study site. Cumulative incidences of HA-VTE in low- and high-risk score patients will be assessed and graphically presented to assess calibration and compare different RAMs. The time-dependent area under the curve (AUC) as well as sensitivity, specificity, and positive and negative predictive values will be calculated for each RAM to assess their accuracy to predict HA-VTE at 90 days using time-dependent receiver operating characteristic (ROC)-curve analysis, taking into account censored data and competing events.
Additionally, because some patients will be treated with TPX and thus have a lower risk of VTE, we will perform separate sensitivity analyses in patients with and without TPX. It is possible that a small number of patients will be started on therapeutic dose anticoagulation for reasons other than VTE (e.g. new onset atrial fibrillation) during follow-up; these patients will be censored in the main analysis.
Secondary time-to-event outcomes (major and clinically relevant non-major bleedings) will also be evaluated using competing risk regression. For all-cause mortality, we will use an ordinary Cox regression, for length of stay an accelerated failure time model. Binary outcomes (in-hospital VTE and readmission) will be evaluated using logistic regression. The Barthel-index will be assessed using linear regression. All models will be adjusted for the use of TPX and study site.

Work package 2) We will examine the association between subjective (physician's perception) and objective (accelerometry-measured) mobility levels, as a continuous measure as well as divided into quartiles, and 90-day cumulative incidences of HA-VTE using competing risk regression (accounting for non-VTE related death as a competing event), unadjusted as well as adjusted for TPX and study site. To define an optimal cutoff for objective immobility, we will assess sensitivity and specificity at different mobility levels using time-dependent ROC-curve analysis accounting for censored data and competing events. To compare the predictive performance of the simplified Geneva score using the standard subjectively-assessed definition of immobilization (i.e. physician perception) versus using objective accelerometry-assessed mobility measures, we will use likelihood ratio tests and/or the Akaike information criterion (AIC) as well as the c-statistics in competing risk models; immobility measures will be used as single covariates, as well as incorporated in the simplified Geneva score. We will assess the net reclassification index to assess improvement in risk prediction if using the accelerometry-based instead of the subjective immobility measure for the simplified Geneva score.

### 4.2 Handling of missing data

We anticipate only little missing data on either the primary outcome or on important baseline covariates (<5%). Competing risk regression analyses with censoring will be used to account for dropouts and patients that die.
Drop-outs will not be replaced, as we have accounted for drop-outs in the sample size calculation (see section 4.1.1).

## 5 5 REGULATORY ASPECTS AND SAFETY

### 5.1 Local regulations / Declaration of Helsinki

This research project will be conducted in accordance with the protocol, the Declaration of Helsinki [3], the principles of Good Clinical Practice, the Human Research Act (HRA) and the Human Research Ordinance (HRO) [1] as well as other locally relevant regulations. The Project Leaders acknowledge their responsibilities as the project leader, one of them also acting as the the Sponsor.

### 5.2 Notification of safety and protective measures (HRO Art. 20)

The project leaders are promptly notified (within 24 hours) if immediate safety and protective measures have to be taken during the conduct of the research project. The Ethics Committee will be notified via BASEC of these measures and of the circumstances necessitating them within 7 days.

### 5.3 5.3 Serious events (HRO Art. 21)

If a serious event occurs, the research project will be interrupted and the Ethics Committee notified on the circumstances via BASEC within 7 days according to HRO Art. 21.[1]

A serious event is defined as any adverse event where it cannot be excluded that the event is attributable to the collection of health-related personal data, and which:[1]
a) requires inpatient treatment not envisaged in the protocol or extends a current
hospital stay;
b) results in permanent or significant incapacity or disability; or
c) is life-threatening or results in death.

### 5.4 Amendments

Substantial changes to the project set-up, the protocol and relevant project documents will be submitted to the Ethics Committee for approval according to HRO Art. 18 before implementation. Exceptions are measures that have to be taken immediately in order to protect the participants.

### 5.5 End of project

Upon project termination, the Ethics Committee is notified within 90 days. All patient-level data obtained during the conduct of this project will be retained and archived for a period of at least 15 years from the completion or premature termination of the project in a coded manner. The Investigators should take measures to prevent accidental or premature destruction of these documents. Paper forms including written source data will be shredded after 10 years.

### 5.6 Insurance

The Insel Gruppe AG (through Zurich Versicherungs-Gesellschaft AG) provides insurance for the conduct of this study.

## 6 FURTHER ASPECTS

### 6.1 Overall ethical considerations

We will conduct this study in accordance with the Declaration of Helsinki, the ICH-GCP guidelines, and all applicable legal/regulatory requirements. Prior to initiation of the study, authorization by the local Ethics Committees shall be obtained.

Given the broad eligibility criteria, the results of this study will be generalizable to the population of hospitalized medical patients at risk of HA-VTE, i.e. those without intake of therapeutic anticoagulation.

Informed consent will be obtained from each participant prior to the conduct of any study related procedures. For participants who are unable to give informed consent due to mental illness or cognitive impairment, permission to participate in the study will be obtained from a legally authorized representative, as outlined in section 3.2. We will include patients with cognitive impairment/confusion as they are at increased risk of immobilization during hospital stay, with a consecutive increase in the risk of hospitalization-related adverse outcomes such as HA-VTE.[53,54] Excluding these patients would limit the generalizability of the results and question their applicability to this population, for whom the study findings might have the greatest relevance (see section 6.3).

A prospective cohort design is the optimal study design and provides the highest quality data to meet the aim of this study, i.e. to validate the simplified Geneva score, to compare

existing VTE risk assessment models, and to assess the prognostic value of objectively measured mobility concerning the risk of HA-VTE. A longitudinal study design is necessary to investigate prognostic measures, and prospective data collection allows to have complete and standardized measurements of exposures prior to the occurrence of any outcomes; also, objective mobility measurement is only possible in a prospective manner.

The majority of the baseline data collected for this study include variables that are routinely collected in clinical practice in hospitalized medical patients. The burden of mobility measurement using accelerometry is low for patients: the device is attached around the wrist and should be worn continuously (day and night). Prior studies have demonstrated good acceptability of this device among inpateints.[64]The burden of follow-up assessments will be minimized by performing a single follow-up phone call at 90 days after study inclusion, without the need for onsite visits during follow-up. No financial incentives will be offered.

Even though the receipt of TPX will be recorded for each participant, we will not influence the decision of the health care providers on the provision of TPX, as this is a purely observational study. Thus, we will not intervene on inadequate TPX provision, e.g. if TPX is provided to patients at low risk of HA-VTE, or if TPX is withheld in patients at high VTE risk. Participants will be informed accordingly in the patient information. We do not expect to detect any other incidental findings that may influence the health or treatment decisions in participants, as the vast majority of the data collected for this study is data that is routinely collected in clinical practice and therefore also available to the treating health care personnel.

Data collected in this project contains sensitive personal data. Confidentiality of all study participants will be maintained; all study forms with patient identifiers will be kept in locked file cabinets; all directly identifying information will not be shared but, if possible, recoded. For example, actual dates will be recoded as days since enrollment. Access to data collection forms and databases will be restricted to dedicated study personnel and database management personnel.

No conflict of interest exists with regard to the intellectual concern and proprietary affairs.

## 6.2 Risk-Benefit Assessment

This is an observational study, so no major health risk is expected. The follow-up burden will be very low, with one in-person visit at the bedside of the participant prior to discharge, and one follow-up phone call at 90 days after study inclusion.

A potential risk for project participants is unauthorized data access or unwanted identification of project participants. However, every reasonable measure will be taken to protect the confidentiality of participants. Codes for subject identifier will be utilized, and all directly identifying information will be recoded if possible (e.g. actual dates will be recoded as days since enrolment). Data entered into case report forms (CRFs) are transferred to the database using Secure Sockets Layer (SSL) encryption. Data will be stored in dedicated servers, which are located in dedicated, locked server rooms with restricted access. All servers are regularly backed-up using a multi-level system. Access to the eCRF and database is restricted to trained users and is logged. A role concept regulates permission for each user. All paper study forms with patient identifiers will be kept in locked file cabinets with controlled and restricted access.

For the individual participating patient, we cannot guarantee any benefits arising from study participation. It may be possible that potential complications of a hospital stay such as recurrent VTE events can be recognized more quickly given that two follow-up assessments are performed. The results of this study will provide a clear guidance for physicians about optimal VTE risk assessment. Thus, future medical inpatients may benefit from the results of this study due to optimized VTE risk assessment by their physicians, which has the potential to result in improved VTE prevention and a reduction in HA-VTE events in this population (see also section 1.2).

## 6.3 Rationale for the inclusion of vulnerable participants

For participants who are unable to give informed consent due to mental illness or cognitive impairment, permission to participate in the study will be obtained from a legally authorized representative. We will not exclude these patients, because the risk of VTE and immobilization and the associated adverse outcomes are particularly high in the elderly,[53,54] where cognitive impairment is more prevalent.

# 7 QUALITY CONTROL AND DATA PROTECTION

## 7.1 Quality measures

For quality assurance the Ethics Committee may visit the research sites. Direct access to the source data and all project related files and documents must be granted on such occasions.

We will implement following measures to ensure optimal data quality and completeness: (1) the study personnel involved in data collection will receive formal training in the methods of data abstraction, patient inquiry, and data recording; (2) baseline and follow-up data will be recorded on standardized eCRFs mostly containing already coded data fields e.g. via drop-down lists or radio buttons (number fields are preformatted and will contain range checks, and plausibility checks will be implemented between data fields); (3) an operations manual will be developed and distributed that includes definitions and acceptable data sources for all data variables.

Principal investigators of the recruiting sites will check the data entered by study personnel for completeness. The number and reasons for exclusion of excluded participants will be compared to assess the potential for selection bias.

## 7.2 Data recording and source data

We will record data in electronic CRFs using REDCap, an International Council on Harmonization Good Clinical Practice (ICH-GCP)-compliant database system. To establish, host, and maintain the database, we will receive support from the CTU Bern. Only authorized study and database management personnel will have access to the database, and any change to the data will be trackable through an audit trail.

All collected data will have a dedicated source. Data source documents will include (1) all original documents relating to the study, i.e., paper forms with baseline data collected directly from the patient in the project CRF; discharge letters, laboratory and radiology reports from routine daily practice; (2) data directly entered into the electronic case report forms (eCRF); and 3) output data from accelerometry ( .bin files). Study nurses will enter all patient baseline (following enrolment) and follow-up data (at the day prior to hospital discharge and after the follow-up interview) for each enrolled patient in a password-secured, web-based, standardized eCRF. The data source will be documented for all data captured in the eCRF.

All study personnel involved in data collection will receive formal training in the methods of data abstraction, patient inquiry, and data recording before they start entering data. An operations manual will be developed that includes definitions and acceptable data sources for all variables. Most data fields in the eCRF are already coded e.g. via drop-down lists or radio buttons.

## 7.3 Confidentiality and coding

Project data will be handled with uttermost discretion and is only accessible to authorized personnel who require the data to fulfil their duties within the scope of the research project. On the CRFs and other project specific documents, participants are only identified by a unique participant number.

To ensure data security and confidentiality, all data entered into the eCRF will be transferred to the database using Secure Sockets Layer (SSL) encryption. Data will be stored in dedicated servers located in locked server rooms with restricted access, and regular backups of all servers will be done using a multi-level system. Access to the password-secured eCRF and database is restricted to study and data management personnel and is logged. A role concept regulates permission for each user. All paper study forms with patient identifiers as well as the participant identification list will be kept in locked file cabinets with restricted access at the

corresponding recruiting study site. The database has an automatic audit trail to enable tracking of all changes of the data.

The participants' identifying contact details (see section 3.3.3.1) will be collected to contact the patients for the follow-up phone calls (or a surrogate in case the patient cannot be reached or has cognitive impairment) and to obtain information on outcomes. These identifying data will be securely stored at the local study site and not be entered in the research database.

### 7.4 Retention and destruction of study data and biological material

All patient-level data obtained during the conduct of this research will be retained and archived. Archiving of the trial database will last at least 15 years after the final analysis. Where possible, data will be stored in files using open formats (e.g. csv) along with proprietary formats (e.g. Stata). Paper forms (including written source data) will be shredded after 10 years. No biological materials will be collected for this project.

## 8 FUNDING / PUBLICATION / DECLARATION OF INTEREST

This study is funded by the Gottfried und Julia Bangerter-Rhyner Stiftung, the Novartis Foundation for medical-biological Research, the Chuard Foundation and the SGAIM Foundation.

The results of this project will be published in peer-reviewed journals that meet the open access policy from the Swiss National Science Foundation. Upon request of the journal, the protocol and the anonymized patient-level data and statistical code will be made available to the publishing journal.

With regard to the intellectual concern and proprietary affairs, no conflict of interest exists.

# 9 REFERENCES


1. Ordinance on Human Research with the Exception of Clinical trials (HRO). https://www.admin.ch/opc/en/classified-compilation/20121177/index.html
2. Human Research Act (HRA). http://www.admin.ch/opc/en/classified-compilation/20121176/201401010000/810.305.pdf
3. Declaration of Helsinki. https://www.wma.net/policies-post/wma-declaration-of-helsinki-ethical-principles-for-medical-research-involving-human-subjects.
4. Maynard G. Preventing hospital-associated venous thromboembolism: a guide for effective quality improvement. *2nd ed Rockville, MD: Agency for Healthcare Research and Quality, August 2016 AHRQ Publication No 16-0001-EF; accessed at: https://wwwahrqgov/sites/default/files/publications/files/vteguidepdf July 15, 2019.*
5. Falck-Ytter Y, Francis CW, Johanson NA, et al. Prevention of VTE in orthopedic surgery patients: Antithrombotic Therapy and Prevention of Thrombosis, 9th ed: American College of Chest Physicians Evidence-Based Clinical Practice Guidelines. *Chest.* 2012;141(2 Suppl):e278S-e325S.
6. Heit JA, Silverstein MD, Mohr DN, Petterson TM, O'Fallon WM, Melton LJ, 3rd. Risk factors for deep vein thrombosis and pulmonary embolism: a population-based case-control study. *Archives of internal medicine.* 2000;160(6):809-815.
7. Goldhaber SZ, Dunn K, MacDougall RC. New onset of venous thromboembolism among hospitalized patients at Brigham and Women's Hospital is caused more often by prophylaxis failure than by withholding treatment. *Chest.* 2000;118(6):1680-1684.
8. Alikhan R, Bedenis R, Cohen AT. Heparin for the prevention of venous thromboembolism in acutely ill medical patients (excluding stroke and myocardial infarction). *The Cochrane database of systematic reviews.* 2014(5):Cd003747.
9. Kakkar AK, Cimminiello C, Goldhaber SZ, Parakh R, Wang C, Bergmann JF. Low-molecular-weight heparin and mortality in acutely ill medical patients. *The New England journal of medicine.* 2011;365(26):2463-2472.
10. Samama MM, Cohen AT, Darmon JY, et al. A comparison of enoxaparin with placebo for the prevention of venous thromboembolism in acutely ill medical patients. Prophylaxis in Medical Patients with Enoxaparin Study Group. *The New England journal of medicine.* 1999;341(11):793-800.
11. Kahn SR, Lim W, Dunn AS, et al. Prevention of VTE in nonsurgical patients: Antithrombotic Therapy and Prevention of Thrombosis, 9th ed: American College of Chest Physicians Evidence-Based Clinical Practice Guidelines. *Chest.* 2012;141(2 Suppl):e195S-e226S.
12. Schunemann HJ, Cushman M, Burnett AE, et al. American Society of Hematology 2018 guidelines for management of venous thromboembolism: prophylaxis for hospitalized and nonhospitalized medical patients. *Blood advances.* 2018;2(22):3198-3225.
13. Nendaz M, Spirk D, Kucher N, et al. Multicentre validation of the Geneva Risk Score for hospitalised medical patients at risk of venous thromboembolism. Explicit ASsessment of Thromboembolic RIsk and Prophylaxis for Medical PATients in SwitzErland (ESTIMATE). *Thrombosis and haemostasis.* 2014;111(3):531-538.
14. Barbar S, Noventa F, Rossetto V, et al. A risk assessment model for the identification of hospitalized medical patients at risk for venous thromboembolism: the Padua Prediction Score. *Journal of thrombosis and haemostasis : JTH.* 2010;8(11):2450-2457.
15. Spyropoulos AC, Anderson FA, Jr., FitzGerald G, et al. Predictive and associative models to identify hospitalized medical patients at risk for VTE. *Chest.* 2011;140(3):706-714.
16. Stuck AK, Spirk D, Schaudt J, Kucher N. Risk assessment models for venous thromboembolism in acutely ill medical patients. A systematic review. *Thrombosis and haemostasis.* 2017;117(4):801-808.
17. Rosenberg D, Eichorn A, Alarcon M, McCullagh L, McGinn T, Spyropoulos AC. External validation of the risk assessment model of the International Medical Prevention Registry

on Venous Thromboembolism (IMPROVE) for medical patients in a tertiary health system. *Journal of the American Heart Association.* 2014;3(6):e001152.
18. Blondon M, Spirk D, Kucher N, et al. Comparative Performance of Clinical Risk Assessment Models for Hospital-Acquired Venous Thromboembolism in Medical Patients. *Thrombosis and haemostasis.* 2018;118(1):82-89.
19. Blondon M, Righini M, Nendaz M, et al. External validation of the simplified Geneva risk assessment model for hospital-associated venous thromboembolism in the Padua cohort. *Journal of thrombosis and haemostasis : JTH.* 2019.
20. Cohen AT, Tapson VF, Bergmann JF, et al. Venous thromboembolism risk and prophylaxis in the acute hospital care setting (ENDORSE study): a multinational cross-sectional study. *Lancet (London, England).* 2008;371(9610):387-394.
21. Spirk D, Nendaz M, Aujesky D, et al. Predictors of thromboprophylaxis in hospitalised medical patients. Explicit ASsessment of Thromboembolic RIsk and Prophylaxis for Medical PATients in SwitzErland (ESTIMATE). *Thrombosis and haemostasis.* 2015;113(5):1127-1134.
22. Theriault T, Touchette M, Goupil V, Echenberg D, Lanthier L. Thromboprophylaxis adherence to the ninth edition of American college of chest physicians antithrombotic guidelines in a tertiary care centre: a cross-sectional study. *Journal of evaluation in clinical practice.* 2016;22(6):952-957.
23. Pottier P, Hardouin JB, Lejeune S, Jolliet P, Gillet B, Planchon B. Immobilization and the risk of venous thromboembolism. A meta-analysis on epidemiological studies. *Thromb Res.* 2009;124(4):468-476.
24. Amin AN, Girard F, Samama MM. Does ambulation modify venous thromboembolism risk in acutely ill medical patients? *Thrombosis and haemostasis.* 2010;104(5):955-961.
25. Kahn SR, Panju A, Geerts W, et al. Multicenter evaluation of the use of venous thromboembolism prophylaxis in acutely ill medical patients in Canada. *Thromb Res.* 2007;119(2):145-155.
26. Hull RD. Relevance of immobility and importance of risk assessment management for medically ill patients. *Clinical and applied thrombosis/hemostasis : official journal of the International Academy of Clinical and Applied Thrombosis/Hemostasis.* 2013;19(3):268-276.
27. Spyropoulos AC, Ageno W, Albers GW, et al. Rivaroxaban for Thromboprophylaxis after Hospitalization for Medical Illness. *The New England journal of medicine.* 2018;379(12):1118-1127.
28. Brown CJ, Friedkin RJ, Inouye SK. Prevalence and outcomes of low mobility in hospitalized older patients. *Journal of the American Geriatrics Society.* 2004;52(8):1263-1270.
29. Callen BL, Mahoney JE, Grieves CB, Wells TJ, Enloe M. Frequency of hallway ambulation by hospitalized older adults on medical units of an academic hospital. *Geriatric nursing.* 2004;25(4):212-217.
30. Lazarus BA, Murphy JB, Coletta EM, McQuade WH, Culpepper L. The provision of physical activity to hospitalized elderly patients. *Archives of internal medicine.* 1991;151(12):2452-2456.
31. Hoyer EH, Young DL, Friedman LA, et al. Routine Inpatient Mobility Assessment and Hospital Discharge Planning. *JAMA internal medicine.* 2019;179(1):118-120.
32. Sinvani L, Kozikowski A, Patel V, et al. Measuring Functional Status in Hospitalized Older Adults Through Electronic Health Record Documentation. *Southern medical journal.* 2018;111(4):220-225.
33. Daskivich TJ, Houman J, Lopez M, et al. Association of Wearable Activity Monitors With Assessment of Daily Ambulation and Length of Stay Among Patients Undergoing Major Surgery. *JAMA network open.* 2019;2(2):e187673.
34. Lim SER, Ibrahim K, Sayer AA, Roberts HC. Assessment of Physical Activity of Hospitalised Older Adults: A Systematic Review. *The journal of nutrition, health & aging.* 2018;22(3):377-386.

35. Brown CJ, Roth DL, Allman RM. Validation of use of wireless monitors to measure levels of mobility during hospitalization. *Journal of rehabilitation research and development.* 2008;45(4):551-558.
36. Hartley P, Keevil VL, Westgate K, et al. Using Accelerometers to Measure Physical Activity in Older Patients Admitted to Hospital. *Current gerontology and geriatrics research.* 2018;2018:3280240.
37. Lim SER, Dodds R, Bacon D, Sayer AA, Roberts HC. Physical activity among hospitalised older people: insights from upper and lower limb accelerometry. *Aging clinical and experimental research.* 2018;30(11):1363-1369.
38. Rowlands AV, Olds TS, Hillsdon M, et al. Assessing sedentary behavior with the GENEActiv: introducing the sedentary sphere. *Medicine and science in sports and exercise.* 2014;46(6):1235-1247.
39. Brown CJ, Redden DT, Flood KL, Allman RM. The underrecognized epidemic of low mobility during hospitalization of older adults. *Journal of the American Geriatrics Society.* 2009;57(9):1660-1665.
40. Villumsen M, Jorgensen MG, Andreasen J, Rathleff MS, Molgaard CM. Very Low Levels of Physical Activity in Older Patients During Hospitalization at an Acute Geriatric Ward: A Prospective Cohort Study. *Journal of aging and physical activity.* 2015;23(4):542-549.
41. Tasheva P, Vollenweider P, Kraege V, et al. Association Between Physical Activity Levels in the Hospital Setting and Hospital-Acquired Functional Decline in Elderly Patients. *JAMA network open.* 2020;3(1):e1920185.
42. Hamilton AC, Lee N, Stilphen M, et al. Increasing Mobility via In-hospital Ambulation Protocol Delivered by Mobility Technicians: A Pilot Randomized Controlled Trial. *J Hosp Med.* 2019;14(5):272-277.
43. Kotaska A. Venous thromboembolism prophylaxis may cause more harm than benefit: an evidence-based analysis of Canadian and international guidelines. *Thrombosis journal.* 2018;16:25.
44. Office of the Surgeon G, National Heart L, Blood I. Publications and Reports of the Surgeon General. In: *The Surgeon General's Call to Action to Prevent Deep Vein Thrombosis and Pulmonary Embolism.* Rockville (MD): Office of the Surgeon General (US); 2008.
45. Buller HR, Cohen AT, Davidson B, et al. Idraparinux versus standard therapy for venous thromboembolic disease. *The New England journal of medicine.* 2007;357(11):1094-1104.
46. Mean M, Righini M, Jaeger K, et al. The Swiss cohort of elderly patients with venous thromboembolism (SWITCO65+): rationale and methodology. *Journal of thrombosis and thrombolysis.* 2013;36(4):475-483.
47. Cote LP, Greenberg S, Caprini JA, et al. Comparisons Between Upper and Lower Extremity Deep Vein Thrombosis: A Review of the RIETE Registry. *Clinical and applied thrombosis/hemostasis : official journal of the International Academy of Clinical and Applied Thrombosis/Hemostasis.* 2017;23(7):748-754.
48. Roy PM, Rachas A, Meyer G, et al. Multifaceted Intervention to Prevent Venous Thromboembolism in Patients Hospitalized for Acute Medical Illness: A Multicenter Cluster-Randomized Trial. *PloS one.* 2016;11(5):e0154832.
49. Schulman S, Kearon C, Subcommittee on Control of Anticoagulation of the S, Standardization Committee of the International Society on T, Haemostasis. Definition of major bleeding in clinical investigations of antihemostatic medicinal products in non-surgical patients. *Journal of thrombosis and haemostasis : JTH.* 2005;3(4):692-694.
50. Raskob GE, Spyropoulos AC, Zrubek J, et al. The MARINER trial of rivaroxaban after hospital discharge for medical patients at high risk of VTE. Design, rationale, and clinical implications. *Thrombosis and haemostasis.* 2016;115(6):1240-1248.
51. Shah S, Vanclay F, Cooper B. Improving the sensitivity of the Barthel Index for stroke rehabilitation. *Journal of clinical epidemiology.* 1989;42(8):703-709.
52. World Health Organization. Manual of the international statistical classification of diseases, injury, and causes of death. Geneva. 1977.

53. Alikhan R, Cohen AT, Combe S, et al. Risk factors for venous thromboembolism in hospitalized patients with acute medical illness: analysis of the MEDENOX Study. *Archives of internal medicine.* 2004;164(9):963-968.
54. Laporte S, Mismetti P, Decousus H, et al. Clinical predictors for fatal pulmonary embolism in 15,520 patients with venous thromboembolism: findings from the Registro Informatizado de la Enfermedad TromboEmbolica venosa (RIETE) Registry. *Circulation.* 2008;117(13):1711-1716.
55. Darzi AJ, Karam SG, Charide R, et al. Prognostic factors for VTE and Bleeding in Hospitalized Medical Patients: a systematic review and meta-analysis. *Blood.* 2020.
56. Charlson ME, Pompei P, Ales KL, MacKenzie CR. A new method of classifying prognostic comorbidity in longitudinal studies: development and validation. *Journal of chronic diseases.* 1987;40(5):373-383.
57. Esliger DW, Rowlands AV, Hurst TL, Catt M, Murray P, Eston RG. Validation of the GENEA Accelerometer. *Med Sci Sports Exerc.* 2011;43(6):1085-1093.
58. Dieu O, Mikulovic J, Fardy PS, Bui-Xuan G, Beghin L, Vanhelst J. Physical activity using wrist-worn accelerometers: comparison of dominant and non-dominant wrist. *Clin Physiol Funct Imaging.* 2017;37(5):525-529.
59. Rowlands AV, Yates T, Davies M, Khunti K, Edwardson CL. Raw accelerometer data analysis with GGIR R-package: does accelerometer brand matter? *Medicine and science in sports and exercise.* 2016;48(10):1935-1941.
60. Dillon CB, Fitzgerald AP, Kearney PM, et al. Number of days required to estimate habitual activity using wrist-worn GENEActiv accelerometer: A cross-sectional study. *PloS one.* 2016;11(5):e0109913.
61. Decousus H, Tapson VF, Bergmann JF, et al. Factors at admission associated with bleeding risk in medical patients: findings from the IMPROVE investigators. *Chest.* 2011;139(1):69-79.
62. Hostler DC, Marx ES, Moores LK, et al. Validation of the International Medical Prevention Registry on Venous Thromboembolism Bleeding Risk Score. *Chest.* 2016;149(2):372-379.
63. Bergstrom N, Braden B, Norvell K, Lenaghan P, Boynton P. Diminished tissue tolerance: influence on pressure sore development in the institutionalized elderly. *Applied nursing research : ANR.* 1988;1(2):96.
64. Tasheva P, Kraege V, Vollenweider P, Mean M, Marques-Vidal P. Level of physical activity in elderly patients hospitalized in a Swiss university hospital. Accessed at: https://primary-hospital-care.ch/fileadmin/content/Supplements/PHC-Suppl_10.pdf, September 16, 2019.
65. Fine JP, Gray RJ. A Proportional Hazards Model for the Subdistribution of a Competing Risk. *Journal of the American Statistical Association.* 1999;94(446):496-509.

## Synopsis of Study Protocol

| | |
|---|---|
| **Sponsor / Sponsor-Investigator** | **Inselspital, Bern University Hospital:**<br>Dr. med. Christine Baumgartner, MAS<br>Department of General Internal Medicine<br>Inselspital, Bern University Hospital<br>University of Bern<br>Freiburgstrasse<br>3010 Bern<br>Switzerland<br>Phone: +41 (0)31 632 57 69<br>e-mail: Christine.Baumgartner@insel.ch<br><br>**Co-Principal Investigator:**<br>Dr. med. Marie Méan<br>Service de Médecine Interne<br>Centre Hospitalier Universitaire Vaudois (CHUV)<br>Rue du Bugnon 46<br>1011 Lausanne<br>Switzerland<br>Phone: +41 (0)21 314 11 11<br>e-mail: Marie.Mean@chuv.ch |
| **Study Title:** | Risk Stratification for Hospital-Acquired Venous Thromboembolism in Medical Patients: a Prospective Cohort Study |
| **Short Title / Study ID:** | RISE Study |
| **Protocol Version and Date:** | Version 1.0 / March 17, 2020 |
| **Trial registration:** | N/A |
| **Study category and Rationale** | This study is a risk category A study because it is an observational non-interventional study and the measures to collect personal data only entail minimal risks and burden. |
| **Clinical Phase:** | N/A |
| **Background and Rationale:** | Hospital-acquired venous thromboembolism (HA-VTE) is one of the leading preventable causes of in-hospital mortality, but prevention of VTE in hospitalized medical patients remains challenging, as preventive measures such as pharmacological thromboprophylaxis (TPX) need to be tailored to individual thrombotic risk. Currently, VTE risk stratification in medical patients is done using risk assessment models (RAMs) incorporating an array of demographic and clinical patient characteristics. Given the practical limitations of current RAMs, a new, easier to use RAM has been recently derived in Switzerland, which needs prospective external validation before it can be widely recommended for use in clinical practice. Immobilization, which is a strong risk factor for VTE, is therefore considered as a predictor in existing RAMs and beyond. Because patient's immobilization relies most often on physician's estimation and because no standardized definition for immobilization exists, the practical usefulness of this predictor is limited. Thus, whether novel, objective measures of immobilization, such as accelerometry-assessed patient's mobility, could predict HA-VTE must be examined. |

| | |
|---|---|
| **Objective(s):** | The **broad objective** of this study is to improve VTE prevention strategies in hospitalized medical patients by prospectively validating a novel Swiss RAM, the simplified Geneva score, and by developing a new, objective, and innovative definition of patient immobilization in the hospital using accelerometry.<br><br>The specific objectives of this project are divided into two work packages (WPs):<br>**WP 1: <u>Evaluate current VTE risk prediction strategies</u>**<br>**1)** to prospectively validate the simplified Geneva score as a RAM to predict HA-VTE (primary objective)<br>**2)** to compare the prognostic performance of the simplified Geneva score to discriminate between low and high VTE risk patients with previously validated RAMs (original Geneva, Padua, and IMPROVE score)<br>**WP 2: <u>Assess novel VTE risk prediction strategies using objective measures of mobility</u>**<br>**1)** to assess whether objective accelerometry-assessed immobilization predicts the risk of HA-VTE and to compare it to the standard subjective method to assess immobilization (physician perception)<br>**2)** to compare the predictive performance of the simplified Geneva score using the standard subjectively-assessed definition of immobilization (i.e. physician perception) versus using objective accelerometry-assessed mobility measures |
| **Outcome(s):** | The primary outcome will be symptomatic, objectively confirmed fatal and non-fatal HA-VTE up to 90 days after hospital admission.<br>The secondary outcomes will be symptomatic HA-VTE during hospitalization, all-cause mortality, major bleeding, and clinically relevant non-major bleeding up to 90 days after hospital admission, patient autonomy in the activities of daily living at hospital discharge and 90 days, length of hospital stay, and subsequent hospitalization up to 90 days. |
| **Study design:** | multicenter non-interventional prospective cohort study |
| **Inclusion / Exclusion criteria:** | Overall, 1350 consecutive adult patients hospitalized for acute illness in general internal medicine will be invited to participate in this study upon admission if they meet the eligibility criteria:<br>- **Inclusion criteria:**<br>1) age ≥18 years<br>2) admitted for hospitalization >24 hours on a general internal medicine ward<br>3) informed consent as documented by signature<br><br>- **Exclusion criteria:**<br>1) need for therapeutic anticoagulation (e.g., atrial fibrillation)<br>2) life expectancy <30 days<br>3) insufficient proficiency of the German or French language<br>4) unwilling to provide informed consent<br>5) prior enrolment in the study<br><br>Patients who are unable to give informed consent due to mental illness or cognitive impairment will not be excluded from participation, because the risk of VTE and immobilization and the associated adverse outcomes are particularly high in the elderly, where cognitive impairment is more prevalent. |
| **Measurements and procedures:** | At the time of hospital admission (baseline), following data will be collected: |

| | |
|---|---|
| | a) demographic data;<br>b) all items of the simplified and original Geneva score, IMPROVE score, and Padua score, and other potential VTE risk factors<br>c) comorbidities<br>d) potential contraindications to pharmacological TPX<br>e) laboratory findings that are relevant concerning VTE and bleeding risk<br>f) medications at admission with a potential antithrombotic effect<br>g) treatments during the current hospital stay<br>h) information on patient's autonomy (modified Barthel Index)<br>i) patient's ability to walk<br>j) risk of pressure sores (Braden scale)<br>Data will be collected from electronic health records, except for information such as contact details, mobility, paresis/paralysis, recent travel, dehydration, leg edema, patient autonomy, and ability to walk, which will be collected at the patient bedside (from the patient and/or nurse in charge) by trained study nurses.<br>In addition, objective mobility data will be measured continuously for the duration of hospital stay using a wrist-worn tri-axis accelerometer (GENEActiv Original, ActivInsights Ltd, UK).<br>On the second day of admission, study nurses will ask the treating physicians to judge whether their patients fulfill the different immobilization criteria, and to subjectively estimate the patient's mobility level in standardized terms.<br>Prior to discharge (1 day prior or day of discharge) the discharge location, length of stay, medical outcome events (HA-VTE, all-cause death, major bleeding, clinically relevant non-major bleeding), and treatments since admission will be obtained from medical records and/or interview of the participant and treating health care personnel. Autonomy will be assessed using the modified Barthel Index.<br>To assess outcome events, participants will be followed with a follow-up phone call 90 days after enrolment.<br><br>**<u>Substudies</u>**<br><u>Prospective validation of the Improve bleeding risk score</u><br>The IMPROVE bleeding risk score consisting of 11 demographical, clinical, and laboratory items was derived and validated to predict in-hospital bleeding in medical inpatients upon admission. All variable for the score will be collected at baseline. We aim to prospectively validate the IMPROVE bleeding risk score in this Swiss cohort of hospitalized medical patients.<br><u>Correlation between nurses' estimation of mobility and an objective motion measurement</u><br>The Braden scale has been validated to identify hospitalized patients at risk of pressure sores. This scoring system includes 6 items with a total score ranging from 0 to 23. Two items of this score are specifically dedicated to assess surrogates of physical activity: Activity and Mobility with scores ranging from 1 to 4. We will evaluate the correlation between two items dedicated to mobility in the systematically documented Braden scale among internal medicine patients. In case of good reliability of either or both items, these variables which are already systematically documented by nurses could be integrated to VTE RAMs to identify patients warranting thromboprophylaxis.<br><u>Feasibility of a future randomized controlled trial</u><br>In a subset of 100 patients the recruitment feasibility for a future randomized controlled trial comparing different VTE prevention strategies will be assessed. Patients who are at high risk of HA-VTE as identified |

| | |
|---|---|
| | by the simplified Geneva score and who are meeting the eligibility criteria for this hypothetical randomized trial will be approached prior to discharge and asked whether they would potentially accept to be enrolled in such a trial. |
| **Study Product / Intervention:** | N/A |
| **Control Intervention (if applicable):** | N/A |
| **Number of Participants with Rationale:** | Overall, we will recruit 1350 participants for this study.<br>The sample size calculation is based on WP 1, assuming that 67% of patients will be categorized as high risk and 33% as low risk based on the simplified Geneva score, and assuming that the 90-day incidence of HA-VTE is 2.8% in high risk and 0.6% in low risk patients with and without TPX, we will need 1308 patients to detect an absolute risk difference of 2.2%, with a power of 80% at a 2-sided alpha of 0.05. This corresponds to a relative risk of 4.7, a sensitivity of 90%, and a specificity of 34%. We will recruit 1350 patients to account for potential dropouts, which will be few given the low follow-up burden. For work package 2, we will use the same sample size. |
| **Study Duration:** | Overall study duration: 18 months (from first patient in to last patient out) |
| **Study Schedule:** | First-Participant-In (planned): May 1, 2020<br>Last-Participant-Out (planned): October 31, 2021 |
| **Investigator(s):** | **Inselspital, Bern University Hospital:**<br>Dr. med. Christine Baumgartner, MAS<br>Department of General Internal Medicine<br>Inselspital, Bern University Hospital<br>University of Bern<br>Freiburgstrasse<br>3010 Bern<br>Switzerland<br>Phone: +41 (0)31 632 57 69<br>e-mail: Christine.Baumgartner@insel.ch<br><br>**Centre Hospitalier Universitaire Vaudois (CHUV):**<br>Dr. med. Marie Méan<br>Service de Médecine Interne<br>Centre Hospitalier Universitaire Vaudois<br>Rue du Bugnon 46<br>1011 Lausanne<br>Switzerland<br>Phone: +41 (0)21 314 11 11<br>e-mail: Marie.Mean@chuv.ch<br><br>**Geneva University Hospital (HUG):**<br>Dr. med. Pauline Darbellay Farhoumand<br>Service de Médecine Interne Générale<br>Hôpitaux Universitaires de Genève<br>Rue Gebrielle-Perret –Gentil 4<br>1205 Geneva<br>Switzerland<br>Phone : +41 (0)22 372 92 03<br>e-mail: Pauline.Darbellay@hcuge.ch |
| **Study Centre(s):** | This is a multicenter study conducted in at least 2 high-volume hospitals in Switzerland |

| | |
|---|---|
| **Statistical Considerations:** | The association and performance of the simplified Geneva score, the 3 other RAMs, patient's immobilization assessed either by physician's estimation or by objective accelerometry-based measure for HA-VTE will be examined using time-to-event analyses with competing risk methods, adjusting for TPX use. Time-dependent receiver operating characteristic (ROC)-curves will be used to assess the discrimination and accuracy of the RAMs and mobility measures for HA-VTE. |
| **GCP Statement:** | This study will be conducted in compliance with the protocol, the current version of the Declaration of Helsinki, the ICH-GCP as well as all national legal and regulatory requirements. |

**Explanation for the Inclusion of vulnerable Subjects:**

For participants who are unable to give informed consent due to mental illness or cognitive impairment, permission to participate in the study will be obtained from a legally authorized representative. We will not exclude these patients, because the risk of VTE and immobilization and the associated adverse outcomes are particularly high in the elderly, where cognitive impairment is more prevalent.

**Recruitment Procedure:**

Consecutive hospitalized medical patients will be screened for recruitment at the General Internal Medicine wards of the participating sites. Study nurses will screen newly admitted patients (n≈12-15 per hospital/day) on a daily basis for eligibility.

**Study Procedure/Flowchart with Timelines:**

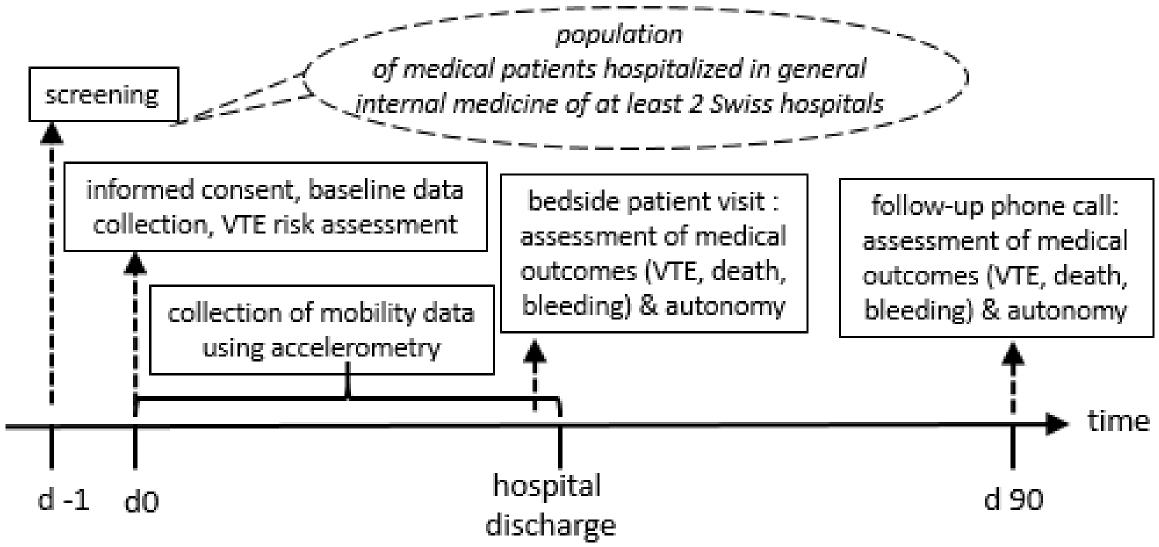


**Risks/ Inconveniences, which are Study specific:**

There are only minimal risks associated with participation in this project. A potential risk for project participants is unauthorized data access or unwanted identification of project participants.

**Coverage of Damages:**

The sponsor and Insel Gruppe AG have taken out insurance by the Zürich Versicherungs-Gesellschaft AG

**Storage of Data-and Samples for Future Research Aims:**
No.

**Ethical Considerations:**

1. Please describe the potential gain of new knowledge obtained with this study, and its meaning for patients/society.

Our project will provide the first prospective head-to-head comparison of RAMs and it will develop a novel concept and definition of immobilization as a predictor of HA-VTE in medical inpatients using accelerometry-assessed measures of mobility. If successful, our project has the potential to improve quality of care by optimizing VTE risk stratification and improving VTE prevention.

2. Please give an assessment of the benefit/risk relationship for the patient.

For the individual participating patient, we cannot guarantee any benefits arising from study participation. However, the risks associated with study participation are only minimal (i.e. breach of confidentiality).

3. Please explain, why the methodology is also ethically appropriate to gain new generalizable knowledge (for ex. double-blind, placebo, sham, vulnerable subjects, emergency cases, partial information only etc.)

To optimize generalizability of our results, we will not exclude patients who are unable to give informed consent due to mental illness or cognitive impairment, because the risk of VTE and immobilization and the associated adverse outcomes are particularly high in the elderly, where cognitive impairment is more prevalent.

**The most relevant References:**

Schunemann HJ, et al. American Society of Hematology 2018 guidelines for management of venous thromboembolism: prophylaxis for hospitalized and nonhospitalized medical patients. *Blood Adv.* 2018;2:3198-3225.

Stuck AK, Spirk D, Schaudt J, Kucher N. Risk assessment models for venous thromboembolism in acutely ill medical patients. A systematic review. *Thromb Haemost.* 2017;117(4):801-808.

Blondon M, Spirk D, Kucher N, et al. Comparative Performance of Clinical Risk Assessment Models for Hospital-Acquired Venous Thromboembolism in Medical Patients. *Thromb Haemost.* 2018;118(1):82-89.

Pottier P, et al. Immobilization and the risk of venous thromboembolism. A meta-analysis on epidemiological studies. *Thromb Res.* 2009;124:468-476.

Hoyer EH, et al. Routine Inpatient Mobility Assessment and Hospital Discharge Planning. *JAMA Intern Med.* 2019;179:118-120.

Lim SER, et al. Assessment of Physical Activity of Hospitalised Older Adults: A Systematic Review. *J Nutr Health Aging.* 2018;22:377-386.
