# Supplementary material for: Risk stratification for hospital-acquired venous thromboembolism in medical patients (RISE): Protocol for a prospective cohort study
Source: PLoS One. 2022 May 24;17(5):e0268833. doi: 10.1371/journal.pone.0268833 (PMC9128957; doi:10.1371/journal.pone.0268833)
Supplement: S2 File — (PDF) [file pone.0268833.s002.pdf]

Kanton Bern
Canton de Berne

Gesundheits-, Sozial- und Integrationsdirektion
Kantonale Ethikkommission für die Forschung

Murtenstrasse 31
3010 Bern
Bern
+41 31 633 70 70 (Telefon)
+41 31 633 70 71 (Telefax)
info.kek.kapa@be.ch
www.be.ch/gsi

Dorothy Pfiffner
+41 31 633 70 77
dorothy.pfiffner@be.ch

GSI-KEK, Murtenstrasse 31, 3010 Bern

Frau
Dr. med. Christine Baumgartner
Universitätsklinik für Allgemeine Innere Medizin
Inselspital Bern
Freiburgstrasse 18
3010 Bern

Bern, 28.04.2020, MF

Verfügung der KEK Bern, Sitzung vom 31.03.2020, Erstentscheid

**Project-ID**	2020-00606
**Projekttitel**	RISK STRATIFICATION FOR HOSPITAL-ACQUIRED VENOUS THROMBOEMBOLISM IN MEDICAL PATIENTS: A PROSPECTIVE COHORT STUDY
**Projektleitung**	Dr. med. Christine Baumgartner
**Sponsor**	Dr. med. Christine Baumgartner
**Leit-Ethikkommission**	**Zentren**
Kantonale Ethikkommission Bern	• Dr. med. Christine Baumgartner, Universitätsklinik für Allgemeine Innere Medizin, Inselspital, Bern
**Beteiligte Ethikkommissionen**	**Zentren**
VD	• Dr. Marie Méan, Centre hospitalier universitaire vaudois (CHUV), Lausanne

Entscheid

Dr. med. Christine Baumgartner, Inselspital, Bern University Hospital, Bern
Dr. Marie Méan, Centre hospitalier universitaire vaudois (CHUV), Lausanne

☐ Die Bewilligung wird erteilt

☒ Die Bewilligung wird mit Auflagen erteilt

☐ Die Bewilligung kann noch nicht erteilt werden

☐ Die Bewilligung wird nicht erteilt

☐ Auf das Gesuch wird nicht eingetreten

Die Dokumente sind gemäss den unten stehenden Punkten zu revidieren. Die Änderungen in den einzelnen Dokumenten müssen im Korrekturmodus abgefasst werden, sodass sowohl die neuen wie auch die alten Informationen sichtbar sind. Beim Hochladen der revidierten Unterlagen (jeweils 1x mit Korrekturmodus, 1x clean-Version) via Basec sind die alten Dokumente, die durch die neuen Dokumente ersetzt werden, zu löschen.

Allgemeine Auflagen:

1. Studieninformationen (Patient und gesetzlicher Vertreter):
   a. S.1: Präzisieren, dass die Studie vom Inselspital (Sponsor) organisiert wird und am Inselspital und am CHUV durchgeführt wird.

b. Kap. 4: Laienverständlich beschreiben, was ein Akzelerometer ist. Dieser wird in diesem Kapitel als Beschleunigungsmesser angegeben. Andernorts ist in den Studieninformationen von Schrittzähler die Rede.

2. Patienteninformation:
   Z.9/10: Bitte den folgenden Passus korrigieren: «*Wir arbeiten in der Allgemeininteressieren uns für die Verbesserung der Behandlungsqualität auf der Inneren Medizin.*»

LeitEK BE: Dr. med. Christine Baumgartner, Universitätsklinik für Allgemeine Innere Medizin, Inselspital, Bern

Die lokalen Gegebenheiten sind erfüllt.

Beteiligte EK VD: Dr. Marie Méan, Centre hospitalier universitaire vaudois (CHUV), Lausanne

Charge:

1. Contrat:
   Merci de nous faire parvenir le contrat, signé par toutes les parties, avant inclusion du premier patient.

Entscheid basierend auf der Beurteilung durch die Commission cantonale d'Éthique de la Recherche sur l'être humain Vaud (CER-VD).

Klassifizierung

☒ Forschungsprojekt gemäss HFV, Kategorie: A

- ☒ Forschung mit Personen
- ☐ Weiterverwendung des biologischen Materials oder der gesundheitsbezogenen Personendaten
- ☐ mit Verstorbenen
- ☐ mit Embryonen / Föten
- ☐ mit ionisierender Strahlung

Entscheidverfahren

☐ ordentliches Verfahren ☒ vereinfachtes Verfahren ☐ Präsidialverfahren

Am Entscheid beteiligte Kommissionsmitglieder siehe Anhang.

Die Ethikkommission bestätigt, dass sie nach ICH-GCP arbeitet.

Gebühren

Betrag: CHF 1500.— **Tarifcode:** 4.2.1

Gemäss der geltenden Gebührenordnung von swissethics. Rechnungsstellung folgt durch die Kantonale Gesundheits-, Sozial- und Integrationsdirektion (GSI).

Rechtsmittelbelehrung

Gegen diese Verfügung kann innert 30 Tagen seit Eröffnung bei der Gesundheits-, Sozial- und Integrationsdirektion des Kantons Bern Beschwerde erhoben werden. Die Beschwerdefrist kann nicht verlängert werden. Die Beschwerdeschrift ist im Doppel bei der Gesundheits-, Sozial- und Integrationsdirektion, Rathausgasse 1, 3011 Bern einzureichen.

Sie muss

(a) angeben, welche Entscheidung anstelle der angefochtenen Verfügung beantragt wird und
(b) aus welchen Gründen diese andere Entscheidung verlangt wird sowie
(c) die Unterschrift der beschwerdeführenden Partei oder der sie vertretenden Person enthalten.

Der Beschwerdeschrift beizulegen sind die Beweismittel, soweit sie greifbar sind, und die angefochtene Verfügung. (Art. 32 und 60 ff. des Gesetzes vom 23. Mai 1989 über die Verwaltungsrechtspflege [VRPG; BSG 155.21]).

Stand Dezember 2015

Kopie an

☐ BAG

☒ DLF

☒ Beteiligte Ethikkommissionen: Commission cantonale d'Éthique de la Recherche sur l'être humain Vaud (CER-VD)

Prof. Dr. med. Christian Seiler<br>Präsident KEK Bern	Dr. sc. nat. Dorothy Pfiffner<br>Vizepräsidentin<br>Leiterin Wissenschaftliches Sekretariat

Anhang: -Pflichten des Sponsors/der Prüfperson oder der Projektleitung
-Mögliche Entscheide und ihre Bedeutung
-Eingereichte Dokumente 20.03.2020
-Am Entscheid beteiligte Kommissionsmitglieder

Anhang

Pflichten des Sponsors/der Prüfperson oder der Projektleitung:

Einreichung Dokumente: revidierte Dokumente und neue Dokumente zur Studie/zum Projekt sollen ausschliesslich über das Web-Portal BASEC eingereicht werden, auf der entsprechenden Formularseite des betreffenden Gesuches. Obsolete Dokumente sind dabei zu entfernen und Datums- und Versionsangaben entsprechend zu ergänzen. Die erfolgten Änderungen müssen im Korrekturmodus abgefasst werden und zusätzlich als ‚clean'-Version eingereicht werden. Die Studieninformationen und -einwilligungen, das Protokoll und die Amendments müssen in durchsuchbaren PDF-Dateien eingereicht werden, insbesondere müssen gescannte Dokumente eine Texterkennung durchlaufen haben (OCR). Das unterschriebene und datierte Begleitschreiben muss die Antworten auf eventuell von der EK gestellte Fragen enthalten. Revidierte Dokumente sind auch den weiteren Zulassungsbehörden zuzustellen, sofern diese involviert sind.

Anmerkung: Die zuständige Ethikkommission überprüft im Rahmen des Bewilligungsverfahrens Aufklärungsbogen und Einwilligungserklärung in einer der Amtssprachen Deutsch, Französisch oder Italienisch. Aufklärungsbogen und Einwilligungserklärung in einer anderen Sprache werden von der Ethikkommission lediglich zur Kenntnis genommen. Für die korrekte Übersetzung ist der Sponsor oder die Projektleitung verantwortlich.

Meldepflichten: Die rechtlich bindenden Melde- resp. Bewilligungspflichten an die Ethikkommission für wesentliche Änderungen, einen vorzeitigen Studienabbruch, unerwünschte Ereignisse u.a. sind einzuhalten (Verordnungen des Bundes). Der Abschlussbericht ist spätesten ein Jahr nach Studienende der Ethikkommission einzureichen.

Registrierungspflicht: Der Sponsor muss – falls es sich um einen klinischen Versuch handelt – diesen in einem WHO-Primärregister oder im Register der Nationalen Medizinbibliothek der USA (clinicaltrials.gov) erfassen und anschliessend diese Nummer im BASEC-Portal eingeben. Die Übertragung der erforderlichen Daten in das Swiss National Clinical Trials Portal (SNCTP) kann nach Bewilligung der Ethikkommission und Zustimmung des Gesuchstellers automatisch erfolgen. Die Informationen über den klinischen Versuch sind in beiden Registern öffentlich zugänglich. Zusätzlich veröffentlicht swissethics wenige Informationen wie Titel, Projekttyp oder Leit-Ethikkommission aller durch die kantonalen Ethikkommissionen bewilligten Gesuche auf swissethics.ch (ausser Phase-I-Studien).

Mögliche Entscheide und ihre Bedeutung

Die Bewilligung wird erteilt: Das Vorhaben kann gemäss bewilligtem Forschungsplan und im Rahmen der anwendbaren rechtlichen Bestimmungen durchgeführt werden. Weitere Bewilligungspflichten (Swissmedic/BAG) sind zu beachten

Die Bewilligung wird mit Auflagen erteilt: Das Vorhaben kann gemäss bewilligtem Forschungsplan gestartet werden und im Rahmen der anwendbaren rechtlichen Bestimmungen durchgeführt werden. Die Auflagen sind zu erfüllen und die Gesuchsunterlagen innert 30 Tagen entsprechend anzupassen. Die revidierten Dokumente werden nach Einreichung im Präsidialverfahren geprüft. Weitere Bewilligungspflichten (Swissmedic/BAG) sind zu beachten

Die Bewilligung kann noch nicht erteilt werden: Das Vorhaben kann noch nicht gestartet werden. Die nachfolgenden Bedingungen sind zu erfüllen bzw. die Fragen zu beantworten und die revidierten Dokumente erneut bei der Ethikkommission einzureichen. Die Ethikkommission überprüft die revidierten Dokumente und erteilt die Bewilligung, wenn die Bedingungen erfüllt bzw. die Fragen zufriedenstellend beantwortet sind.

Die Bewilligung wird nicht erteilt: Das Vorhaben kann in der vorliegenden Form nicht durchgeführt werden. Eine Neueinreichung ist möglich.

Auf das Gesuch wird nicht eingetreten: Begründung siehe vorne, z.B. nicht zuständig oder nicht bewilligungspflichtig.

Eingereichte Dokumente für das Hauptzentrum

Dr. med. Christine Baumgartner, Universitätsklinik für Allgemeine Innere Medizin, Inselspital, Bern

Dokument	Dok.Datum	Version
**1. Cover Letter**		
rise-study-cover-letter-signed.pdf	20/03/2020	
**2. Synopsis of the study plan**		
rise-study-synopsis-v1-0.pdf	17/03/2020	v1.0
**3. Participant information sheet and informed consent (ICF)**		
rise-study-patient-information-and-ic-d-v1-0-bern.docx	17/03/2020	v1.0
rise-study-patient-information-and-ic-f-v1-0-bern.docx	17/03/2020	v1.0
rise-study-representative-information-and-ic-d-v1-0-bern.docx	17/03/2020	v1.0
rise-study-representative-information-and-ic-f-v1-0-bern.docx	17/03/2020	v1.0
**4. Study plan (protocol), signed and dated**		
rise-study-protocol-v1-0-signed.pdf	17/03/2020	v1.0
**5. CRF (Case Report Form)**		
rise-study-baseline-crf-20200317.docx	17/03/2020	draft
rise-study-discharge-fup-cfr-20200317.docx	17/03/2020	draft
rise-study-90d-fup-crf-20200317.docx	17/03/2020	draft
**6. Investigator's CV, dated**		
cv-christine-baumgartner-20200317.pdf	17/03/2020	
cv-marie-mean-20200317.pdf	17/03/2020	
**9. Agreement between sponsor/commissioned institution / grant provider or other third parties and the investigator**		
1253-vte-ram-immobilization-clinicalresearchagreement-signed-20200313.pdf	26/11/2019	
rise-study-clinical-study-agreement-draft.docx	17/03/2020	
novartis-foundation-agreement.pdf	22/11/2019	
bangerter-agreement.pdf	17/02/2020	
**10. Insurance**		
police-probandenversicherung-zuerich-versicherung-01.pdf	30/10/2018	
**11. Other documents handed over to study participants**		
No other documents handed over to study participants		
**12. Details on nature and scope/value of compensation for participants**		
There is no compensation for the participation in this study		
**14. Information on secure handling of biological material and personal data, and in particular on the storage thereof**		
see doc/cat: **4**, page/ref: **Protocol page 26-28**		

Eingereichte Dokumente für Prüfzentren im Verantwortungsbereich der EK Vaud

Dr. Marie Méan, Centre hospitalier universitaire vaudois (CHUV), Lausanne

Dokument	Dok.Datum	Version
**2. Synopsis of the study plan**		
rise-study-synopsis-v1-0.pdf	17/03/2020	v1.0
**3. Participant information sheet and informed consent (ICF)**		
rise-study-patient-information-and-ic-d-v1-0-lausanne.docx	17/03/2020	v1.0
rise-study-patient-information-and-ic-f-v1-0-lausanne.docx	17/03/2020	v1.0
rise-study-representative-information-and-ic-f-v1-0-lausanne.docx	17/03/2020	v1.0
rise-study-representative-information-and-ic-d-v1-0-lausanne.docx	17/03/2020	v1.0
**4. Signed study plan or signature page of the study plan**		
rise-study-protocol-v1-0-signed.pdf	17/03/2020	v1.0
**6. Local Investigator's / Project Leader's CV, dated2**		
cv-marie-mean-20200317.pdf	17/03/2020	
**9. Agreement between sponsor/commissioned institution and local investigator**		
rise-study-clinical-study-agreement-draft.docx	05/02/2020	
**11. Other documents handed over to study participants**		
The information / document(s) provided for the main study site also cover this site.		

Zusammensetzung der am Entscheid beteiligten Kommission

					am Beschluss beteiligt		
					ja	nein	
	**Name, Titel**	**Berufliche Stellung/Titel**	**m**	**f**		**abwesend**	**In Ausstand**
	Prof. Dr. med. Dr. sc. nat. Chris Boesch	Abteilungsleiter,<br>Abt. Magentresonanz-Spektroskopie und Methodologie	X		X		
**Vizepräsident**	Prof. Dr. med. Robert Greif	Senior Consultant	X		X		
**Vizepräsidentin**	Dr. sc. nat. Dorothy Pfiffner	Leiterin KEK-Sekretariat		X	X		
**Präsident**	Prof. Dr. med. Christian Seiler	Stv. Chefarzt	X		X		
