# Supplementary material for: Risk stratification for hospital-acquired venous thromboembolism in medical patients (RISE): Protocol for a prospective cohort study
Source: PLoS One. 2022 May 24;17(5):e0268833. doi: 10.1371/journal.pone.0268833 (PMC9128957; doi:10.1371/journal.pone.0268833)
Supplement: S3 File — (PDF) [file pone.0268833.s003.pdf]

Studieninformation: RISE Studie

Untersuchung des Risikos für Spital-assoziierte venöse Thromboembolien: eine prospektive Kohortenstudie

Originaltitel: Risk Stratification for Hospital-Acquired Venous Thromboembolism: A Prospective Cohort Study

Diese Studie wird organisiert durch: Inselspital, Universitätsspital Bern (Sponsor). Die Studie wird am Inselspital und am Centre Hospitalier Universitaire Vaudois (CHUV) durchgeführt.

Sehr geehrte Dame, sehr geehrter Herr,

Wir interessieren uns für die Verbesserung der Behandlungsqualität auf der Inneren Medizin. Deshalb möchten wir Sie anfragen, ob Sie an einem multizentrischen Forschungsprojekt teilnehmen wollen. Das Endziel des Projektes ist, mit den Erkenntnissen dieser Studie die Entstehung von venösen Thromboembolien während oder nach einem Spitalaufenthalt besser verhindern zu können. Im Folgenden wird Ihnen das geplante Forschungsprojekt dargestellt. Aus Gründen der besseren Lesbarkeit wurde im Text die männliche Form gewählt.

1. Ziel der Studie

Diese Studie hat primär zum Ziel, das Risiko zur Entwicklung von venösen Thromboembolien bei hospitalisierten Patienten wie Ihnen zu untersuchen. Venöse Thromboembolien sind Blutgerinnsel in den Beinvenen (tiefe Venenthrombosen) oder den Lungenarterien (Lungenembolien). Ebenfalls möchten wir das Blutungsrisiko und die Selbständigkeit im Alltag nach einem Spitalaufenthalt untersuchen.

2. Auswahl der Personen, welche teilnehmen können

Es können alle Personen ab 18 Jahren teilnehmen, die für mindestens 24 Stunden auf der Inneren Medizin hospitalisiert sind.

3. Allgemeine Informationen zum Projekt

Anlässlich eines Spitalaufenthaltes kommt es bei einem kleinen Prozentsatz der Patienten (ca. 1-3%) zu einer Komplikation mit einer venösen Thromboembolie (daher auch Spital-assoziierte venöse Thromboembolie genannt). Zur Einschätzung des Risikos für venöse Thromboembolien wurden Risikomodelle (Scores) entwickelt, welche auf klinischen Charakteristika wie Alter, Begleiterkrankungen, oder der Mobilität beruhen. Diese Scores helfen Ihren behandelnden Ärzten bei der Entscheidung, ob gegebenenfalls präventive Massnahmen angezeigt sind.

In der Schweiz wurde vor Kurzem ein solcher Score zur Einschätzung des Risikos für venöse Thromboembolien bei entwickelt. Bevor dieser Score jedoch in der Alltagspraxis angewendet werden kann, muss er zuerst einer Population von hospitalisierten Patienten geprüft werden.

Im Rahmen einer Erkrankung oder eines Spitalaufenthaltes ist die Mobilität oft reduziert, was wiederum das Risiko für venöse Thromboembolien erhöht. Deshalb wird für die Einschätzung dieses Risikos auch die Mobilität berücksichtigt. Eine objektive Messung Ihrer körperlichen Aktivität während des Spitalaufenthaltes ist daher wichtig. Anhand eines Beschleunigungssensors, welcher um das Handgelenk getragen wird, kann die Quantität und Intensität der körperlichen Aktivität während der gesamten Hospitalisation objektiv gemessen werden.

Für diese Beobachtungsstudie, welche in mehreren Spitälern der Schweiz durchgeführt wird, werden insgesamt 1350 Patienten über einen Zeitraum von voraussichtlich 2 Jahren eingeschlossen.

Dieses Projekt wird so durchgeführt wie es die Gesetze in der Schweiz vorschreiben. Die zuständige Ethikkommission hat dieses Projekt geprüft und bewilligt.

4. Ablauf

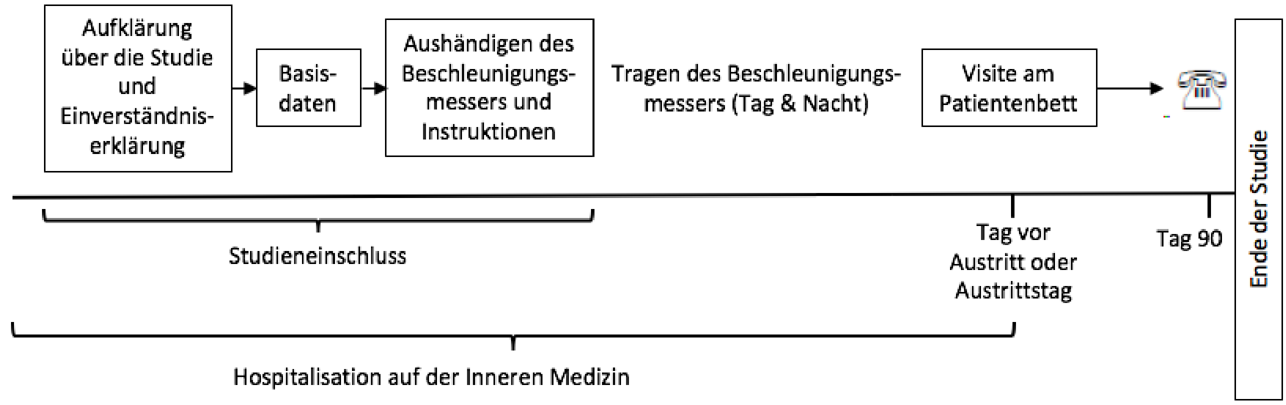

Nachdem Sie über die Studie informiert wurden und diese Studieninformation erhalten haben, werden Sie Zeit haben, um allfällige Fragen zu stellen. Falls Sie an der Studie teilnehmen möchten, werden wir Sie bitten, die Einverständniserklärung zu unterzeichnen. Der weitere Ablauf wird folgendermassen aussehen:

Während der Hospitalisation:

a) Die erste Studienvisite wird nach Eintritt auf die Innere Medizin vor Ort stattfinden (Dauer ca. 15 Minuten). Das Studienpersonal wird Ihre Kontaktinformationen aufnehmen und von Ihnen Informationen zu Ihrer Gesundheit sowie Ihrer Selbständigkeit in den Alltagsfunktionen erheben.
b) Bei Eintritt werden wir Ihnen einen Beschleunigungssensor aushändigen, den Sie am Handgelenk befestigen und während der gesamten Hospitalisation auf der Inneren Medizin (tags und nachts) tragen sollen. Dieser Beschleunigungssensor ähnelt einer kleinen, leichten Uhr und ermöglicht die Erfassung Ihrer Bewegungen, damit wir Ihre Mobilität während der Hospitalisation objektiv messen können (ähnlich wie ein Schrittzähler, kann aber nebst den Anzahl Schritten auch andere Formen der Bewegung messen).
c) Am Austrittstag (oder am Tag davor) erfolgt eine zweite Studienvisite vor Ort (Dauer ca. 15 Minuten). Dabei werden wir Informationen zum Spitalaufenthalt erheben und Ihnen den Beschleunigungssensor wieder abnehmen.
d) Zudem werden wir aus Ihren Krankenakten Informationen erfassen zu Ihnen und Ihren Erkrankungen, Medikamenten, Laborresultaten, Behandlungen, welche Sie während des aktuellen Spitalaufenthaltes erhalten haben, und Ihrer Mobilität.
e) Zusätzlich werden wir Ihren behandelnden Arzt über Ihre Studienteilnahme informieren und ihn zu Ihrer Mobilität und allfälligen anderen fehlenden Informationen befragen.

Nach Austritt aus dem Spital: 90 Tage nach Spitaleintritt wird ein **letzter** Studienkontakt per Telefon erfolgen, und es sind keine weiteren Studienvisiten notwendig.

f) ungefähr 90 Tage nach Spitaleintritt werden Sie telefonisch von unserem Studienpersonal kontaktiert, um Informationen zu Ihrer Gesundheit, zu allfälligen Komplikationen wie venösen Thromboembolien oder Blutungen, sowie zu Ihrer Selbständigkeit in Alltagsfunktionen zu erhalten (Dauer des Telefonats ca. 15 Minuten).
g) Gegebenenfalls werden wir zur Erfassung von eventuellen Komplikationen wie venöse Thromboembolien und Blutungen, welche wir in dieser Studie analysieren, Ihren nachbetreuenden Arzt (oder anderes Sie betreuendes Gesundheitspersonal z.B. aus dem Pflegeheim) kontaktieren und/oder diese Information aus Ihren Krankenakten erfassen.

Teilstudie: Untersuchung der Machbarkeit einer zukünftigen Studie zur Thromboseprophylaxe: Einen Teil der Studienteilnehmer werden wir über eine mögliche zukünftige Studie informieren, in welcher wir verschiedene prophylaktische Massnahmen zur Verhinderung von venösen Thromboembolien vergleichen möchten. Wir werden Sie fragen, ob Sie theoretisch bereit wären, an

einer solchen Studie mitzumachen. Diese Studie ist zum jetzigen Zeitpunkt nur hypothetisch, und es geht lediglich darum, dass wir die Machbarkeit einer solchen Studie für die Zukunft evaluieren; sie verpflichten sich somit zu gar nichts, und es geht nur um Ihre Ansicht dazu. Da wir diese Teilstudie nur bei einem Teil der Studienteilnehmer durchführen, kann es sein, dass Sie davon nicht betroffen sind. Diese Teilstudie würde gleich im Anschluss an die erste Studienvisite (nach Austeilen des Beschleunigungsmessers) erfolgen und dauert etwa 10-15 Minuten.

5. Nutzen
Sie werden persönlich keinen direkten Nutzen von der Teilnahme am Projekt haben. Die Resultate können wichtig sein für die Verbesserung der Behandlungsqualität für zukünftige hospitalisierte medizinische Patienten, damit die Spitalärzte das Risiko für Spital-assoziierte venöse Thromboembolien besser einschätzen und präventive Massnahmen gezielter einsetzen können.

6. Rechte
Sie nehmen freiwillig teil. Wenn Sie nicht mitmachen oder später Ihre Teilnahme zurückziehen wollen, müssen Sie dies nicht begründen. Ihre medizinische Behandlung/Betreuung ist unabhängig von Ihrem Entscheid gewährleistet. Sie dürfen jederzeit Fragen zur Teilnahme und zum Projekt stellen. Wenden Sie sich dazu bitte an die Person, die am Ende dieser Information genannt ist.

7. Pflichten
Als Teilnehmer ist es notwendig, dass Sie sich an die notwendigen Vorgaben und Anforderungen durch die Projektleitung und den Projektplan halten (Unterschreiben der Einverständniserklärung, Teilnahme an zwei Studienvisiten, Tragen des Beschleunigungssensors während der Hospitalisation, Teilnahme an der Telefonbefragung nach 90 Tagen).

8. Risiken und Belastungen für die Teilnehmenden
Durch das Projekt sind Sie nur geringfügigen Risiken wie mögliche Unannehmlichkeiten durch das Tragen des Beschleunigungssensors ausgesetzt (Hautirritation, Unbequemlichkeit des Armbands).

9. Andere Behandlungsmöglichkeiten
Sie müssen bei dieser Studie nicht teilnehmen. Sie erhalten die gleiche Behandlung und Betreuung im Spital, unabhängig davon, ob Sie in der Studie mitmachen oder nicht.

10. Ergebnisse aus der Studie
Der Prüfarzt wird Sie während des Projekts über alle neuen Erkenntnisse informieren, die den Nutzen oder Ihre Sicherheit und somit Ihre Einwilligung zur Teilnahme beeinflussen können.

11. Vertraulichkeit von Daten
Für dieses Projekt werden Ihre persönlichen und medizinischen Daten erfasst. Nur sehr wenige Fachpersonen werden Ihre unverschlüsselten Daten sehen, und zwar ausschliesslich, um Aufgaben im Rahmen des Projekts zu erfüllen. Bei der Datenerhebung zu Studienzwecken werden die Daten verschlüsselt. Verschlüsselung bedeutet, dass alle Bezugsdaten, die Sie identifizieren könnten (Name, Geburtsdatum), gelöscht und durch einen Schlüssel ersetzt werden. Die Schlüssel-Liste bleibt immer im Spital. Diejenigen Personen, die den Schlüssel nicht kennen, können daher keine Rückschlüsse auf Ihre Person ziehen. Bei einer Publikation sind die zusammengefassten Daten daher auch nicht auf Sie als Einzelperson rückverfolgbar. Ihr Name taucht niemals im Internet oder einer Publikation auf. Manchmal gibt es die Vorgabe bei einer Zeitschrift zur Publikation, dass Einzel-Daten (sogenannte Roh-Daten) übermittelt werden müssen.

Wenn Einzel-Daten übermittelt werden müssen, dann sind die Daten immer verschlüsselt und somit ebenfalls nicht zu Ihnen als Person rückverfolgbar. Alle Personen, die im Rahmen des Projekts Einsicht in Ihre Daten haben, unterliegen der Schweigepflicht. Die Vorgaben des Datenschutzes werden eingehalten und Sie als teilnehmende Person haben jederzeit das Recht auf Einsicht in Ihre Daten.

Die Datenerfassung erfolgt elektronisch anhand einer Datenbank für Forschungszwecke (RedCap der Clinical Trial Unit, Mittelstrasse 43, 3012 Bern, am Inselspital Bern).

Möglicherweise wird dieses Projekt durch die zuständige Ethikkommission oder durch die Institution, die das Projekt veranlasst hat, überprüft. Der Projektleiter muss eventuell Ihre persönlichen und medizinischen Daten für solche Kontrollen offenlegen. Alle Personen müssen absolute Vertraulichkeit wahren. Wir halten alle Vorgaben des Datenschutzes ein und werden Ihren Namen weder in einer Publikation noch im Internet öffentlich machen.

Es ist möglich, dass der Sie nachbehandelnde Arzt (oder anderes Gesundheitspersonal) kontaktiert wird, um Auskunft über den Gesundheitszustand im Rahmen der Studie zu geben.

12. Rücktritt
Sie können jederzeit aufhören und von dem Projekt zurücktreten, wenn Sie das wünschen. Die bis dahin erhobenen Daten und Proben werden noch verschlüsselt auswertet, weil das ganze Projekt sonst seinen Wert verliert. Nach der Auswertung werden Ihre Daten und vollständig anonymisiert, d.h. Ihre Schlüsselzuordnung wird vernichtet, so dass danach niemand mehr erfahren kann, dass die Daten und Proben ursprünglich von Ihnen stammten.

13. Entschädigung für Teilnehmende
Wenn Sie an diesem Projekt teilnehmen, bekommen Sie dafür keine Entschädigung. Es entstehen Ihnen oder Ihrer Krankenkasse keine Kosten durch die Teilnahme.

14. Haftung
Falls Sie durch das Projekt einen Schaden erleiden, haftet die Institution, die das Projekt veranlasst hat und für die Durchführung verantwortlich ist (Inselspital, Universitätsspital Bern). Die Voraussetzungen und das Vorgehen sind gesetzlich geregelt.
Wenn Sie einen Schaden erlitten haben, so wenden Sie sich bitte an den Projektleiter.

15. Finanzierung der Studie
Das Projekt wird durch Stiftungen sowie durch die teilnehmenden Kliniken finanziert.

16. Kontaktperson(en)
Bei allen Unklarheiten, Befürchtungen oder Notfällen, die während des Projekts oder danach auftreten, können Sie sich jederzeit an eine dieser Kontaktpersonen wenden.

Leiterin am Studienort:<br>**Dr. med. Christine Baumgartner**<br>Universitätsklinik für Allgemeine Innere Medizin<br>Inselspital, Universitätsspital Bern<br>Freiburgstrasse<br>3010 Bern	Das Studienteam von Dr. Baumgartner ist folgendermassen erreichbar:<br>Telefonnummer: **+41 (0)31 632 77 81** (zu Bürozeiten)<br>e-mail: **RISE@insel.ch**

Bei Fragen oder Bemerkungen dürfen Sie sich jederzeit an uns wenden.

Vielen Dank für Ihre wertvolle Mitarbeit. Mit freundlichen Grüssen

Dr. Christine Baumgartner

INSELSPITAL
UNIVERSITÄTSSPITAL BERN
HÔPITAL UNIVERSITAIRE DE BERNE

Einwilligungserklärung

Schriftliche Einwilligungserklärung zur Teilnahme an einem Studienprojekt
Bitte lesen Sie dieses Formular sorgfältig durch. Bitte fragen Sie, wenn Sie etwas nicht verstehen oder wissen möchten.

**BASEC-Nummer (nach Einreichung):**	
**Titel des Projekts (wissenschaftlich und Laiensprache)**:	Untersuchung des Risikos für Spital-assoziierte venöse Thromboembolien: eine prospektive Kohortenstudie
**verantwortliche Institution (Projektleitung mit Adresse)**:	Inselspital, Universitätsspital Bern<br>Dr. med. Christine Baumgartner<br>Freiburgstrasse<br>3010 Bern
**Ort der Durchführung**:	Inselspital, Universitätsspital Bern<br>Universitätsklinik für Allgemeine Innere Medizin<br>Freiburgstrasse<br>3010 Bern
**Leiter / Leiterin des Projekts am Studienort:**	Dr. med Christine Baumgartner
**Teilnehmerin/Teilnehmer:**<br>Name und Vorname in Druckbuchstaben:<br>Geburtsdatum:	☐ weiblich ☐ männlich

- Ich wurde vom unterzeichnenden Prüfarzt/ Prüfärztin/ Prüfperson mündlich und schriftlich über den Zweck, den Ablauf des Projekts, über mögliche Vor- und Nachteile sowie über eventuelle Risiken informiert.
- Ich nehme an diesem Projekt freiwillig teil und akzeptiere den Inhalt der zum oben genannten Projekt abgegebenen schriftlichen Information. Ich hatte genügend Zeit, meine Entscheidung zu treffen.
- Meine Fragen im Zusammenhang mit der Teilnahme an diesem Projekt sind mir beantwortet worden. Ich behalte die schriftliche Information und erhalte eine Kopie meiner schriftlichen Einwilligungserklärung.
- Ich bin einverstanden, dass mein Hausarzt über meine Teilnahme an dem Projekt informiert wird.
- Ich bin einverstanden, dass die zuständigen Fachleute der Projektleitung/ des Auftraggebers des Projekts und der für dieses Projekt zuständigen Ethikkommission zu Prüf- und Kontrollzwecken in meine unverschlüsselten Daten Einsicht nehmen dürfen, jedoch unter strikter Einhaltung der Vertraulichkeit.
- Bei Studienergebnissen oder Zufallsbefunden, die direkt meine Gesundheit betreffen, werde ich informiert. Wenn ich das nicht wünsche, informiere ich meinen Prüfarzt.
- Ich weiss, dass meine gesundheitsbezogenen und persönlichen Daten nur in verschlüsselter Form zu Forschungszwecken **für dieses Projekt** weitergegeben werden können.
- Im Fall einer Weiterbehandlung ausserhalb des Prüfzentrums ermächtige ich meine nachbehandelnden Ärzte oder anderes Gesundheitspersonal (z.B. Pflegepersonal aus Alters- und Pflegeheim, Rehabilitation), meine für das Projekt relevanten Nachbehandlungsdaten dem Prüfarzt zu übermitteln.
- Ich bin einverstanden, dass gegebenenfalls die von mir angegebene Kontaktperson im Verlauf der Studie kontaktiert und zu meinen für das Projekt relevanten Informationen befragt wird.

- Ich kann jederzeit und ohne Angabe von Gründen von der Teilnahme zurücktreten, ohne dass ich deswegen Nachteile bei der weiteren medizinischen Behandlung/Betreuung habe. Die bis dahin erhobenen Daten und Proben werden für die Auswertung des Projekts noch verwendet.
- Die Haftpflichtversicherung des Spitals/ der Institution kommt für allfällige Schäden auf.
- Ich bin mir bewusst, dass die in der Teilnehmerinformation genannten Pflichten einzuhalten sind. Im Interesse meiner Gesundheit kann mich der Leiter/ die Leiterin jederzeit ausschliessen.

Ort, Datum	Unterschrift Teilnehmerin/Teilnehmer

Bestätigung des Prüfarztes/der Prüfperson: Hiermit bestätige ich, dass ich dieser Teilnehmerin/ diesem Teilnehmer Wesen, Bedeutung und Tragweite des Projekts erläutert habe. Ich versichere, alle im Zusammenhang mit diesem Projekt stehenden Verpflichtungen gemäss dem geltenden Recht zu erfüllen. Sollte ich zu irgendeinem Zeitpunkt während der Durchführung des Projekts von Aspekten erfahren, welche die Bereitschaft der Teilnehmerin/ des Teilnehmers zur Teilnahme an dem Projekt beeinflussen könnten, werde ich sie/ ihn umgehend darüber informieren.

Ort, Datum	Name und Vorname der informierenden Prüfärztin/ des informierenden Prüfarztes/ der informierenden Prüfperson in Druckbuchstaben<br><br>Unterschrift der Prüfärztin/des Prüfarztes/der Prüfperson
