# Supplementary material for: Risk stratification for hospital-acquired venous thromboembolism in medical patients (RISE): Protocol for a prospective cohort study
Source: PLoS One. 2022 May 24;17(5):e0268833. doi: 10.1371/journal.pone.0268833 (PMC9128957; doi:10.1371/journal.pone.0268833)
Supplement: S4 File — (PDF) [file pone.0268833.s004.pdf]

Eligibility and Informed Consent

Patient ID	______________________
Current year	______________________<br>(format yyyy, e.g. 2020)
Screening date and time	______________________<br>(date format dd-mm-yyyy; time format hh:mm)
Sex	◯ male<br>◯ female
Year of Birth	______________________
Age (calculated)	______________________

Inclusion Criteria

age ≥18 years	◯ Yes ◯ No
admitted for hospitalization >24 hours on a general internal medicine ward	◯ Yes ◯ No<br>(defined as direct admission to internal medicine (NO transfers of patients previously hospitalized at the ICU))
informed consent as documented by signature	◯ Yes ◯ No

Exclusion Criteria

need for therapeutic anticoagulation	◯ Yes ◯ No<br>(e.g. atrial fibrillation/flutter with a CHADS2 score of ≥1 or CHA2DS2-VASc ≥2, mechanical heart valves, previous unprovoked VTE or provoked within the last 3 months)
life expectancy < 30 days	◯ Yes ◯ No
insufficient proficiency of the German or French language	◯ Yes ◯ No
unwilling to provide informed consent	◯ Yes ◯ No
prior enrolment in the study	◯ Yes ◯ No

Eligibility Determination

Patient is ELIGIBLE

Patient does NOT meet eligibility criteria

Eligibility criteria are incomplete!

Eligibility Calculation 0 = not eligible 1 = eligible
2 = eligibility criteria incomplete ________________

The patient is not eligible. Data entry for this patient is now complete, please save and exit form.

Informed Consent

Date of Informed Consent ________________
(date format dd-mm-yyyy)

Consent obtained from
◯ patient
◯ proxy

if consent obtained from proxy, please indicate reason for inability to provide informed consent
◯ cognitive impairment
◯ other psychiatric disorder

Demographic Characteristics

date of hospital admission	______________________ (date of admission to the current hospital; date format dd-mm-yyyy)
setting prior to admission	◯ home<br>◯ rehab<br>◯ nursing home<br>◯ other
if other, please indicate setting	______________________
body height [cm]	______________________ (if not documented in the patient chart directly ask the patient; if unknown, make the best estimate you can)
body weight [kg]	______________________ (if not documented in the patient chart, directly ask the patient; if unknown, make the best estimate you can)
BMI [kg/m2]	______________________

Comorbidities

history of myocardial infarction	◯ Yes<br>◯ No
peripheral vascular disease	◯ Yes<br>◯ No
cerebrovascular disease	◯ Yes<br>◯ No<br>(refers to: history of TIA or CVI)
dementia	◯ Yes<br>◯ No<br>(defined as: diagnosis of dementia in medical records (e.g. Alzheimer's disease, vascular dementia, dementia due to other disorders))
chronic obstructive pulmonary disease (copd)	◯ Yes<br>◯ No
connective tissue disease	◯ Yes<br>◯ No<br>(refers to: systemic lupus erythematodes (SLE), polymyositis, mixed connective tissue disease, polymyalgia rheumatica, moderate to severe rheumatoid arthritis)
peptic ulcer disease	◯ Yes<br>◯ No<br>(e.g. gastric ulcer or duodenal ulcer)
liver disease	◯ Yes, mild (cirrhosis without portal hypertension, chronic hepatitis)<br>◯ Yes, moderate to severe (cirrhosis with portal hypertension or presence of oesophageal / gastric varices, liver failure, liver necrosis, hepatorenal syndrome, veno-occlusive disease)<br>◯ No
diabetes mellitus	◯ Yes, without complications<br>◯ Yes, with complications (retinopathy, neuropathy, nephropathy)<br>◯ No<br>(refers to: diabetes mellitus with medication)
hemiplegia	◯ Yes<br>◯ No<br>(refers to: hemiplegia or paraplegia)
renal disease	◯ Yes, mild (kidney disease with creatinine < 266 umol/l, NO dialysis, NO transplantation)<br>◯ Yes, moderate to severe (creatinine >265 umol/l, dialysis, kidney transplantation, uremic syndrome)<br>◯ No

solid tumor or tumor with metastasis	◯ Yes, localized<br>◯ Yes, metastatic<br>◯ No<br>(consider every tumor that was treated within the last 5 years, excluding non-melanomatous skin cancers and in situ cervical carcinoma; myeloma or myelodysplastic syndrome are NOT considered solid tumors)
leukemia	◯ Yes<br>◯ No<br>(including myeloma or myelodysplastic syndrome)
lymphoma	◯ Yes<br>◯ No
AIDS	◯ Yes<br>◯ No<br>(defined as: diagnosis of AIDS in medical records, or HIV stage 3, or HIV and CD4 count < 200/ul)
prior bleeding within last 3 months	◯ Yes<br>◯ No<br>(defined as major bleeding (fatal bleeding, symptomatic bleeding at critical sites (intracranial, intraspinal, intraocular, retroperitoneal, intraarticular, pericardial, or intramuscular with compartment syndrome), or bleeding with a reduction of hemoglobin of at ≥20 g/L or bleeding leading to transfusion of ≥2 units of red blood cells) or clinically relevant non-major bleeding (associated with a medical intervention, unscheduled physician contact (visit or telephone call), or pain or impairment of activities of daily life))
inflammatory bowel disease	◯ Yes<br>◯ No<br>(refers to: colitis ulcerosa or morbus crohn)
blood dyscrasia	◯ Yes<br>◯ No<br>(defined as the presence of any bleeding disorder, e.g. hemophilia, von Willbrand disease, idiopathic thrombopenia (but NOT liver disease))
anemia	◯ Yes<br>◯ No<br>(defined as: hemoglobin value < 120g/l in women (< 110g/l if pregnant), < 130g/l in men)
number of comorbidities (enter number)	______________________________<br>(only count chronic conditions as listed on the patient's diagnoses list)

principal diagnosis at admission, please indicate category	◯ heart failure<br>◯ acute ischemic heart disease<br>◯ arrhythmia<br>◯ venous thromboembolism<br>◯ stroke/TIA<br>◯ COPD exacerbation<br>◯ pneumonia<br>◯ infection, sepsis<br>◯ gastro-intestinal disorder<br>◯ liver disorder<br>◯ renal disorder<br>◯ nutrition or metabolic disorder<br>◯ adverse drug event<br>◯ neoplasm<br>◯ neurologic disorder (not stroke related<br>◯ other
of other, please specify (free text)	__________________________________
presence of isolation precaution	◯ Yes<br>◯ No<br>(isolation e.g. due to MRSA, VRE, COVID-19, etc)

VTE risk factors

Items of the risk assessment models	
previous venous thromboembolism (VTE)	◯ Yes<br>◯ No<br>(only prior deep vein thrombosis, pulmonary embolism (do not consider cerebral or splanchnic vein thrombosis))
hypercoagulable state/thrombophilia	◯ Yes<br>◯ No<br>(defined as: antithrombin deficiency, APC resistence, protein C or protein S deficiency, faktor V Leiden, G20210A prothrombin-mutation, antiphospholipid syndrome)
history of any cancer within last 5 years	◯ Yes<br>◯ No<br>(excluding non-melanoma skin cancer)
active cancer	◯ Yes<br>◯ No<br>(defined as: metastatic cancer, or cancer treated with radiotherapy/chemotherapy/immunotherapy, or cancer surgery within last 6 months (also relates to myeloma or myelodysplastic syndrome))
myeloproliferative syndrome	◯ Yes<br>◯ No<br>(refers to: essential thrombocytopenia, polycythemia vera, primary myelofibrosis, chronic myeloic leukemia (CML); does NOT refer to myeloma or myelodysplastic syndrome)
cardiac failure	◯ Yes<br>◯ No<br>(acute or chronic cardiac failure, definend as: diagnosis of heart failure in medical records (HF-rEF or HF-pEF) or a known left ventricular ejection fraction (EF) of < 40%)
respiratory failure (chronic or acute)	◯ Yes<br>◯ No<br>(acute or chronic respiratory failure, defined as: need for supplemental oxygen)
acute infection	◯ Yes<br>◯ No<br>(defined as: acute infection during current hospitalization, or infection that has led to hospitalization, including sepsis)
COVID-19	◯ Yes, confirmed by PCR / serology<br>◯ Highly suspected, but PCR / serology negative<br>◯ No

acute rheumatologic disorder	◯ Yes<br>◯ No<br>(defined as: a diagnosis of rheumatoid arthritis, vasculitis (e.g., granulomatosis with polyangiitis [Wegener], Behçet disease, etc.), or connective tissue disease (e.g., systemic lupus erythematodes [SLE], Sjögren's syndrome, myositis, or systemic sclerosis))
immobilization ≥7 days	◯ Yes<br>◯ No<br>(defined as: being confined to bed or chair with or without bathroom privileges for 7 or more days days immediately prior to and during hospital admission)
immobilization ≥3 days	◯ Yes<br>◯ No<br>(defined as: complete bedrest or inability to walk for >30min per day for ≥3d)
reduced mobility for at least 3 days	◯ Yes<br>◯ No<br>(defined as: anticipated bed rest with bathroom privileges (either due to patient's limitations or on physicians order) for at least 3d)
recent ischemic or hemorrhagic stroke (≤3 months)	◯ Yes, ischemic<br>◯ Yes, hemorrhagic<br>◯ Yes, both ischemic and hemorrhagic stroke<br>◯ No<br>(please note that hemorrhagic stroke refers to intracerebral hemorrhage)
if yes, date of stroke (if available)	______________________________<br>(if more than one event, please indicate date of last event)
recent myocardial infarction (≤1 month)	◯ Yes<br>◯ No<br>(defined as any diagnosis of myocardial infarction, STEMI, NSTEMI)
if yes, date of myocardial infarction if available	______________________________
recent (≤1 month) trauma	◯ Yes<br>◯ No<br>(defined as: severe trauma resulting in a hospitalization)
if yes, date of trauma if available	______________________________
recent (≤1 month) surgery	◯ Yes<br>◯ No<br>(for surgery: only surgery lasting >60min (i.e. do not consider port-à-cath or pace maker implantations))

if yes, date of surgery if available	__________________
ongoing hormonal treatment	◯ Yes<br>◯ No<br>(refers to: contraception, post-menopausal hormone therapy, antitumor therapy containing estrogen, ethinylestradion, estradiol))
lower extremity paralysis/paresis	◯ Yes<br>◯ No<br>(if documented in the medical records)
stay in intensive care unit/ intermediate care unit	__________________<br>(during the current hospitalization)
nephrotic syndrome	◯ Yes<br>◯ No<br>(defined as: diagnosis of nephrotic syndrome in medical records)
recent travel >6h (within last 7 days)	◯ Yes<br>◯ No
pregnancy	◯ Yes<br>◯ No
acute dehydration	◯ Yes<br>◯ No<br>(check if dehydration or hypovolemia is mentioned in medical records, or prerenal kidney insufficiency)
chronic venous insufficiency	◯ Yes<br>◯ No<br>(check in medical records or ask patient about varicose veins or chronic venous insufficiency)

Other potential VTE risk factors

heart rate [beats per minute]	__________________<br>(Take the first available measurement at admission)
body temperature [°C]	__________________<br>(Take the first available measurement at admission)
leg edema	◯ Yes<br>◯ No<br>(check if leg edema mentioned in the medical records (physical examination findings))
active tobacco use	◯ Yes<br>◯ No

Contraindications to pharmacological TPX

known hypersensitivity to heparin or heparin induced thrombocytopenia (HIT)	◯ Yes<br>◯ No
liver failure	◯ Yes<br>◯ No<br>(defined as: diagnosis of liver failure in medical records, or cirrhosis with spontaneous INR>2)
any active bleeding	◯ Yes<br>◯ No<br>(defined as any signs of active bleeding (presence of melena, bright red blood in stool, macroscopic hematuria, or active bleeding at other sites) at the time of presentation)
if yes, date of bleeding event	______________________________
hemorrhagic transformation of acute ischemic stroke	◯ Yes<br>◯ No
if yes, date of hemorrhagic transformation	______________________________

Laboratory findings (as available)

Laboratory findings at admission (as available)

platelet count [G/l] at admission

____
(Take the first available measurement at admission)

INR at admission

____
(Take the first available measurement at admission)

serum creatinine [µmol/l] at admission

____
(Take the first available measurement at admission)

creatinine clearance [ml/min]

____

hemoglobin [g/l] at admission

____
(Take the first available measurement at admission)

leukocyte count [G/l] at admission

____
(Take the first available measurement at admission)

CRP [mg/l] at admission

____
(Take the first available measurement at admission)

D-dimer [µg/l or ng/ml] at admission

____
(Take the first available measurement at admission, leave blank if not available)

Medications at baseline

Medications at admission

aspirin	◯ Yes<br>◯ No
other anti-platelet therapy	◯ Yes<br>◯ No<br>(e.g. clopidogrel, ticagrelor, prasugrel)
non-steroidal anti-inflammatory drugs (NSAIDs)	◯ Yes<br>◯ No<br>(e.g. ibuprofen, diclofenac, naproxen, etc.)

Treatments since admission

thromboprophylaxis started	◯ Yes<br>◯ No<br>(refers to any pharmacological or mechanical thromboprophylaxis)
if yes, start date	__________________________
if yes, indicate type of thromboprophylaxis	◯ pharmacological thromboprophylaxis<br>◯ mechanical thromboprophylaxis

Mobility assessment at admission

use of walking aids within last 2 weeks before admission	◯ Yes<br>◯ No
patient is bedridden or unable to walk	◯ no<br>◯ yes, before hospital admission and since study inclusion (at least one week)<br>◯ yes, since study inclusion
current (hospital) ambulation	◯ walk alone<br>◯ walk with the help of one person<br>◯ walk with the help of 2 persons<br>◯ transfer only from bed to chair<br>◯ bedridden
where does the patient eat (location)?	◯ in bed<br>◯ sitting at the edge of the bed<br>◯ on the table (out of bed)<br>◯ does not eat
where does the patient eliminate (urine or stool) (location)?	◯ in bed<br>◯ on a chair besides the bed (out of bed)<br>◯ bathroom
where does the patient wash him/herself?	◯ in bed<br>◯ on a chair in front of a sink (out of bed)<br>◯ shower
physical therapy prescribed for mobilization	◯ Yes<br>◯ No

Barthel Index at admission

This refers to the patient's automoy in the activities of daily living prior to hospitalization (i.e. during the 2 weeks prior to admission)

BI personal hygiene prior to admission	◯ The patient is unable to attend to personal hygiene and is dependent in all aspects.<br>◯ Assistance is required in all steps of personal hygiene, but patient able to make some contribution.<br>◯ Some assistance is required in one or more steps of personal hygiene.<br>◯ Patient is able to conduct his/her own personal hygiene but requires minimal assistance before and/or after the operation.<br>◯ The patient can wash his/her hands and face, comb hair, clean teeth and shave. A male patient may use any kind of razor but must insert the blade, or plug in the razor without help, as well as retrieve it from the drawer or cabinet. A female patient must apply her own make-up, if used, but need not braid or style her hair.
BI bathing prior to admission	◯ Total dependence in bathing self.<br>◯ Assistance is required in all aspects of bathing, but patient is able to make some contribution.<br>◯ Assistance is required with either transfer to shower/bath or with washing or drying; including inability to complete a task because of condition or disease, etc.<br>◯ Supervision is required for safety in adjusting the water temperature, or in the transfer.<br>◯ The patient may use a bathtub, a shower, or take a complete sponge bath. The patient must be able to do all the steps of whichever method is employed without another person being present.
BI feeding prior to admission	◯ Dependent in all aspects and needs to be fed, nasogastric needs to be administered.<br>◯ Can manipulate an eating device, usually a spoon, but someone must provide active assistance during the meal.<br>◯ Able to feed self with supervision. Assistance is required with associated tasks such as putting milk/sugar into tea, salt, pepper, spreading butter, turning a plate or other "set up" activities.<br>◯ Independence in feeding with prepared tray, except may need meat cut, milk carton opened or jar lid etc. The presence of another person is not required.<br>◯ The patient can feed self from a tray or table when someone puts the food within reach. The patient must put on an assistive device if needed, cut food, and if desired use salt and pepper, spread butter, etc.

BI toilet transfers prior to admission	◯ Fully dependent in toileting.<br>◯ Assistance required in all aspects of toileting.<br>◯ Assistance may be required with management of clothing, transferring, or washing hands.<br>◯ Supervision may be required for safety with normal toilet. A commode may be used at night but assistance is required for emptying and cleaning.<br>◯ The patient is able to get on/off the toilet, fasten clothing and use toilet paper without help. If necessary, the patient may use a bed pan or commode or urinal at night, but must be able to empty it and clean it.
BI stair climbing prior to admission	◯ The patient is unable to climb stairs.<br>◯ Assistance is required in all aspects of stair climbing, including assistance with walking aids.<br>◯ The patient is able to ascend/descend but is unable to carry walking aids and needs supervision and assistance.<br>◯ Generally no assistance is required. At times supervision is required for safety due to morning stiffness, shortness of breath, etc.<br>◯ The patient is able to go up and down a flight of stairs safely without help or supervision. The patient is able to use hand rails, cane or crutches when needed and is able to carry these devices as he/she ascends or descends.
BI dressing prior to admission	◯ The patient is dependent in all aspects of dressing and is unable to participate in the activity.<br>◯ The patient is able to participate to some degree, but is dependent in all aspects of dressing.<br>◯ Assistance is needed in putting on, and/or removing any clothing.<br>◯ Only minimal assistance is required with fastening clothing such as buttons, zips, bra, shoes, etc.<br>◯ The patient is able to put on, remove, corset, braces, as prescribed.
BI bowel control prior to admission	◯ The patient is bowel incontinent.<br>◯ The patient needs help to assume appropriate position, and with bowel movement facilitatory techniques.<br>◯ The patient can assume appropriate position, but cannot use facilitatory techniques or clean self without assistance and has frequent accidents. Assistance is required with incontinence aids such as pad, etc.<br>◯ The patient may require supervision with the use of suppository or enema and has occasional accidents.<br>◯ The patient can control bowels and has no accidents, can use suppository, or take an enema when necessary.

BI bladder control prior to admission	◯ The patient is dependent in bladder management, is incontinent, or has indwelling catheter.<br>◯ The patient is incontinent but is able to assist with the application of an internal or external device.<br>◯ The patient is generally dry by day, but not at night and needs some assistance with the devices.<br>◯ The patient is generally dry by day and night, but may have an occasional accident or need minimal assistance with internal or external devices.<br>◯ The patient is able to control bladder day and night, and/or is independent with internal or external devices.
BI ambulation prior to admission	◯ Dependent in ambulation.<br>◯ Constant presence of one or more assistant is required during ambulation.<br>◯ Assistance is required with reaching aids and/or their manipulation. One person is required to offer assistance.<br>◯ The patient is independent in ambulation but unable to walk 50 metres without help, or supervision is needed for confidence or safety in hazardous situations.<br>◯ The patient must be able to wear braces if required, lock and unlock these braces assume standing position, sit down, and place the necessary aids into position for use. The patient must be able to crutches, canes, or a walkarette, and walk 50 metres without help or supervision.
if dependent in ambulation, has the patient been trained in wheelchair management?	◯ Yes<br>◯ No
if unable to walk and trained in wheelchair management, BI ambulation wheelchair	◯ Dependent in wheelchair ambulation.<br>◯ Patient can propel self short distances on flat surface, but assistance is required for all other steps of wheelchair management.<br>◯ Presence of one person is necessary and constant assistance is required to manipulate chair to table, bed, etc.<br>◯ The patient can propel self for a reasonable duration over regularly encountered terrain. Minimal assistance may still be required in "tight corners" or to negotiate a kerb 100mm high.<br>◯ To propel wheelchair independently, the patient must be able to go around corners, turn around, manoeuvre the chair to a table, bed, toilet, etc. The patient must be able to push a chair at least 50 metres and negotiate a kerb.

BI chair/bed transfers prior to admission	◯ Unable to participate in a transfer. Two attendants are required to transfer the patient with or without a mechanical device.<br>◯ Able to participate but maximum assistance of one other person is require in all aspects of the transfer.<br>◯ The transfer requires the assistance of one other person. Assistance may be required in any aspect of the transfer.<br>◯ The presence of another person is required either as a confidence measure, or to provide supervision for safety.<br>◯ The patient can safely approach the bed walking or in a wheelchair, lock brakes, lift footrests, or position walking aid, move safely to bed, lie down, come to a sitting position on the side of the bed, change the position of the wheelchair, transfer back into it safely and/or grasp aid and stand. The patient must be independent in all phases of this activity.
The Barthel Index is not complete yet!	
Barthel Index	______________________________

Activity monitor: hand out

recording start date	______________________
wrist side	◯ right<br>◯ left
serial number of activity monitor	______________________<br>(enter 6 digit serial number)

Don't forget to push the button to start recording!

The baseline data collection for the main study is now completed, please continue to enter the data for the substudies.

Substudy: Braden Scale

Sensory perception, ability to respond to pressure related discomfort effectively	◯ completely limited: unresponsive to painful stimuli due to diminished consciousness or sedation, OR limited ability to feel pain over most of body<br>◯ very limited: responds only to painful stimuli, cannot communicate discomfort except by moaning or restlessness, OR has sensory impairment limiting ability to feel pain/discomfort over half of body<br>◯ slightly limited: responds to verbal commands, but cannot always communicate discomfort or need to be turned, OR has sensory impairment limiting ability to feel pain/discomfort in 1-2 extremities<br>◯ no impairment: no sensory deficit limiting expression of discomfort
Moisture, degree to which skin is exposed to moisture	◯ constantly moist; skin is kept moist almost constantly by perspiration, urine, etc; dampness detected every time patient is moved/turned<br>◯ very moist; skin is often but not always moist; linen must be changed at least once a shift<br>◯ occasionally moist; requiring extra linen change approximately once a day<br>◯ rarely moist; skin usually dry, linen only requires changing at routine intervals
Activity, degree of physical activity	◯ bedfast; confined to bed<br>◯ chairfast; ability to walk severely limited or non-existent; cannot bear own weight and/or must be assisted into chair/wheelchair<br>◯ walks occasionally; during day but for very short distances with or without assistance; spends majority of shift in bed/chair<br>◯ walks often; walks outside room ≥2x/day and inside room at least once every 2 hrs during waking hours
Mobility, ability to change and control position	◯ completely immobile; does not make even slight changes in body or extremity position without assistance<br>◯ very limited; makes occasional slight changes in body or extremity position but unable to make frequent or significant changes independently<br>◯ slightly limited; makes frequent though slight changes in body or extremity position independently<br>◯ no limitation; makes major and frequent changes in position without assistance

Nutrition, usual food intake pattern	◯ very poor; never eats complete meal; rarely eats >⅓ of any food offered; eats ≤2 servings protein (meat or dairy) daily; takes fluids poorly; does not take liquid dietary supplement; or is NPO and/or maintained on clear liquids or IV for >5 days<br>◯ probably indadequate; rarely eats complete meal and generally eats only about ½ of any food offered; protein intake includes only 3 servings of meat or dairy products daily; occasionally will take dietary supplement; or receives less than optimum amount of liquid diet/tube feeding<br>◯ adequate; eats over half of most meals; eats 4 servings protein (meat or dairy) daily; occasionally refuses meal but will usually take supplement when offered; or is on a tube feeding/TPN regimen which probably meets most of nutritional needs<br>◯ excellent; eats most of every meal; never refuses a meal; usually eats ≥4 servings of meat and dairy products; occasionally eats between meals; supplementation not required
Friction and shear	◯ problem; requires moderate to maximum assistance in moving; complete lifting without sliding against sheets is impossible; frequently slides down in bed or chair, requiring frequent repositioning with maximum assistance; spasticity, contractures, or agitation leads to almost constant friction<br>◯ potential problem; moves feebly or requires minimum assistance; during a move, skin probably slides to some extent against sheets, chair restraints, or other devices; maintains relatively good position in chair or bed most of the time but occasionally slides down<br>◯ no apparent problem; moves in bed/chair independently, has sufficient muscle strength to lift up completely during move, maintains good position in bed/chair
The Braden Scale is not complete yet!	
Braden score	______________________________

Substudy: hypothetical inclusion in future VTE RCT

Hypothetical inclusion criteria

hospitalized for acute non-surgical illness	◯ Yes<br>◯ No<br>(no prior or planned surgery was the reason for the current hospitalization)
expected duration of hospital stay ≥48h	◯ Yes<br>◯ No<br>(indicate "no" if the patinet is expected to be discharged soon, e.g. because he/she only needs to wait for a procedure, or only needs short (e.g. 24h) surveillance)
simplified Geneva score	______________________
High VTE risk as assessed by the simplified Geneva score (≥3 points)	______________________

Hypothetical exclusion criteria

known hypersensitivity to heparin or heparin induced thrombocytopenia (HIT)	______________________
creatinine clearance < 15ml/min	______________________
platelet count < 50G/l	______________________
liver failure with spontaneous INR>2.0	______________________
active bleeding	______________________
hemorrhagic transformation of acute ischemic troke	______________________
fibrinolysis/thrombolysis within 24h	◯ Yes<br>◯ No
any type of anticoagulation given for >48h immediately before study enrolment	◯ Yes<br>◯ No<br>(this also refers to prophylactic dose anticoagulation (i.e. pharmacological thromboprophylaxis))
hospitalized for >72h prior to study enrolment	◯ Yes<br>◯ No<br>(before study enrolment)

major surgery within last month	__________________________________
planned surgery during this hospitalization	◯ Yes<br>◯ No
known pregnancy	__________________________________
patient is currently breastfeeding	◯ Yes<br>◯ No

Eligibility for Substudy

Automatic eligibility Calculation for Substudy 0 = not eligible 1 = eligible 2 = eligibility criteria incomplete	__________________________________<br>(if patient is eligible for stubstudy, please complete substudy form 2 at the discharge visit)
Please manually confirm eligibility for substudy	◯ not eligible<br>◯ eligible

The baseline visit is now completed, thank you!

Mobility assessment by physician

Mobility assessment by Physician: to be filled in on day2 --> ask the physician to estimate the patient's mobility as of today

date of mobility assessment by physician	______________________ (date format dd-mm-yyyy)
physician's perception of patient's mobility	◯ no ambulation<br>◯ out of bed to chair<br>◯ out of bed to ambulate once daily<br>◯ out of bed to ambulate twice daily<br>◯ out of bed to ambulate three times daily<br>◯ ambulation more than three times daily<br>(ask physician on day 2 - do not mention relation to VTE prevention study!)
physician estimation: immobilization ≥7 days	◯ Yes<br>◯ No<br>(defined as: being confined to bed or chair with or without bathroom privileges for 7 or more days days immediately prior to and during hospital admission)
physician estimation: immobilization ≥3 days	◯ Yes<br>◯ No<br>(defined as: complete bedrest or inability to walk for >30min per day for ≥3d)
physician estimation: reduced mobility for at least 3 days	◯ Yes<br>◯ No<br>(defined as: anticipated bed rest with bathroom privileges (either due to patient's limitations or on physicians order) for at least 3d)

The mobility assessment by the phyisician is now completed, thank you! The next follow-up visit will be the day prior to or the day of discharge.

Discharge information

date of discharge visit	______________ (date format dd-mm-yyyy)
hemoglobin at discharge [g/l]	______________ (refers to the last hemoglobin value that was measured prior to the discharge visit)
date of discharge from internal medicine	______________ (date format dd-mm-yyyy)
discharge location	◯ home<br>◯ home with aid (Spitex/ CMS / OSAD)<br>◯ return to nursing home<br>◯ new institutionalization<br>◯ rehabilitation<br>◯ death<br>◯ other acute care hospital<br>◯ transfer to another unit of the same hospital<br>◯ palliative care structure<br>◯ other<br>(Ferienbett / lit de vacances is considered as new institutionalization)
if other discharge location, please specify	______________
Please collect medical records, death certificates and autopsy reports. Please complete the discharge visit, and don't forget to complete end of study form!	
if the patient is transferred to another unit, please indicate the division	◯ surgery<br>◯ ICU<br>◯ other
if other unit, please specify	______________
date of discharge from the hospital	______________ (date format dd-mm-yyyy)
discharge follow-up visit completed	◯ Yes<br>◯ No
if no, reason for non-completion	◯ death<br>◯ withdrawal<br>◯ patient discharged early<br>◯ other
indicate reason	______________
Don't Forget to complete end of study form!	

Follow-up information

date of follow-up phone call (90 +/- 5d)	________________ (date format dd-mm-yyyy)
contacts made for follow-up phone call	☐ patient<br>☐ relative / friend / legal representative<br>☐ general practitioner / other health care professional
follow-up completed	◯ Yes<br>◯ No

Don't forget to complete end of study form!

Please make sure to complete the date of the follow-up visit!

Outcomes

occurrence of venous thromboembolism (VTE)	◯ Yes<br>◯ No<br>(was a VTE (pumonary embolism or deep vein thrombosis) diagnosed during the stay in internal medicine (for the discharge visit) or after discharge from internal medicine (for the 90day visit)?)
if yes, type of VTE event	◯ pulmonary embolism<br>◯ deep vein thrombosis (DVT)<br>◯ both pulmonary embolism and DVT
if yes, date of VTE event	____________________<br>(date format dd-mm-yyyy)
occurrence of a second VTE event (recurrence)	◯ Yes<br>◯ No
if yes, type of VTE event	◯ pulmonary embolism<br>◯ deep vein thrombosis (DVT)<br>◯ both pulmonary embolism and DVT
if yes, date of second VTE event	____________________<br>(date format dd-mm-yyyy)
occurrence of bleeding	◯ Yes<br>◯ No<br>(did a bleeding event occur during the stay in internal medicine (for the discharge visit) or after discharge from internal medicine (for the 90day visit)?)
if yes, localization of bleeding	◯ intracranial<br>◯ intraspinal<br>◯ intraocular<br>◯ retroperitoneal<br>◯ intraarticular<br>◯ pericardial<br>◯ intramuscular with compartment syndrome<br>◯ intramuscular without compartment syndrome<br>◯ gastrointestinal<br>◯ urogenital<br>◯ epistaxis<br>◯ other<br>◯ unknown
if other, indicate location	____________________
did the bleeding lead to a reduction of hemoglobin of ≥20g/l?	◯ no<br>◯ yes<br>◯ unknown

did the bleeding lead to transfusion of ≥2 units of packed red blood cells?	◯ Yes<br>◯ No
the bleeding event was related to (check all that apply)	☐ medical intervention<br>☐ unscheduled physician contact (visit or phone call) if bleeding occurred outside of the hospital<br>☐ pain<br>☐ impairment in activities of daily life
date of bleeding event	____________________<br>(date format dd-mm-yyyy)
ocurrence of second bleeding event	◯ Yes<br>◯ No
if yes, localization of bleeding	◯ intracranial<br>◯ intraspinal<br>◯ intraocular<br>◯ retroperitoneal<br>◯ intraarticular<br>◯ pericardial<br>◯ intramuscular with compartment syndrome<br>◯ intramuscular without compartment syndrome<br>◯ gastrointestinal<br>◯ urogenital<br>◯ epistaxis<br>◯ other<br>◯ unknown
if other, indicate location	____________________
did the bleeding lead to a reduction of hemoglobin of ≥20g/l?	◯ no<br>◯ yes<br>◯ unknown
did the bleeding lead to transfusion of ≥2 units of packed red blood cells?	◯ Yes<br>◯ No
the bleeding event was related to (check all that apply)	☐ medical intervention<br>☐ unscheduled physician contact (visit or phone call) if bleeding occurred outside of the hospital<br>☐ pain<br>☐ impairment in activities of daily life
date of bleeding event	____________________<br>(date format dd-mm-yyyy)
ocurrence of third bleeding event	◯ Yes<br>◯ No

if yes, localization of bleeding	◯ intracranial<br>◯ intraspinal<br>◯ intraocular<br>◯ retroperitoneal<br>◯ intraarticular<br>◯ pericardial<br>◯ intramuscular with compartment syndrome<br>◯ intramuscular without compartment syndrome<br>◯ gastrointestinal<br>◯ urogenital<br>◯ epistaxis<br>◯ other<br>◯ unknown
if other, indicate location	__________________________________
did the bleeding lead to a reduction of hemoglobin of ≥20g/l?	◯ no<br>◯ yes<br>◯ unknown
did the bleeding lead to transfusion of ≥2 units of packed red blood cells?	◯ Yes<br>◯ No
the bleeding event was related to (check all that apply)	☐ medical intervention<br>☐ unscheduled physician contact (visit or phone call) if bleeding occurred outside of the hospital<br>☐ pain<br>☐ impairment in activities of daily life
date of bleeding event	__________________________________<br>(date format dd-mm-yyyy)
ocurrence of a fourth bleeding event	◯ Yes<br>◯ No
if yes, localization of bleeding	◯ intracranial<br>◯ intraspinal<br>◯ intraocular<br>◯ retroperitoneal<br>◯ intraarticular<br>◯ pericardial<br>◯ intramuscular with compartment syndrome<br>◯ intramuscular without compartment syndrome<br>◯ gastrointestinal<br>◯ urogenital<br>◯ epistaxis<br>◯ other<br>◯ unknown
if other, indicate location	__________________________________
did the bleeding lead to a reduction of hemoglobin of ≥20g/l?	◯ no<br>◯ yes<br>◯ unknown

did the bleeding lead to transfusion of ≥2 units of packed red blood cells?	◯ Yes<br>◯ No
the bleeding event was related to (check all that apply)	☐ medical intervention<br>☐ unscheduled physician contact (visit or phone call) if bleeding occurred outside of the hospital<br>☐ pain<br>☐ impairment in activities of daily life
date of bleeding event	______________________<br>(date format dd-mm-yyyy)
death	◯ Yes<br>◯ No
if yes, date of death	______________________<br>(date format dd-mm-yyyy)

Please make sure to fill in the correct date of the outcome event!

Please collect medical records, death certificates and autopsy reports. Please complete this follow-up visit, and don't forget to complete end of study form!

Please collect all documentation (medical records, laboratory values, radiology reports, etc.) concerning the clinical outcome events!

Thromboprophylaxis during hospital stay

Pharmacologic thromboprophylaxis during hospital stay

Pharmacologic thromboprophylaxis (TPX) prescribed during hospital stay	◯ Yes<br>◯ No
if yes, start date TPX	__________________________________<br>(date format dd-mm-yyyy)
if yes, which one?	◯ dalteparine<br>◯ nadroparine<br>◯ enoxaparine<br>◯ heparin<br>◯ fondaparinux<br>◯ prophylactic dose DOAC (apixaban or rivaroxaban)<br>◯ other
if other, indicate drug	__________________________________
end date TPX	__________________________________<br>(date format dd-mm-yyyy; if end date of TPX is unknown (e.g. because patient was discharged on TPX), add 09-09-9999)
if first TPX prescription has been stopped, has a 2nd pharmacologic TPX been prescribed during hospital stay?	◯ Yes<br>◯ No
if yes, start date 2nd TPX	__________________________________<br>(date format dd-mm-yyyy)
if yes, which one?	◯ dalteparine<br>◯ nadroparine<br>◯ enoxaparine<br>◯ heparin<br>◯ fondaparinux<br>◯ prophylactic dose DOAC (apixaban or rivaroxaban)<br>◯ other
if other, indicate drug	__________________________________<br>(date format dd-mm-yyyy)
end date 2nd TPX	__________________________________<br>(date format dd-mm-yyyy)
if 2nd TPX prescription has been stopped, has a 3rd pharmacologic TPX been prescribed during hospital stay?	◯ Yes<br>◯ No

if yes, start date 3rd TPX	______________________ (date format dd-mm-yyyy)
if yes, which one?	◯ dalteparine<br>◯ nadroparine<br>◯ enoxaparine<br>◯ heparin<br>◯ fondaparinux<br>◯ prophylactic dose DOAC (apixaban or rivaroxaban)<br>◯ other
if other, indicate drug	______________________
end date 3nd TPX	______________________ (date format dd-mm-yyyy)
therapeutic anticoagulation initiated during hospital stay	◯ Yes<br>◯ No
therapeutic anticoagulant agent prescribed during hospital stay	☐ DOAC (apixaban, rivaroxaban, dabigatran, edoxaban)<br>☐ vitamin K antagonist (e.g. sintrom, marcoumar)<br>☐ low molecular weight heparin (e.g. dalteparin, nadroparin, enoxaparin)<br>☐ unfractionated heparin
if DOAC, which one?	◯ rivaroxaban (Xarelto)<br>◯ apixaban (Eliquis)<br>◯ edoxaban (Lixiana)<br>◯ dabigatran (Pradaxa)
if yes, start date	______________________ (date format dd-mm-yyyy)
if yes, indicate reason	◯ venous thromboembolism<br>◯ atrial fibrillation<br>◯ prosthetic valve<br>◯ other
if therapeutic anticoagulation was stopped again during the hospitalization, enter stop date (otherwise leave blank)	______________________
if other, indicate reason (free text)	______________________

Mechanical thromboprophylaxis since admission

Mechanical thromboprophylaxis prescribed since admission	◯ no<br>◯ yes, compression stockings or bandages<br>◯ yes, intermittent pneumatic compression devices

Other treatments during hospital stay

did the patient receive red blood cell transfusions during current hospital stay?	◯ no<br>◯ yes, 1 red blood cell unit<br>◯ yes, 2 red blood cell units<br>◯ yes, 3 or more red blood cell units
if 3 or more, how many?	______________________
if yes, date of first transfusion	______________________
date of 2nd transfusion	______________________
date of 3rd transfusion	______________________
date of 4th transfusion	______________________<br>(leave blank if no 4th transfusion)
date of 5th transfusion	______________________<br>(leave blank if no 5th transfusion)
date of 6th transfusion	______________________<br>(leave blank if no 6th transfusion)
date of 7th transfusion	______________________<br>(leave blank if no 7th transfusion)
central venous catheter	◯ Yes<br>◯ No
surgery during hospital stay	◯ Yes<br>◯ No<br>(for surgery: only surgery lasting >60min (i.e. do not consider port-à-cath or pace maker implantations))
if yes, type of surgery?	______________________

Activity monitor: collect

did the patient stop using the activity monitor prior to study end (i.e. at least 1 day prior to the discharge visit)?	◯ Yes<br>◯ No<br>(click yes if patient stopped using the activity monitor)
if yes, please indicate reason	◯ patient refused<br>◯ patient withdrawal due to local discomfort<br>◯ risk related to confusion of the patient<br>◯ wrist band has been lost<br>◯ other
if other, please specify	______________________________
activity monitor collected from patient	◯ Yes<br>◯ No
if not, indicate reason	◯ activity monitor lost<br>◯ activity monitor early withdrawal<br>◯ other
if other, indicate reason	______________________________

Barthel Index at discharge

BI personal hygiene at discharge	◯ The patient is unable to attend to personal hygiene and is dependent in all aspects.<br>◯ Assistance is required in all steps of personal hygiene, but patient able to make some contribution.<br>◯ Some assistance is required in one or more steps of personal hygiene.<br>◯ Patient is able to conduct his/her own personal hygiene but requires minimal assistance before and/or after the operation.<br>◯ The patient can wash his/her hands and face, comb hair, clean teeth and shave. A male patient may use any kind of razor but must insert the blade, or plug in the razor without help, as well as retrieve it from the drawer or cabinet. A female patient must apply her own make-up, if used, but need not braid or style her hair.
BI bathing at discharge	◯ Total dependence in bathing self.<br>◯ Assistance is required in all aspects of bathing, but patient is able to make some contribution.<br>◯ Assistance is required with either transfer to shower/bath or with washing or drying; including inability to complete a task because of condition or disease, etc.<br>◯ Supervision is required for safety in adjusting the water temperature, or in the transfer.<br>◯ The patient may use a bathtub, a shower, or take a complete sponge bath. The patient must be able to do all the steps of whichever method is employed without another person being present.
BI feeding at discharge	◯ Dependent in all aspects and needs to be fed, nasogastric needs to be administered.<br>◯ Can manipulate an eating device, usually a spoon, but someone must provide active assistance during the meal.<br>◯ Able to feed self with supervision. Assistance is required with associated tasks such as putting milk/sugar into tea, salt, pepper, spreading butter, turning a plate or other "set up" activities.<br>◯ Independence in feeding with prepared tray, except may need meat cut, milk carton opened or jar lid etc. The presence of another person is not required.<br>◯ The patient can feed self from a tray or table when someone puts the food within reach. The patient must put on an assistive device if needed, cut food, and if desired use salt and pepper, spread butter, etc.

BI toilet transfers at discharge	◯ Fully dependent in toileting.<br>◯ Assistance required in all aspects of toileting.<br>◯ Assistance may be required with management of clothing, transferring, or washing hands.<br>◯ Supervision may be required for safety with normal toilet. A commode may be used at night but assistance is required for emptying and cleaning.<br>◯ The patient is able to get on/off the toilet, fasten clothing and use toilet paper without help. If necessary, the patient may use a bed pan or commode or urinal at night, but must be able to empty it and clean it.
Bi stair climbing at discharge	◯ The patient is unable to climb stairs.<br>◯ Assistance is required in all aspects of chair climbing, including assistance with walking aids.<br>◯ The patient is able to ascend/descend but is unable to carry walking aids and needs supervision and assistance.<br>◯ Generally no assistance is required. At times supervision is required for safety due to morning stiffness, shortness of breath, etc.<br>◯ The patient is able to go up and down a flight of stairs safely without help or supervision. The patient is able to use hand rails, cane or crutches when needed and is able to carry these devices as he/she ascends or descends.
BI dressing at discharge	◯ The patient is dependent in all aspects of dressing and is unable to participate in the activity.<br>◯ The patient is able to participate to some degree, but is dependent in all aspects of dressing.<br>◯ Assistance is needed in putting on, and/or removing any clothing.<br>◯ Only minimal assistance is required with fastening clothing such as buttons, zips, bra, shoes, etc.<br>◯ The patient is able to put on, remove, corset, braces, as prescribed.
BI bowel control at discharge	◯ The patient is bowel incontinent.<br>◯ The patient needs help to assume appropriate position, and with bowel movement facilitatory techniques.<br>◯ The patient can assume appropriate position, but cannot use facilitatory techniques or clean self without assistance and has frequent accidents. Assistance is required with incontinence aids such as pad, etc.<br>◯ The patient may require supervision with the use of suppository or enema and has occasional accidents.<br>◯ The patient can control bowels and has no accidents, can use suppository, or take an enema when necessary.

BI bladder control at discharge	◯ The patient is dependent in bladder management, is incontinent, or has indwelling catheter.<br>◯ The patient is incontinent but is able to assist with the application of an internal or external device.<br>◯ The patient is generally dry by day, but not at night and needs some assistance with the devices.<br>◯ The patient is generally dry by day and night, but may have an occasional accident or need minimal assistance with internal or external devices.<br>◯ The patient is able to control bladder day and night, and/or is independent with internal or external devices.
BI ambulation at discharge	◯ Dependent in ambulation.<br>◯ Constant presence of one or more assistant is required during ambulation.<br>◯ Assistance is required with reaching aids and/or their manipulation. One person is required to offer assistance.<br>◯ The patient is independent in ambulation but unable to walk 50 metres without help, or supervision is needed for confidence or safety in hazardous situations.<br>◯ The patient must be able to wear braces if required, lock and unlock these braces assume standing position, sit down, and place the necessary aids into position for use. The patient must be able to crutches, canes, or a walkarette, and walk 50 metres without help or supervision.
if dependent in ambulation, has the patient been trained in wheelchair management?	◯ Yes<br>◯ No
if unable to walk and trained in wheelchair management, BI ambulation wheelchair	◯ Dependent in wheelchair ambulation.<br>◯ Patient can propel self short distances on flat surface, but assistance is required for all other steps of wheelchair management.<br>◯ Presence of one person is necessary and constant assistance is required to manipulate chair to table, bed, etc.<br>◯ The patient can propel self for a reasonable duration over regularly encountered terrain. Minimal assistance may still be required in "tight corners" or to negotiate a kerb 100mm high.<br>◯ To propel wheelchair independently, the patient must be able to go around corners, turn around, manoeuvre the chair to a table, bed, toilet, etc. The patient must be able to push a chair at least 50 metres and negotiate a kerb.

BI chair/bed transfers at discharge	◯ Unable to participate in a transfer. Two attendants are required to transfer the patient with or without a mechanical device.<br>◯ Able to participate but maximum assistance of one other person is require in all aspects of the transfer.<br>◯ The transfer requires the assistance of one other person. Assistance may be required in any aspect of the transfer.<br>◯ The presence of another person is required either as a confidence measure, or to provide supervision for safety.<br>◯ The patient can safely approach the bed walking or in a wheelchair, lock brakes, lift footrests, or position walking aid, move safely to bed, lie down, come to a sitting position on the side of the bed, change the position of the wheelchair, transfer back into it safely and/or grasp aid and stand. The patient must be independent in all phases of this activity.
The Barthel Index is not complete yet!	________________________________
Barthel Index	__________________________

Substudy form 2: hypothetical inclusion in future VTE RCT

Eligibility for substudy as determined at the baseline visit: [d0_enrolment_and_b_arm_1][rct_eligibility_conf]

This form is not applicable, as the participant is not eligible for the substudy. The discharge visit is now completed, thank you!

patient willing to consent for enrolment in hypothetical randomized trial on VTE prophylaxis	◯ Yes<br>◯ No
if not willing, please indictate the reason for refusal (free text)	__________________________
if willing to provide informed consent, what would be the patient's preferred follow-up visit type and schedule after discharge? (please check any that apply)	☐ inpatient visits<br>☐ phone calls<br>☐ both inpatient visits and phone calls<br>☐ once after 90 days<br>☐ twice within 90 days<br>☐ 3 times within 90 days<br>☐ other suggestions concerning visits and schedule
if other suggestions for follow-up visits or schedule, enter free text	__________________________

The discharge visit is now completed, thank you!

Rehospitalization

rehospitalization since discharge	◯ Yes<br>◯ No<br>(admission to a rehabilitation is NOT considered a rehospitalization)
if yes, date of first rehospitalization	__________________________________<br>(date format dd-mm-yyyy)
if yes, date of second rehospitalization	__________________________________<br>(date format dd-mm-yyyy; if no second rehospitalization date, leave blank)
if yes, date of third rehospitalization	__________________________________<br>(_date format dd-mm-yyyy; if no third rehospitalization date, leave blank)
if yes, date of fourth rehospitalization	__________________________________<br>(date format dd-mm-yyyy; if no fourth rehospitalization date, leave blank)
if yes, date of fifth rehospitalization	__________________________________<br>(_date format dd-mm-yyyy; if no fifth rehospitalization date, leave blank)
if yes, date of sixth rehospitalization	__________________________________<br>(date format dd-mm-yyyy; if no sixth rehospitalization date, leave blank)
if yes, date of seventh rehospitalization	__________________________________<br>(date format dd-mm-yyyy; if no seventh rehospitalization date, leave blank)

Barthel Index_d90

BI personal hygiene at 90 days	◯ The patient is unable to attend to personal hygiene and is dependent in all aspects.<br>◯ Assistance is required in all steps of personal hygiene, but patient able to make some contribution.<br>◯ Some assistance is required in one or more steps of personal hygiene.<br>◯ Patient is able to conduct his/her own personal hygiene but requires minimal assistance before and/or after the operation.<br>◯ The patient can wash his/her hands and face, comb hair, clean teeth and shave. A male patient may use any kind of razor but must insert the blade, or plug in the razor without help, as well as retrieve it from the drawer or cabinet. A female patient must apply her own make-up, if used, but need not braid or style her hair.
BI bathing at 90 days	◯ Total dependence in bathing self.<br>◯ Assistance is required in all aspects of bathing, but patient is able to make some contribution.<br>◯ Assistance is required with either transfer to shower/bath or with washing or drying; including inability to complete a task because of condition or disease, etc.<br>◯ Supervision is required for safety in adjusting the water temperature, or in the transfer.<br>◯ The patient may use a bathtub, a shower, or take a complete sponge bath. The patient must be able to do all the steps of whichever method is employed without another person being present.
BI feeding at 90 days	◯ Dependent in all aspects and needs to be fed, nasogastric needs to be administered.<br>◯ Can manipulate an eating device, usually a spoon, but someone must provide active assistance during the meal.<br>◯ Able to feed self with supervision. Assistance is required with associated tasks such as putting milk/sugar into tea, salt, pepper, spreading butter, turning a plate or other "set up" activities.<br>◯ Independence in feeding with prepared tray, except may need meat cut, milk carton opened or jar lid etc. The presence of another person is not required.<br>◯ The patient can feed self from a tray or table when someone puts the food within reach. The patient must put on an assistive device if needed, cut food, and if desired use salt and pepper, spread butter, etc.

BI toilet transfers at 90 days	◯ Fully dependent in toileting.<br>◯ Assistance required in all aspects of toileting.<br>◯ Assistance may be required with management of clothing, transferring, or washing hands.<br>◯ Supervision may be required for safety with normal toilet. A commode may be used at night but assistance is required for emptying and cleaning.<br>◯ The patient is able to get on/off the toilet, fasten clothing and use toilet paper without help. If necessary, the patient may use a bed pan or commode or urinal at night, but must be able to empty it and clean it.
Bi stair climbing at 90 days	◯ The patient is unable to climb stairs.<br>◯ Assistance is required in all aspects of chair climbing, including assistance with walking aids.<br>◯ The patient is able to ascend/descend but is unable to carry walking aids and needs supervision and assistance.<br>◯ Generally no assistance is required. At times supervision is required for safety due to morning stiffness, shortness of breath, etc.<br>◯ The patient is able to go up and down a flight of stairs safely without help or supervision. The patient is able to use hand rails, cane or crutches when needed and is able to carry these devices as he/she ascends or descends.
BI dressing at 90 days	◯ The patient is dependent in all aspects of dressing and is unable to participate in the activity.<br>◯ The patient is able to participate to some degree, but is dependent in all aspects of dressing.<br>◯ Assistance is needed in putting on, and/or removing any clothing.<br>◯ Only minimal assistance is required with fastening clothing such as buttons, zips, bra, shoes, etc.<br>◯ The patient is able to put on, remove, corset, braces, as prescribed.
BI bowel control at 90 days	◯ The patient is bowel incontinent.<br>◯ The patient needs help to assume appropriate position, and with bowel movement facilitatory techniques.<br>◯ The patient can assume appropriate position, but cannot use facilitatory techniques or clean self without assistance and has frequent accidents. Assistance is required with incontinence aids such as pad, etc.<br>◯ The patient may require supervision with the use of suppository or enema and has occasional accidents.<br>◯ The patient can control bowels and has no accidents, can use suppository, or take an enema when necessary.

BI bladder control at 90 days	◯ The patient is dependent in bladder management, is incontinent, or has indwelling catheter.<br>◯ The patient is incontinent but is able to assist with the application of an internal or external device.<br>◯ The patient is generally dry by day, but not at night and needs some assistance with the devices.<br>◯ The patient is generally dry by day and night, but may have an occasional accident or need minimal assistance with internal or external devices.<br>◯ The patient is able to control bladder day and night, and/or is independent with internal or external devices.
BI ambulation at 90 days	◯ Dependent in ambulation.<br>◯ Constant presence of one or more assistant is required during ambulation.<br>◯ Assistance is required with reaching aids and/or their manipulation. One person is required to offer assistance.<br>◯ The patient is independent in ambulation but unable to walk 50 metres without help, or supervision is needed for confidence or safety in hazardous situations.<br>◯ The patient must be able to wear braces if required, lock and unlock these braces assume standing position, sit down, and place the necessary aids into position for use. The patient must be able to crutches, canes, or a walkarette, and walk 50 metres without help or supervision.
if dependent in ambulation, has the patient been trained in wheelchair management?	◯ Yes<br>◯ No
if unable to walk and trained in wheelchair management, BI ambulation wheelchair	◯ Dependent in wheelchair ambulation.<br>◯ Patient can propel self short distances on flat surface, but assistance is required for all other steps of wheelchair management.<br>◯ Presence of one person is necessary and constant assistance is required to manipulate chair to table, bed, etc.<br>◯ The patient can propel self for a reasonable duration over regularly encountered terrain. Minimal assistance may still be required in "tight corners" or to negotiate a kerb 100mm high.<br>◯ To propel wheelchair independently, the patient must be able to go around corners, turn around, manoeuvre the chair to a table, bed, toilet, etc. The patient must be able to push a chair at least 50 metres and negotiate a kerb.

BI chair/bed transfers at 90 days	◯ Unable to participate in a transfer. Two attendants are required to transfer the patient with or without a mechanical device.<br>◯ Able to participate but maximum assistance of one other person is require in all aspects of the transfer.<br>◯ The transfer requires the assistance of one other person. Assistance may be required in any aspect of the transfer.<br>◯ The presence of another person is required either as a confidence measure, or to provide supervision for safety.<br>◯ The patient can safely approach the bed walking or in a wheelchair, lock brakes, lift footrests, or position walking aid, move safely to bed, lie down, come to a sitting position on the side of the bed, change the position of the wheelchair, transfer back into it safely and/or grasp aid and stand. The patient must be independent in all phases of this activity.
The Barthel Index is not complete yet!	
Barthel Index	______________________________

Medications since discharge

start of therapeutic anticoagulation since discharge	◯ Yes<br>◯ No
if yes, indicate date when anticoagulation was started	__________________________________<br>(date format dd-mm-yyyy; leave blank if unknown)
if yes, indicate reason	◯ venous thromboembolism<br>◯ atrial fibrillation<br>◯ prosthetic valve<br>◯ other
if other, indicate reason (free text)	__________________________________

Thank you, you have now completed data collection for the follow-up visit. Don't forget to complete end of study form!

End of study

Patient has completed the study according to protocol?	◯ Yes ◯ No<br>(if follow-up phone call was late (e.g. due to invavilability of the patient), the study is still considered as completed according to protocol)
Completion Date	______________________<br>(date format dd-mm-yyyy)
Reason for non-completion	◯ Patient withdrew consent<br>◯ Patient was lost to follow-up<br>◯ Patient was excluded requested by PI<br>◯ Patient died<br>◯ Other reason
State a reason why patient withdrew consent	◯ adverse event<br>◯ pain<br>◯ general wellbeing<br>◯ not willing to dedicate more time to the study<br>◯ other
If other, please specify	______________________
if withdrawal: agrees for passive follow-up	◯ Yes<br>◯ No<br>(passive follow-up refers to collection of follow-information based on available hospital records and/or follow-up with the patient's primary care physician)
Date of last contact	______________________<br>(date format dd-mm-yyyy)
Death date	______________________<br>(date format dd-mm-yyyy)
Cause of death	◯ pulmonary embolism<br>◯ bleeding<br>◯ acute coronary syndrome<br>◯ ischemic stroke<br>◯ left ventricular cardiac failure<br>◯ sudden death without known etiology (necropsy recommended)<br>◯ cancer<br>◯ unknown<br>◯ other
if other, please specify	______________________
Please provide reason	______________________

Please collect medical records, death certificates and autopsy reports.

Adjudication

Date of patient inclusion (i.e. date of informed consent): [d0_enrolment_and_b_arm_1][ic_date]	
date of event	________________________
type of event	◯ vte event<br>◯ bleeding<br>◯ death
hospital acquired VTE	☐ time between hospital admission and VTE event ≥48h<br>(check if any apply, otherwise leave empty)
symptoms of pulmonary embolism	☐ symptoms consistent with PE (new/worsening dyspnea or (bloody) cough, acute chest pain, syncope)<br>(check if any apply, otherwise leave empty)
objective diagnosis of PE	☐ objectively confirmed PE based on available radiographic reports, defined as a new intraluminal filling defect on CTPA or pulmonary angiography<br>☐ objectively confirmed PE based on available radiographic reports, defined as a ventilation-perfusion lung scan showing a new perfusion defect involving at least 75% of a segment, with corresponding normal ventilation (i.e., high probability lung scan)<br>☐ the confirmation of a new PE on autopsy<br>(check if any apply, otherwise leave empty)
symptoms of DVT	☐ symptoms consistent with DVT (unilateral pain or swelling or erythema)<br>(check if any apply, otherwise leave empty)
objective diagnosis of proximal leg DVT	☐ non-compressibility of a proximal (i.e. popliteal vein or higher) venous segment on lower limb compression ultrasonography<br>☐ a proximal (i.e. popliteal vein or higher) intraluminal filling defect on contrast venography of the lower limbs<br>☐ abnormal duplex flow patterns compatible with thrombosis or an intraluminal filling defect on spiral computed tomography or magnetic resonance imaging venography in the iliac and/or caval vein<br>☐ the confirmation of a proximal (i.e. popliteal vein or higher) leg DVT on autopsy<br>(check if any apply, otherwise leave empty)

objective diagnosis of distal leg DVT	☐ non-compressibility of a distal (i.e. infrapopliteal) venous segment on lower limb compression ultrasonography with a diameter of the non-compressible vein of ≥5mm<br>☐ distal (i.e. infrapopliteal) intraluminal filling defect on lower limb contrast venography<br>☐ the confirmation of a distal (i.e. infrapopliteal) leg DVT on autopsy<br>(check if any apply, otherwise leave empty)
objective diagnosis of upper extremity DVT	☐ non-compressibility of a venous segment of the subclavian, axillary, innominate (brachiocephalic), internal jugular, humeral (or brachial), ulnar or radial vein on compression ultrasonography<br>☐ an intraluminal filling defect in the subclavian, axillary, innominate (brachiocephalic), internal jugular, humeral (or brachial), ulnar or radial vein on venography<br>☐ abnormal duplex flow patterns compatible with thrombosis or an intraluminal filling defect on spiral computed tomography or magnetic resonance imaging venography in the subclavian vein<br>☐ the confirmation of an upper extremity DVT (the subclavian, axillary, innominate (brachiocephalic), internal jugular, humeral (or brachial), ulnar or radial vein) on autopsy<br>(check if any apply, otherwise leave empty)
Adjudicated VTE event	◯ hospital-acquired symtpomatic PE<br>◯ hospital-acquired symptomatic proximal lower limb DVT<br>◯ hospital-acquired symptomatic distal lower limb DVT<br>◯ hospital-acquired symptomatic upper extremity DVT<br>◯ this event is no hospital-acquired symptomatic VTE event according to the definition of the study
location of pulmonary embolism	◯ central, lobar, segmental pulmonary artery<br>◯ subsegmental pulmonary artery<br>(indicate according to most proximal location)
this event describes a bleeding event	◯ Yes<br>◯ No
major bleeding criteria	☐ Fatal bleeding (please fill in the death adjudication form also)<br>☐ Bleeding at a critical site<br>☐ Bleeding led to a reduction of hemoglobin of at least 20 g/L<br>☐ Bleeding lead to transfusion of 2 or more units of packed red blood cells<br>(check if any apply, otherwise leave empty)

site of critical bleeding	☐ Intracranial<br>☐ Intraspinal<br>☐ Intraocular<br>☐ Retroperitoneal<br>☐ Intraarticular<br>☐ Pericardial<br>☐ Intramuscular with compartment syndrome
the bleeding event was associated with	☐ medical intervention / happened during hospitalization<br>☐ unscheduled physician contact (visit or telephone call)<br>☐ pain<br>☐ impairment of activities of daily life<br>(check if any apply, otherwise leave empty)
Adjudicated bleeding event	◯ no bleeding event<br>◯ major bleeding<br>◯ clinically significant non-major bleeding<br>◯ minor bleeding event
cause of death	◯ pulmonary embolism<br>◯ major bleeding<br>◯ other cause<br>◯ undetermined cause
indicate the criteria for death due to PE	◯ autopsy-confirmed PE in the absence of another more likely cause of death<br>◯ objectively confirmed PE within the last 48 hours before death (see definition for new PE) in the absence of another more likely cause of death<br>◯ PE is not objectively confirmed, but is most likely the main cause of death
type of fatal bleeding	◯ intracranial<br>◯ other major bleeding
please specify	◯ acute coronary syndrome<br>◯ ischemic stroke<br>◯ infection/ sepsis<br>◯ trauma<br>◯ suicide<br>◯ left ventricular failure<br>◯ respiratory failure (other than PE)<br>◯ cancer<br>◯ kidney failure<br>◯ fatal arrhythmia<br>◯ dissecting aneurysm<br>◯ other
please specify	__________________________________

if the patient had more than one adjudicated medical outcome event, please select "Save & go to next instance" to enter the adjudication data of the additional outcome event
