# Supplementary material for: Risk stratification for hospital-acquired venous thromboembolism in medical patients (RISE): Protocol for a prospective cohort study
Source: PLoS One. 2022 May 24;17(5):e0268833. doi: 10.1371/journal.pone.0268833 (PMC9128957; doi:10.1371/journal.pone.0268833)
Supplement: S5 File — (PDF) [file pone.0268833.s005.pdf]

Adjudication Form RISE: VTE Events

First and last name of adjudicator: ____

Patient-ID: ____

Date of patient's inclusion [ ] dd.mm.yyyy

Date of VTE event: [ ] dd.mm.yyyy

T **hospital-acquired VTE:**

☐ time between index hospital admission and VTE event ≥48h[1]

A **symptoms of pulmonary embolism:**

☐ symptoms consistent with PE (new/worsening dyspnea or (bloody) cough, acute chest pain, syncope)

B **objective diagnosis of PE** (no answer or multiple answers possible)

☐ objectively confirmed PE based on available radiographic reports, defined as a new intraluminal filling defect on CTPA or pulmonary angiography

☐ objectively confirmed PE based on available radiographic reports, defined as a ventilation-perfusion lung scan showing a new perfusion defect involving at least 75% of a segment, with corresponding normal ventilation (i.e., high probability lung scan)

☐ the confirmation of a new PE on autopsy

C **symptoms of DVT:**

☐ symptoms consistent with DVT (unilateral pain or swelling or erythema)[4]

D **objective diagnosis of proximal leg DVT** (no answer or multiple answers possible)

☐ non-compressibility of a proximal (i.e. popliteal vein or higher) venous segment on lower limb compression ultrasonography

☐ a proximal (i.e. popliteal vein or higher) intraluminal filling defect on contrast venography of the lower limbs

☐ abnormal duplex flow patterns compatible with thrombosis or an intraluminal filling defect on spiral computed tomography or magnetic resonance imaging venography in the iliac and/or caval vein

☐ the confirmation of a proximal (i.e. popliteal vein or higher) leg DVT on autopsy

---

E **objective diagnosis of distal leg DVT** (no answer or multiple answers possible)

☐ non-compressibility of a distal (i.e. infrapopliteal) venous segment on lower limb compression ultrasonography with a diameter of the non-compressible vein of ≥5mm

☐ distal (i.e. infrapopliteal) intraluminal filling defect on lower limb contrast venography

☐ the confirmation of a distal (i.e. infrapopliteal) leg DVT on autopsy

---

F **objective diagnosis of upper extremity DVT** (no or multiple answers possible) [2-4]

☐ non-compressibility of a venous segment of the subclavian, axillary, innominate (brachiocephalic), internal jugular, humeral (or brachial), ulnar or radial vein on compression ultrasonography

☐ an intraluminal filling defect in the subclavian, axillary, innominate (brachiocephalic), internal jugular, humeral (or brachial), ulnar or radial vein on venography

☐ abnormal duplex flow patterns compatible with thrombosis or an intraluminal filing defect on spiral computed tomography or magnetic resonance imaging venography in the subclavian vein

☐ the confirmation of an upper extremity DVT (the subclavian, axillary, innominate (brachiocephalic), internal jugular, humeral (or brachial), ulnar or radial vein) on autopsy

---

○ A plus B plus T OR A plus D plus T: hospital-acquired symptomatic pulmonary embolism (PE)[5]

Localization: □ central □ lobar □ segmental pulmonary artery □ subsegmental artery

○ C plus D plus T: hospital-acquired symptomatic proximal lower limb DVT[5]

○ C plus E plus T: hospital-acquired symptomatic distal lower limb DVT[5]

○ C plus F plus T: hospital-acquired symptomatic upper extremity DVT[6]

○ neither T AND (A plus B) OR (A plus D) OR (C plus D) OR (C plus E) OR (C plus F): this event is no hospital-acquired symptomatic VTE event according to the definition of the study

---

1. Roy PM, Rachas A, Meyer G, et al. Multifaceted Intervention to Prevent Venous Thromboembolism in Patients Hospitalized for Acute Medical Illness: A Multicenter Cluster-Randomized Trial. PloS one. 2016;11(5):e0154832.
2. Spyropoulos A, Ageno W, Albers GW, et al. Rivaroxaban for Thromboprophylaxis after Hospitalization for Medical Illness. The New England journal of medicine. 2018;379(12):1118-1127
3. Newton DH, Monreal Bosch M, Amendola M, et al. Analysis of non-catheter-associated upper extremity deep venous thrombosis from the RIETE registry. J Vasc Surg Venous Lymphat Disord. 2017;5(1):18-24.e1
4. Ploton G, Pistoriums MA, Raimbeau A, et al. A STROBE cohort study of 755 deep and supervficial upper-extremity vein thrombosis. Medicine (Baltimore) 2020 Feb;99(6):e18996.
5. Mean M, Righini M, Jaeger K, et al. The Swiss cohort of elderly patients with venous thromboembolism (SWITCO65+): rationale and methodology. J Thromb Thrombolysis. 2013;36(4):475-483.
6. Cote LP, Greenberg S, Caprini JA, et al. Comparisons Between Upper and Lower Extremity Deep Vein Thrombosis: A Review of the RIETE Registry. Clinical and applied thrombosis/hemostasis : official journal of the International Academy of Clinical and Applied Thrombosis/Hemostasis. 2017;23(7):748-754.

Adjudication Form RISE: Bleeding Events

First and last name of adjudicator: ______________________________

Patient-ID: ______________________________

Date of patient's inclusion: [ ] dd.mm.yyyy

Date of bleeding event: [ ] dd.mm.yyyy

A (no answer or one answer possible)

☐ **Bleeding event**

B (no answer or multiple answers possible)

☐ **Fatal bleeding** (please fill in the death adjudication form also)

death is considered to be bleeding-related if it follows an intracranial hemorrhage or a bleeding episode leading to hemodynamic deterioration (needing fluid resuscitation or needing perfusion of amines or intervention prior to death)[1]

☐ **Bleeding at a critical site** if

- □ Intracranial
- □ Intraspinal
- □ Intraocular
- □ Retroperitoneal
- □ Intraarticular
- □ Pericardial
- □ Intramuscular with compartment syndrome

☐ **Reduction of hemoglobin of at least 20 g/L**

the reduction of hemoglobin of ≥20g/l relates to the difference between the latest hemoglobin value before the bleeding and the lowest value within 24 hours after bleeding[2]

☐ **Leading to transfusion of 2 or more units of packed red blood cells**

C (no answer or multiple answers possible)

☐ **bleeding event associated with**

- □ medical intervention / happened during hospitalization
- □ unscheduled physician contact (visit or telephone call)
- □ pain
- □ impairment of activities of daily life

○ **none of the above: no bleeding event**

○ **A plus B: major bleeding**[3]

○ **A plus C, but NOT B: clinically significant non-major bleeding**[4]

○ **A, but not B or C: minor bleeding event**

---

1. Jakobsson C, Jimenez D, Gomez V, et al. *J Thromb Haemost.*8(6):1242-1247.
2. Raskob GE, Spyropoulos AC, Zrubek J, et al. *Thromb Haemost.* 2016;115(6):1240-1248.
3. Schulman S, Kearon C. *J Thromb Haemost.* 2005;3(4):692-694.
4. Buller HR, Prins MH, Lensin AW, et al. *N Engl J Med.* 2012;366(14):1287-1297

Adjudication Form RISE: Cause of Death

First and last name of adjudicator: ______________________

Patient-ID: ______________________

Date of patient's inclusion: [ ] dd.mm.yyyy

Date of death: [ ] dd.mm.yyyy

Please indicate the cause of death:

○ pulmonary embolism (PE)[1]

○ autopsy-confirmed PE in the absence of another more likely cause of death

○ objectively confirmed PE within the last 48 hours before death (see definition for new PE) in the absence of another more likely cause of death

○ PE is not objectively confirmed, but is most likely the main cause of death

○ major bleeding

death is considered to be bleeding-related if it follows an intracranial hemorrhage or a bleeding episode leading to hemodynamic deterioration[2]

○ **intracranial**

○ **other major bleeding**

○ other cause, any death not covered by the above definitions, please specify :[3,4]

○ acute coronary syndrome ○ left ventricular failure ○ fatal arrhythmia

○ ischemic stroke ○ dissecting aneurysm

○ infection / sepsis ○ cancer ○ respiratory failure (other than PE) ○ kidney failure

○ trauma ○ suicide ○ other, please specify :

○ undetermined

if the cause of death is undetermined based upon the clinical information, or if no or insufficient information is available to determine the cause of death

---

1. Tritschler T, Kraaijpoel N, Girard P, et al. Definition of pulmonary embolism-related death and classification of the cause of death in venous thromboembolism studies: communication from the SSC of the ISTH. J Thromb Haemost.
2.Jakobsson C, et al. Validation of a clinical algorithm to identify low-risk patients with pulmonary embolism. J Thromb Haemost.8(6):1242-1247.
3. World Health Organization. Manual of the international statistical classification of diseases, injury, and causes of death. Geneva. 1977.
4. Cutlip DE, et al. Clinical end points in coronary stent trials: a case for standardized definitions. Circulation. 2007;115(17):2344-2351.
