# Supplementary material for: Risk stratification for hospital-acquired venous thromboembolism in medical patients (RISE): Protocol for a prospective cohort study
Source: PLoS One. 2022 May 24;17(5):e0268833. doi: 10.1371/journal.pone.0268833 (PMC9128957; doi:10.1371/journal.pone.0268833)
Supplement: Attachment — Submitted filename: Response to Reviewers.docx [file pone.0268833.s007.docx]

Roza Chaireti
Academic Editor
PLOS ONE

Lausanne, April 5^th^, 2022

**Re: Revision of the manuscript entitled “RIsk Stratification for hospital-acquired venous thromboEmbolism in medical patients (RISE): protocol for a prospective cohort study.”**

Dear editors,

Thank you for the opportunity to revise and resubmit our manuscript entitled “RIsk Stratification for hospital-acquired venous thromboEmbolism in medical patients (RISE): protocol for a prospective cohort study.”

We have provided a point-by-point response to each comment below.

Each author has approved the revised version, and we hope that these changes meet with your approval.

Please contact Dr. Choffat at [damien.choffat@chuv.ch](mailto:damien.choffat@chuv.ch) with any remaining questions or for additional clarification.

Thank you again for your time and consideration.

Sincerely,

Dr Damien Choffat

1. ***Please ensure that your manuscript meets PLOS ONE's style requirements, including those for file naming. The PLOS ONE style templates can be found at***
   [***https://journals.plos.org/plosone/s/file?id=wjVg/PLOSOne_formatting_sample_main_body.pdf***](https://journals.plos.org/plosone/s/file?id=wjVg/PLOSOne_formatting_sample_main_body.pdf) ***and*** [***https://journals.plos.org/plosone/s/file?id=ba62/PLOSOne_formatting_sample_title_authors_affiliations.pdf***](https://journals.plos.org/plosone/s/file?id=ba62/PLOSOne_formatting_sample_title_authors_affiliations.pdf)

We ensured that our manuscript met the PLOS ONE’s style requirements.

2. ***Thank you for stating the following in the Acknowledgments/ Funding Section of your manuscript:*** *The RISE cohort is funded by several non-profit foundations (SGAIM Foundation, Novartis Biomedical Research Foundation, Swiss Heart Foundation, Chuard Schmidt Foundation, Gottfried und Julia Bangerter Foundation). The funding sources had no role in study design; in the collection, analysis and interpretation of data; in the writing of the report; and in the decision to submit the article for publication.* ***Please note that funding information should not appear in the Acknowledgments section or other areas of your manuscript. We will only publish funding information present in the Funding Statement section of the online submission form. Please remove any funding-related text from the manuscript and let us know how you would like to update your Funding Statement. Currently, your Funding Statement reads as follows:*** *The RISE cohort is funded by several non-profit foundations (SGAIM Foundation, Novartis Biomedical Research Foundation, Swiss Heart Foundation, Chuard Schmidt Foundation, Gottfried und Julia Bangerter Foundation). The funding sources had no role in study design; in the collection, analysis and interpretation of data; in the writing of the report; and in the decision to submit the article for publication.* ***Please include your amended statements within your cover letter; we will change the online submission form on your behalf.***

We removed any funding information from the manuscript. Our Funding Statement section of the online submission is correct.

3. ***Your ethics statement should only appear in the Methods section of your manuscript. If your ethics statement is written in any section besides the Methods, please delete it from any other section.***

We confirm that our ethics statement is stated in the methods section.

4. ***Please review your reference list to ensure that it is complete and correct. If you have cited papers that have been retracted, please include the rationale for doing so in the manuscript text, or remove these references and replace them with relevant current references. Any changes to the reference list should be mentioned in the rebuttal letter that accompanies your revised manuscript. If you need to cite a retracted article, indicate the article’s retracted status in the References list and also include a citation and full reference for the retraction notice.***

We have reviewed the whole bibliography and can confirm that no cited article has been retracted. Furthermore, our bibliography list is complete and correct.

5. ***Does the manuscript provide a valid rationale for the proposed study, with clearly identified and justified research questions? The research question outlined is expected to address a valid academic problem or topic and contribute to the base of knowledge in the field.***
   a. *Reviewer #1: Yes*
   b. *Reviewer #2: Yes*

Thank you very much for your remark.

6. ***Is the protocol technically sound and planned in a manner that will lead to a meaningful outcome and allow testing the stated hypotheses?***

*The manuscript should describe the methods in sufficient detail to prevent undisclosed flexibility in the experimental procedure or analysis pipeline, including sufficient outcome-neutral conditions (e.g. necessary controls, absence of floor or ceiling effects) to test the proposed hypotheses and a statistical power analysis where applicable. As there may be aspects of the methodology and analysis which can only be refined once the work is undertaken, authors should outline potential assumptions and explicitly describe what aspects of the proposed analyses, if any, are exploratory.*

a. *Reviewer #1: Yes*
b. *Reviewer #2: Yes*

Thank you.

7. ***Is the methodology feasible and described in sufficient detail to allow the work to be replicable?*** *Descriptions of methods and materials in the protocol should be reported in sufficient detail for another researcher to reproduce all experiments and analyses. The protocol should describe the appropriate controls, sample size calculations, and replication needed to ensure that the data are robust and reproducible.*
   a. *Reviewer #1: Yes*
   b. *Reviewer #2: Yes*

Thank you very much.

8. ***Have the authors described where all data underlying the findings will be made available when the study is complete?*** *The PLOS Data policy requires authors to make all data underlying the findings described in their manuscript fully available without restriction, with rare exception, at the time of publication. The data should be provided as part of the manuscript or its supporting information, or deposited to a public repository. For example, in addition to summary statistics, the data points behind means, medians and variance measures should be available. If there are restrictions on publicly sharing data—e.g. participant privacy or use of data from a third party—those must be specified.*
   a. *Reviewer #1: No*
   b. *Reviewer #2: No*

This manuscript describes a protocol for a prospective cohort study, which is still ongoing. There are no datasets analyzed for this manuscript. Therefore, we cannot make the data fully available.

9. ***Is the manuscript presented in an intelligible fashion and written in standard English?*** *PLOS ONE does not copyedit accepted manuscripts, so the language in submitted articles must be clear, correct, and unambiguous. Any typographical or grammatical errors should be corrected at revision, so please note any specific errors here.*
   a. *Reviewer #1: Yes*
   b. *Reviewer #2: Yes*

Thank you very much.

10. *PLOS authors have the option to publish the peer review history of their article (*[*what does this mean?*](https://journals.plos.org/plosone/s/editorial-and-peer-review-process#loc-peer-review-history)*). If published, this will include your full peer review and any attached files. If you choose “no”, your identity will remain anonymous but your review may still be made public.* ***Do you want your identity to be public for this peer review?*** *For information about this choice, including consent withdrawal, please see our Privacy Policy.*
    a. *Reviewer #1: No*
    b. *Reviewer #2: No*

**Reviewer #1:**

1. ***The authors presented a protocol for a prospective cohort study on risk stratification for hospital-acquired venous thromboembolism in medical patients. Venous stasis as a predisposing factor for VTE in medical patients has been under recognized, and this RCT will try to shed light on this aspect using objectively measured immobilization.***

Thank you very much for your comment.

2. ***Abstract, methods: you state “are calculated for each RAM”, but you have not listed or mentioned them. Essentially the scope of this study is to provide an objective assessment and grading of patient immobility, and assess its relative contribution in VTE risk, and eventually incorporate it in existing RAMs.***

Thank you for this remark. Indeed, we initially didn’t specify the name of the RAMs. We have now added the name of all the RAMs we will compare against the simplified Geneva score.

Manuscript modifications (abstract, page 2):

- Time-dependent area under the curve, sensitivity, specificity, and positive and negative predictive values are calculated for each RAM **(i.e. the original Geneva score, Padua, IMPROVE score and simplified Geneva score)** with and without the objective mobility measures to assess their accuracy in predicting hospital-acquired VTE at 90 days.

3. ***Page 3, last paragraph: Usually physician mobility orders are used and determine patient mobility; please comment.***

Thank you for this interesting remark. Indeed, many hospitalized patients aren’t active during hospitalization even if they can move freely as shown by this recent study (reference: Tasheva P, et al. Accelerometry assessed physical activity of older adults hospitalized with acute medical illness - an observational study. BMC Geriatr. 2020 Oct 2;20(1):382). Physicians’ mobility orders are not systematically followed by hospitalized patients (please see our manuscript reference #21)

Therefore, we decided not to use physician mobility orders, which represent an ideal goal for patients that is then applied or not by nursing staff and patients themselves, but rather how physicians estimate their patients’ ability to move (i,e, their subjective estimation). Furthermore, we were interested in the physicians’ subjective mobility estimation since it is a key factor in the decision of implementing or not thromboprophylaxis. We now clarified this in the introduction section on page 3.

Manuscript modifications (introduction, page 3):

- Patients and hospital staff also interpret physicians’ orders of mobilization with a substantial variation; for example, ambulation orders “out of bed to chair” can lead to a daily step count of 0 to 1800 (0-1.3 km) [21].

4. ***Page 4, first paragraph: you mention the second aim of your study without mentioning its first aim.***

Thank you for your remark. Indeed, we understand how our phrasing was confusing. We therefore modified our manuscript.

Manuscript modifications (introduction, page 3):

- Therefore, we aim to establish the predictive performance of a novel and objective definition of in-hospital immobilization using accelerometry.

In addition, the aims of the study are mentioned as follows in the Objectives and hypotheses section on page 4:

- “The primary objective is to prospectively validate the simplified Geneva score and to compare its prognostic performance with previously validated RAMs (i.e., the original Geneva, Padua, and IMPROVE scores). Therefore, we hypothesize that the novel, easier-to-use simplified Geneva score will be able to accurately detect medical inpatients at risk of hospital-acquired VTE and that it will be at least as accurate as previously validated RAMs. Our second objective is to develop a new, objective, definition of inpatient immobilization using accelerometry and to compare its performance in predicting hospital-acquired VTE with that of the subjective measurement. Accordingly, we hypothesize that objective, accelerometry-assessed mobility will be more accurate in predicting the risk of hospital-acquired VTE than subjective physician perception and that its incorporation into the simplified Geneva score will improve its prognostic performance.”

**Reviewer #2:**

1. ***In this study authors sent the protocol for a prospective cohort study. The aim of the study was prospectively validate the simplified Geneva score and to examine the predictive performance of a novel and objective definition of in-hospital immobilization using accelerometry. Primary objective is to prospectively validate the simplified Geneva score and to compare its prognostic performance with previously validated RAMs (i.e., the original Geneva, Padua, and IMPROVE scores). Second objective is to develop a new, objective, definition of inpatient immobilization using accelerometry and to compare its performance in predicting hospital acquired VTE with that of the subjective measurement. The work is well written, and the study design is quite robust, however, I would like to make a couple of comments/suggestions:***

Thank you very much for your comment.

2. ***In the estimation of the sample size, “a 90-day incidence of hospital-acquired VTE of respectively 2.8% and 0.6%, in high risk and low risk patients respectively, has been taken into account”. In this sense, the percentages shown refer to percentages of VTE diagnosed with or without thromboprophylaxis? Has the number of patients who will receive thromboprophylaxis been taken into consideration? It is true that the authors comment that a sensitivity analysis adjusted for the use of thromboprophylaxis will be performed, but if this situation has not been previously taken into account, it could influence the statistical power.***

Thank you for this important comment. The incidence of VTE is calculated taking into account the provision of thromboprophylaxis. For clearer comprehension, we modified the sentence on page 12 as follows:

*“Based on a 2010 Swiss cohort study (13), we assume that that 67% of patients are categorized as high risk and 33% as low risk based on the simplified Geneva score and a 90-day incidence of hospital-acquired VTE with adequate thromboprophylaxis of 2.8% and 0.6%, in high risk and low risk patients, respectively, according to the simplified Geneva score (13). Therefore, we will need 1308 patients to detect an absolute risk difference of 2.2% between high and low risk patients, with a power of 80% at a 2-sided alpha of 0.05”*

3. ***Page 10: the authors state that: the objective diagnosis of PE is based on available radiographic or autopsy reports (39-42). I don't know if I have come to understand this sentence, but the diagnosis of PE should be based on a CT-scan /angioCT***

Indeed, we agree with the reviewer that this is confusing. We now modified the description on page 10:

*“[…] the objective diagnostic of PE is based on available radiology (CT pulmonary angiography, pulmonary angiography, or ventilation-perfusion lung scan) or autopsy reports”.*
